# Tin Halide Perovskites: From Fundamental Properties to Solar Cells

**DOI:** 10.1002/adma.202105844

**Published:** 2021-10-28

**Authors:** Matteo Pitaro, Eelco Kinsa Tekelenburg, Shuyan Shao, Maria Antonietta Loi

**Affiliations:** ^1^ Photophysics and OptoElectronics Zernike Institute for Advanced Materials University of Groningen Nijenborgh 4 Groningen 9747 AG The Netherlands

**Keywords:** electronic properties, optical properties, perovskite structures, solar cells, tin‐based perovskites

## Abstract

Metal halide perovskites have unique optical and electrical properties, which make them an excellent class of materials for a broad spectrum of optoelectronic applications. However, it is with photovoltaic devices that this class of materials has reached the apotheosis of popularity. High power conversion efficiencies are achieved with lead‐based compounds, which are toxic to the environment. Tin‐based perovskites are the most promising alternative because of their bandgap close to the optimal value for photovoltaic applications, the strong optical absorption, and good charge carrier mobilities. Nevertheless, the low defect tolerance, the fast crystallization, and the oxidative instability of tin halide perovskites currently limit their efficiency. The aim of this review is to give a detailed overview of the crystallographic, photophysical, and optoelectronic properties of tin‐based perovskite compounds in their multiple forms from 3D to low‐dimensional structures. At the end, recent progress in tin‐based perovskite solar cells are reviewed, mainly focusing on the detail of the strategies adopted to improve the device performances. For each subtopic, the current challenges and the outlook are discussed, with the aim to stimulate the community to address the most important issues in a concerted manner.

## Graphical abstract

A thorough overview is given on the fundamental properties of tin halide perovskites tailored to their application in solar cells. A detailed discussion of the crystal structure, electronic properties, photophysics, and thin‐film crystallization of tin halide perovskites provides a foundation to highlight its exceptional properties and reveals research strategies to improve the performance of tin halide solar cells.

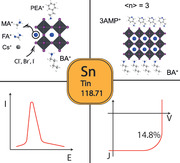

## 1 Introduction

In the past ten years, metal halide perovskites have attracted great interest from the scientific community leading to numerous studies elucidating the physical properties of the material and developing a large variety of optoelectronic devices (solar cells, photodetectors (including x‐ray detectors), light emitting diodes, lasers).^[^ ^1^, ^2^, ^3^ ^]^ Metal halide perovskite, the bulk 3D form, has the general chemical formula ABX_3_, where A is an organic or inorganic monovalent cation (typically methylammonium, formamidinium or cesium), B is a divalent metal ion (Sn^2+^, Ge^2+^, Pb^2+^), and X is a halide anion (Cl^−^, Br^−^, or I^−^). Significant progress has been made not only in developing new perovskite materials and controlling their deposition, as well as in understanding the fundamental crystallographic, optical, and electrical properties. Thanks to these efforts perovskite solar cells (PSCs) showed a big leap in power conversion efficiency (PCE) from 3.8% to 25.5% within a decade, surpassing in performance copper indium gallium selenide (23,4%), cadmium telluride (CdTe, 22.1%), and multicrystalline silicon‐based solar cells (22.3%).^[^ ^4^, ^5^, ^6^, ^7^, ^8^, ^9^, ^10^, ^11^, ^12^, ^13^, ^14^, ^15^ ^]^ The exceptionally high PCE performances are due to the unique combination of optical and electrical characteristics of the lead‐based compounds, including the high absorption coefficient, the small exciton binding energy, the long carrier diffusion length, the ambipolar charge carrier transport and the high tolerance to defects.^[^ ^16^, ^17^, ^18^ ^]^ Despite these excellent properties, the toxicity of lead (Pb) poses concerns about the possible commercialization of these devices. Consequently, an increasing number of studies is currently dedicated to substitute Pb with other group IVA metals, for example, tin (Sn) and germanium (Ge), or group VA metals such as bismuth (Bi), antimony (Sb), and copper (Cu).^[^ ^19^, ^20^, ^21^, ^22^ ^]^

The exchange of Pb with other metals typically results in a compromise between desirable properties relevant for high‐efficiency solar cells or the stability of the material. For example, Bi‐based double perovskites are very stable in ambient conditions; however, the indirect bandgap of ≈2 eV drastically limits their absorption properties.^[^ ^23^, ^24^ ^]^ When using only one metal cation, the trivalent oxidation state of Bi^3+^ and Sb^3+^ causes the formation of a layered vacant structure, limiting the charge transport of the material.^[^ ^25^ ^]^

When looking at group IVA metals, Ge^2+^ with its 4s^2^ electronic configuration easily loses its lone pair electrons. This behavior leads to a poor chemical stability of Ge‐based perovskites and solar cells.^[^ ^26^ ^]^ Although plagued by a lower stability compared to Bi or Sb, Sn is the most promising candidate to substitute the toxic lead compound owing to excellent optical and electrical properties, especially suitable for solar cell applications. Theoretically, Sn‐based perovskites with an optical bandgap in the range of 1.2–1.4 eV could achieve a PCE around 33%.^[^ ^27^ ^]^ In addition, Sn‐based perovskites display similar or superior electronic and optical properties compared to Pb‐based perovskites, such as higher charge carrier mobilities and long‐lived hot carriers.^[^ ^28^, ^29^, ^30^ ^]^ Hence, Sn‐based perovskites have great potential toward the development of highly efficient solar cells.

Despite these exciting properties, Sn‐based perovskite solar cells reached significant lower efficiency of 14.81% compared to the 25.5% of Pb‐based compounds.^[^ ^5^, ^31^ ^]^ This discrepancy is due to the poor stability of Sn‐based perovskites, the instability of the oxidation number 2+ and therefore the fast oxidation of Sn^2+^ in Sn^4+^, which results in large doping concentrations and limits the solar cell efficiency and reproducibility.^[^ ^32^, ^33^ ^]^ Moreover, compact and pinhole‐free films are difficult to obtain. Sn‐based perovskite thin films typically show poor crystalline quality and rough morphologies, with small grains of hundreds of nanometers instead of hundreds of micrometers as for Pb‐based films.^[^ ^34^ ^]^

**Figure 1:**
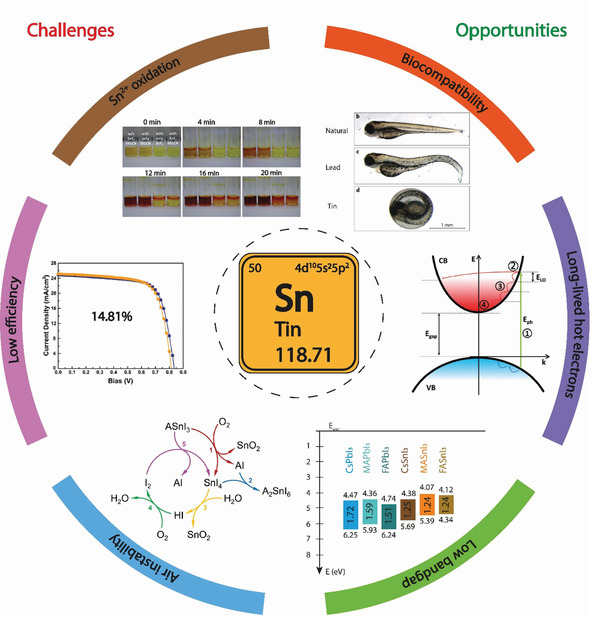
Summary of the opportunities and challenges for Sn‐based tin perovskite materials. Low efficiency. Reproduced with permission.^[^ ^31^ ^]^ Copyright 2021, Wiley‐VCH GmbH. Sn^2+^ oxidation. Reproduced with permission.^[^ ^35^ ^]^ Copyright 2018, The Royal Society of Chemistry. Biocompatibility. Reproduced with permission.^[^ ^36^ ^]^ Copyright 2016, Macmillan Publishers Limited. Long‐lived hot electrons. Reproduced under the terms of the Attribution‐Non‐Commercial 3.0 Unported (CC BY‐NC 3.0).^[^ ^37^ ^]^ Copyright 2019, Royal Society of Chemistry. Low bandgap. Reproduced under the terms of the Creative Common Attribution 4.0 International license.^[^ ^38^ ^]^ Copyright 2019, Nature Research. Air stability. Reproduced under the terms of the Creative Common Attribution 4.0 International license.^[^ ^39^ ^]^ Copyright 2021, Nature Research.

An increased understanding of the properties of Sn‐based perovskites is essential for tackling current challenges that limits the development of this material. **Figure** **1** summarizes both the opportunities provided and the challenges to face in the development of Sn‐based perovskites.

In this review, we critically analyze the physical properties of different types of Sn‐based perovskites and provide a comprehensive overview of the research conducted up until now. As applications, we decided to focus on solar cells; not only because they are the most advanced application of metal halide perovskites, but we believe that in this type of devices the use of Sn‐based perovskites will have the largest impact. First, we shall introduce the general structure and optoelectronic properties of 3D Sn‐based perovskites. Then, we shall discuss new types of Sn‐based perovskites including low‐dimensional systems. We shall then conclude scrutinizing the application of these perovskites in solar cells by analyzing the large body of work present in the literature.

## 2 3D Perovskites

### 2.1 Physical Properties

#### 2.1.1 Structural Properties

The ABX_3_ perovskite structure consists of sixfold coordinated B^2+^ cations surrounded by X sites that form [BX_6_]^4−^ octahedra, while A occupies the cavity formed by the corner shared octahedra. Not all the combinations between cations or anions create a stable 3D structure. For example, the possible dimensions of the A cation are dictated by the dimensions of the B and X ions in order to form the octahedral framework. To indicate which combination between anions and cations realizes a stable perovskite structure, two empirical parameters are used. The first parameter is the geometrical tolerance factor (*t*) defined by the following equation^[^ ^40^ ^]^

(1) $$t=\frac{R_{a}+R_{x}}{\sqrt{2}\left(R_{b}+R_{x}\right)}$$

where *R_a_*, *R_b_*, and *R_x_* are the ionic radii of the A, B, and X sites, respectively. Hence, the tolerance factor assesses whether the A cation can fit within the framework of corner sharing octahedra. Empirical data suggests that *t* values have to be in the range between 0.8 and 1.0 to maintain a 3D perovskite structure. Generally, a tolerance factor between 0.8 and 0.9 shows a distorted structure with rhombohedral, orthorhombic, or tetragonal symmetry; if the values are between 0.9 and 1 an ideal cubic structure can form. MA (radius of 217 pm) and Cs (radius of 167 pm) cations guarantee a stable perovskite phase.^[^ ^41^, ^42^ ^]^ FA (253 pm) is a little bit larger, although slight distortions within the inorganic framework will allow the formation of the perovskite structure.

This parameter‐based approach provides a rapid estimation of the material structure. In fact, the first step to design new perovskites is typically to calculate the tolerance factor, which has been the leading actor in the design of perovskites for over 90 years. However, as reported by Li et al., its accuracy is insufficient, and its deficiency arises from its mathematical formula.^[^ ^43^ ^]^ The predictive power of the tolerance factor is limited, as only 74% of the materials are correctly predicted to be perovskites.^[^ ^44^ ^]^ Bartel et al. reported an improved description using the following equation

(2) $$\tau =\frac{r_{X}}{r_{B}}-n_{A}\left(n_{A}-\frac{\frac{r_{A}}{r_{B}}}{\ln\frac{r_{A}}{r_{B}}}\right)$$

where *n*_A_ is the oxidation state of A, and *r*_i_ is the ionic radius of ion *i*.^[^ ^45^ ^]^ The authors demonstrated an accurate prediction in 92% of the cases. Thanks to its accurate and probabilistic nature as well as its generality, τ is a new key parameter to establish the stability of the perovskite structures and predict new perovskites. Recently, the search for new perovskite compounds was further improved using machine learning modeling and descriptors such as Pauling's rules, atomic packing fractions, and enthalpy of formation.^[^ ^46^, ^47^, ^48^ ^]^

Another important consideration is whether the B site cation can fit in the octahedral cage in the anion sublattice. The value of the B site radius to form an undistorted perovskite structure needs to be greater than 0.41R*_x_*.^[^ ^44^ ^]^ For example, for the oxide and fluoride perovskites, the radius of the octahedral cavity (55 and 52 pm, respectively) limit the number of cations (P^5+^, As^5+^, and Si^4+^) that are suitable to form the structure. To assess the fit of the B site cation into the X_6_ octahedron, a second parameter called the octahedral factor (μ) is defined and is used to estimate the fit of the B cation in the X_6_ octahedron. The octahedral factor is defined as^[^ ^49^ ^]^

(3) $$\mu \;=\frac{R_{b}}{R_{x}}$$

Empirically determined μ values between 0.442 and 0.895 give rise to a perovskite structure.

It is important to note that these two parameters are not the only factors to determine the formability and stability of a perovskite structure, because other nongeometric factors, such as the bond valence and chemical stability should be also considered.

##### Crystal Structure and Phase Transition in 3D Sn‐Based Perovskites

**Figure 2:**
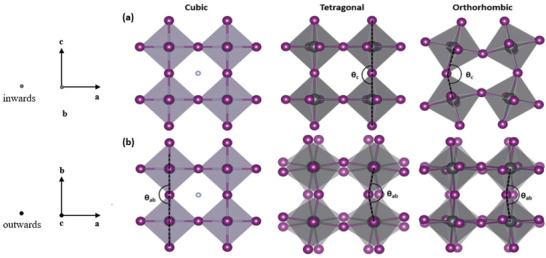
a) Lateral view and b) top view of the cubic, tetragonal, and orthorhombic structure. For the tetragonal structure, the octahedra are rotated such that θ_ab_ < 180°. The orthorhombic structure shows an additional tilting of the octahedra with θ_c_ < 180°.

As mentioned, crystal structures are determined by the types of ions involved, the temperature, the orientation of [BX_6_]^4−^ octahedra, and the B–X–B bond's orientation and length. Among the possible crystal structures, the high‐temperature cubic structure is the most symmetric one and is constructed by the corner‐sharing of the octahedra with B–X–B bond angles of 180°, as shown in **Figure** **2**a. In the empty space of the [BX_6_]^4−^ octahedral framework, the A cations are 12‐fold cuboctahedrally coordinated by the halide anions. In general, the high‐temperature crystal structure transits to a lower symmetric tetragonal or orthorhombic phase upon decreasing temperature. The tetragonal phase is characterized by a rotation of the octahedra resulting in a B–X–B bond angle (θ_ab_) deviating from the ideal 180°, as shown in Figure 2b. An additional distortion is seen in the orthorhombic phase, see Figure 2a, where the angle between two octahedra that do not belong to the same plane (θ_c_) also deviates from 180°.

In the following section, we are going to describe the crystal structure of three different 3D Sn‐based perovskites—FASnI_3_, MASnI_3_, and CsSnI_3_— in detail.

For FASnI_3_, there is not a complete consensus on the crystallographic phase for the analyzed compounds. One of the first studies on the FASnI_3_ crystal structure was carried on by Mitzi and Liang in 1997 and showed a high‐temperature cubic crystal structure.^[^ ^50^ ^]^ They discovered that MASnI_3_ and FASnI_3_ are isostructural, where the formamidinium molecules are orientationally disordered because their symmetry does not agree with O_h_ site symmetry of the cubic perovskite structure. FASnI_3_ shows a Sn—I bond length of 3.158 Å, which is ≈1.2% longer than the perovskite with the MA cation, caused by the steric effect of the larger FA cation.^[^ ^50^ ^]^ At 340 K, FASnI_3_ formed a pseudocubic *Amm2* crystal structure and underwent an orthorhombic phase transition that crystallizes in the polar *Imm2* space group below 180 K with an additional phase transition primitive monoclinic structure that could not be solved close to 100 K to a primitive monoclinic structure that could not be solved.^[^ ^28^ ^]^ By contrast, Schueller et al. determined the first phase transition from cubic *Pm3m* to tetragonal *P4/mbm* at a temperature between 225 and 250 K, cooling from room temperature.^[^ ^51^ ^]^ A further phase transition to an orthorhombic *Pnma* structure occurs between 125 and 150 K. Kahmann et al. observed a transition from a cubic room‐temperature phase to a tetragonal phase at around 255 K and a second tetragonal phase below 155 K instead of the orthorhombic structure reported by Schueller et al.^[^ ^52^ ^]^ The variability in the data reported may be caused by the different nature of the analyzed samples (single crystals or polycrystalline films).

In 2010 Takahashi et al. described the lattice framework of MASnI_3_ and observed a cubic phase at 295 K, while at 275 K they observed a phase transition from the cubic (*Pm3m*) to the lower‐symmetry tetragonal (*I4/mcm*) structure.^[^ ^53^ ^]^ The authors found that the tetragonal structure corresponds to a $\sqrt{2}$ *a* × $\sqrt{2}$ *a* × 2*a* supercell expansion of the cubic lattice due to the SnI_6_ octahedra tilting around the vertical axis. Another phase transition from tetragonal to orthorhombic (*Pbn21* or *Pbnm*) was observed by lowering the temperature to 108–114 K, which was confirmed by Laurita and co‐workers.^[^ ^53^, ^54^ ^]^ Ma et al. studied the crystal structure of MASnI_3_ implementing first‐principles calculations based on density functional theory (DFT) using a Perdew–Burke–Ernzerhof generalized gradient approximation (PBE‐GGA).^[^ ^55^ ^]^ The lattice constant of 6.34 Å calculated with this approach is slightly larger than the experimental value of 6.24 Å due to the overestimation caused by the PBE‐GGA method.^[^ ^53^ ^]^ The authors studied the role of the C—N bond in the MA cations in the tetragonal structure with two different orientations of the C—N bonds—parallel (TETP) and vertical (TETV) to each other—and reported a higher stability for the parallel orientation of the C—N bonds.^[^ ^55^ ^]^

CsSnI_3_ shows four polymorphs: three that show a black color and one that is yellow. Above 425 K, the black cubic phase (B‐α) is formed, which converts to a black tetragonal phase (B‐β) below 426 K and a black orthorhombic phase (B‐γ) at 351 K. Interestingly, in the low‐temperature black phases, the SnI_6_ octahedra do not tilt during the phase transition, even though the Sn–I—Sn bonds are no longer perfectly linear.^[^ ^56^ ^]^ The yellow (Y) phase is characterized by a nonperovskite orthorhombic cell with 1D double‐chain structure and can coexist with the B‐γ orthorhombic *Pnma* space group at room temperature.^[^ ^56^, ^57^ ^]^ At 425 K, the Y phase transitions into the black cubic phase B‐α. Calculations suggested that the unwanted yellow phase could be suppressed by introducing Br and Rb in the structure that simultaneously reduced the oxidation of Sn^2+^ into Sn^4+^, leading to the more stable γ‐Rb*_y_* Cs_1‐_ *_y_* SnI_3_ suitable for solar cell applications.^[^ ^58^ ^]^ The authors also proposed a further stabilization of the perovskite structure by substituting I with Br.

#### 2.1.2 Electronic and Optical Properties

In order to exploit Sn‐based perovskite for optoelectronic applications, it is essential to deeply understand their electronic and optical properties. Evidently, the energy position of the valence and conduction band (VB, CB), the total and partial density of states (DOS), and effective masses, largely determine these properties. In this section we are going to review, both from a theoretical and experimental point of view, what is known about these physical features for MASnI_3_, FASnI_3_, and CsSnI_3_.

##### Band Structure

**Figure 3:**
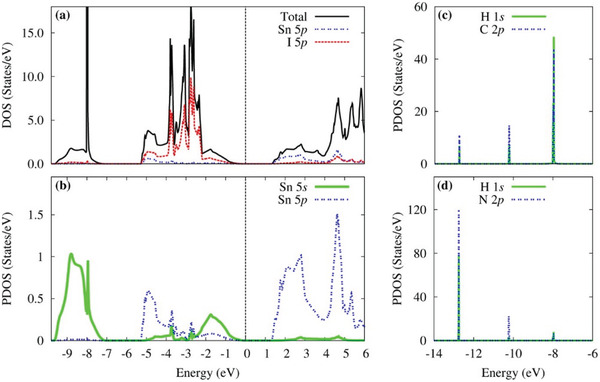
Calculated DOS and PDOS for MASnI_3_. The black dashed line indicates the maximum value of the valence band. Reproduced with permission.^[^ ^60^ ^]^ Copyright 2014, American Chemical Society.

DFT calculations of the band structure of MASnI_3_ using a GW method incorporating spin–orbit coupling show that the cubic phase has a direct bandgap of 1.55 eV at the R point in the Brillouin zone.^[^ ^59^ ^]^ By contrast, the tetragonal and orthorhombic structures have a direct bandgap at the C point. A bandgap of 1.3 eV was calculated using a hybrid functional, which coincided with the experimentally obtained value.^[^ ^60^, ^61^ ^]^ The DOS calculation demonstrates that an antibonding hybridization of the Sn 5s and the I 5p states predominantly contributes to the VBM, while the Sn 5p states and the I 5p states contribute to the conduction band minimum (CBM) (**Figure** **3**a,b).^[^ ^60^ ^]^ Figure 3c,d shows that the organic MA molecules give rise to three discrete energy levels far below the valence band in the energy range from −14 to −7 eV, which means that the MA cations do not contribute directly to the band edges of the VB nor of the CB. Hence, the bandgap of these Sn‐based perovskites is mainly determined by the Sn—X bond. Umari et al. confirmed these results with a relativistic GW approach incorporating spin–orbit coupling (SOC) effects, finding a direct bandgap at the Γ point of the Brillouin zone.^[^ ^59^ ^]^

The carrier effective mass is an important parameter dictating the transport properties. It is calculated from the curvature of the electronic band dispersion at the VBM and CBM, using the following equation

(4) $$m^{*}=\;\frac{\hbar^{2}}{\frac{\partial^{2}E}{\partial k^{2}}}$$

where *E* is the energy dispersion relation and *k* is the lattice wavevector. Although this approach is generally valid, relativistic effects may cause non‐negligible deviation from these calculations in Pb and Sn‐based perovskites. Obviously, theoretically calculated effective masses depend on the band dispersion but also on the applied computational approach in the calculations. Feng and Xiao studied the effects of SOC on the band dispersions and effective mass of charge carriers at the Γ point of the orthorhombic MASnI_3_ under a small isotropic stress using the hybrid functional HSE06.^[^ ^62^ ^]^ **Table** **1** shows these effective masses, which are generally similar to those of orthorhombic MAPbI_3_.^[^ ^63^ ^]^ The switching on of SOC splits the bottom of the conduction band into two separate bands, p_1/2_ and p_3/2_, resulting in a narrower bandgap and an anisotropy in the effective mass of the electrons. The degeneracy for the hole at the top of the valence band mostly remains the same, but the band becomes more dispersive, leading to a smaller effective mass for the holes. Umari et al. highlighted the impact of the different calculation packages on the value of the effective mass (**Table** **2**). The authors propose as best approach a GW method, incorporating spin–orbit coupling, which allowed an accurate modeling of the effective masses.^[^ ^59^ ^]^

**Table 1:** Calculated effective mass of holes and electrons relative to the electron mass *m*_0_ along three different crystallographic directions for MASnI_3_^[^ ^62^ ^]^

Effective mass	[100]	[010]	[001]
$m_{e}^{*}$	3.74	0.81	5.77
$m_{h}^{*}$	0.59	1.14	0.53

**Table 2:** Average value of the electron and the hole effective masses (relative to electron mass *m*_0_) calculated using different approaches: spin–orbit coupling‐GW, spin–orbit coupling‐DFT, and scalar relativistic‐DFT^[^ ^59^ ^]^

Effective mass	SOC‐GW	SOC‐DFT	SR‐DFT
$m_{e}^{*}$ (AVG)	0.28	0.24	0.63
$m_{h}^{*}$ (AVG)	0.13	0.15	0.18

**Figure 4:**
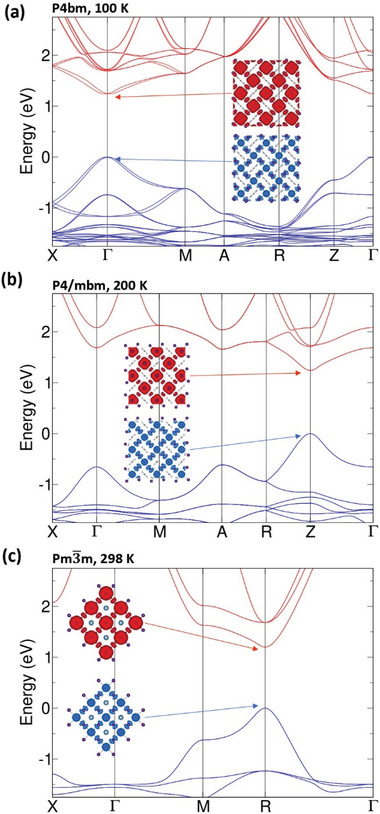
Computed band structures of FASnI_3_ using DFT calculations for the a) 100 K *P4bm*, b) 200 K *P4/mbm*, and c) 298 K *Pm* $\bar{3}$ *m* structures. Reproduced under the terms of the Attribution Non‐Commercial No Derivatives 4.0 International license.^[^ ^52^, ^53^ ^]^ Copyright 2020, American Chemical Society.

Similar to MASnI_3_, FASnI_3_ has a direct bandgap where the VBM and CBM are composed of the same Sn and I orbitals (**Figure** **4**).^[^ ^52^, ^55^ ^]^ Shi et al. found that the Sn—I bond length determines the energy position of the valence band maximum.^[^ ^64^ ^]^ The longer bond length of FASnI_3_ due to the larger ionic size of FA cation weakens the antibonding interactions between I 5p and Sn 5s and lowers the VBM as compared to MASnI_3_. Consequently, FASnI_3_ has a slightly wider bandgap. Tao et al. analyzed the calculated DOS of the tetragonal phase for FASnI_3_ and MASnI_3_, and orthorhombic phase for CsSnI_3_. Comparing the calculated results with experimental data, the authors highlighted the potential weakness of the calculations, which are caused by imperfect surfaces or defects in the film, introducing additional states at the band edges.^[^ ^38^ ^]^ Filippetti et al. used ab initio calculation and absorption measurements to fully investigate electrical and optical properties of FASnI_3_ and MASnI_3_ perovskites.^[^ ^27^ ^]^ The authors found that the comparison between calculated and measured data is complicated by the large defect concentration present in this kind of materials. However, properties not influenced by defects, such as the fundamental bandgap, can be predicted by the calculation method employed with a good quantitative agreement. Roknuzzaman et al. calculated that the electron and hole mass increased upon the substitution of I by Br and Cl, as shown in **Table** **3**.^[^ ^65^ ^]^ Low carrier effective masses imply high charge carrier mobilities, which justify the use of FASnI_3_ as a good candidate for photovoltaic applications. When Sn is replaced by Pb no significant increase was observed, while perovskites with germanium exhibited an increase of the carrier effective masses compared to Sn and Pb compounds.

**Table 3:** Carrier effective mass for electrons $m_{e}^{*}$ and holes $m_{h}^{*}$ in electron mass units for FASnI_3_, FASnBr_3_, and FASnCl_3_^[^ ^65^ ^]^

Materials	$m_{e}^{*}$	$m_{h}^{*}$
FASnI_3_	0.28	0.11
FASnBr_3_	0.39	0.26
FASnCl_3_	0.51	0.35

Interestingly, Ma et al. observed that also the carrier effective masses of MASnX_3_ depend on the halide atoms, organic molecules, and structures of the organic–inorganic halide compounds.^[^ ^55^ ^]^ When the size of halide compound decreases from I to Br, the bandgap of the perovskite material increases (**Table** **4**). The authors also observed an increase in the bandgap when the size of the cation increases from MA to FA, in accordance with the study by Shi et al. Moreover, they observed that replacing Pb with Sn in the perovskite compounds can reduce the bandgap and the carrier effective masses.

**Table 4:** Calculated effective masses and bandgaps of MASnI_3_ and MASnBr_3_^[^ ^55^ ^]^

	MASnI_3_			MASnBr_3_		
	Bandgap [eV]	$m_{e}^{*}$	$m_{h}^{*}$	Bandgap [eV]	$m_{e}^{*}$	$m_{h}^{*}$
Cubic	0.83	0.17	0.11	1.12	0.19	0.14
TETP	0.73	0.11	0.10	1.06	0.17	0.14
TETV	0.93	0.15	0.14	1.36	0.25	0.19
Orthorhombic	1.08	0.19	0.18	1.53	0.22	0.23

Huang and Lambrecht analyzed the band structure of the cubic phase of CsSnI_3_ using DFT calculation with quasiparticle self‐consistent GW (QSGW) method without spin–orbit coupling.^[^ ^66^ ^]^ Similar to the hybrid organic inorganic structure, the VBM consists of antibonding Sn 5s and I 5p orbitals, while the CBM consists of Sn 5p and I 5p antibonding orbitals; the contribution of Cs 5d orbitals are observed at ≈5 eV. All the CsSnI_3_ structures present a direct bandgap but at different points in the k‐space: R for the cubic, Z for the tetragonal, and Γ for the orthorhombic phase.^[^ ^67^ ^]^ A gradual increase in the bandgap was observed during the phase transition from cubic to orthorhombic structure upon increasing the tilting of the octahedra network. Huang and Lambrecht calculated the effective mass of CsSnI_3_ by fitting the parabolic dispersion curves of the energy band structures obtained from QSGW calculations and are shown in **Table** **5**.^[^ ^66^ ^]^ Near the VBM, the band is nondegenerate, and the band dispersion is isotropic, which led to a single value for the hole effective mass. The low value of 0.069 for the hole effective mass gave rise to the computation of a high hole mobility of ≈585 cm^2^ V^−1^ s^−1^.^[^ ^68^ ^]^ The conduction band maximum is a threefold degenerate state when SOC is not considered, while these states split into a singlet and a doublet when SOC is included and hence show an anisotropic effective mass.

**Table 5:** Calculated light (le) and heavy (he) effective masses of electrons along the [100] and [111] directions of the CsSnI_3_ cubic phase.^[^ ^66^ ^]^ There is only a value for the hole mass because the band is nondegenerate and the band dispersion is isotropic and parabolic near the VBM

*m* *_h_* ^*^	$m_{he}^{*}$ [100]	$m_{he}^{*}$ [111]	$m_{le}^{*}$ [100]	$m_{le}^{*}$ [111]
0.069	0.573	0.154	0.041	0.068

To summarize, the valence band maximum and conduction band minimum of tin perovskites are mainly composed of Sn 5s and 5p orbitals hybridized with I 5p orbitals, therefore the bandgap and effective masses are strongly correlated to the inorganic framework. Hence, in the tetragonal and the orthorhombic structures, when the B–X–B angles deviate from 180°, there is a consequent reduction of the orbital overlap and increases of the effective masses.

##### Optical Properties

**Figure 5:**
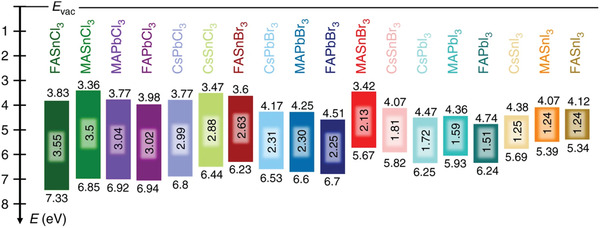
Schematic energy level diagram of the 18 most common metal halide perovskites. All the compounds are sorted in order of decreasing optical bandgap. Reproduced under the terms of the Creative Common Attribution 4.0 International license.^[^ ^38^ ^]^ Copyright 2019, Nature Research.

Due to the orbital composition of the band edges, the crystal structure directly influences the optical properties of perovskite compounds. In corner‐shared octahedral structures, the bandgap follows a straightforward relation with its symmetry, where an increase of the octahedral distortions results in a wider bandgap. Furthermore, increasing the size of the A‐site cation also widens the optical bandgap as the larger size introduces distortions in the structure and reduces the orbital overlap as discussed for the electronic bandgap previously. For example, at room temperature, the tetragonal crystal structure of MASnI_3_ has a lower bandgap than the orthorhombic structure of FASnI_3_.^[^ ^28^ ^]^ By contrast, increasing the size of the X‐site anion results in a smaller bandgap. For other nonperovskite structures the nature of the bandgap can change; for example, the hexagonal phase of MASnI_3_ has an indirect bandgap compared to the direct bandgap of cubic and tetragonal phases.^[^ ^69^ ^]^ **Figure** **5** shows the bandgaps of various Sn perovskite compositions, compared with the Pb‐based counterparts.^[^ ^38^ ^]^ For Sn‐based materials, only the bandgaps of CsSnI_3_, MASnI_3_, FASnI_3_, and FASnI_2_Br fall in the range of 1.2–1.6 eV, which makes them most suitable for single‐junction solar cells. The bandgap does not vary significantly by changing only the A‐site cations, but the variation is significant in case of the replacement of the X‐site halogens or B‐site ions (as quantified in Figure 5).^[^ ^66^ ^]^ By replacing I with Br in these compositions, the bandgap can be continuously tuned from 1.3 to 2.4 eV for optimal use in single‐junction and tandem solar cells. Nagane et al. demonstrated that by mixing Ge in MASn_1‐_ *_x_* Ge*_x_* I_3_, a monotonic shift in the absorption edge toward higher energy can be obtained.^[^ ^70^ ^]^

Understanding the photophysical properties of tin perovskites is paramount to further improve devices. In particular, absorption and photoluminescence (PL) spectra can provide important information on the material quality. Specifically, the PL peak position, the full width at half maximum (FWHM), and the photoluminescence quantum yield (PLQY) are crucial to determine the nature of the emission, the presence of disorder and defects, and the degree of electron–phonon coupling. As Sn‐based perovskites are prone to degradation, high care should be taken of the sample when the objective is determining its intrinsic optical properties. Even considering this difficulty, many studies contributed to the understanding of the photophysics of this class of materials.

**Figure 6:**
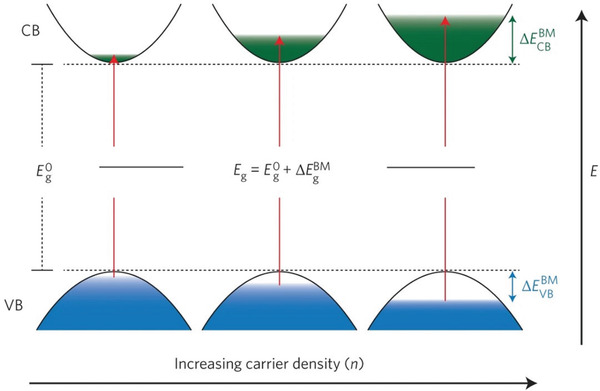
Schematic representation of the Burstein–Moss effect. Reproduced with permission.^[^ ^71^ ^]^ Copyright 2014, Springer Nature.

Ozaki et al. studied the optical properties of MASnI_3_ and FASnI_3_ compounds, highlighting the dependence of the optical properties on the quality of the films.^[^ ^72^ ^]^ The authors found that the absorption and PL are sensitive to the oxidation, which created a higher hole carrier concentration in the film. This doping of the film results in a blue shift of the absorption onset according to the Burstein–Moss effect, as shown in **Figure** **6**. By using SnF_2_ as an additive, the authors fabricated films with lower hole carrier density, observing a shift for the PL peaks of FASnI_3_ from 1.44 to 1.40 eV, with a smaller FWHM value of 110 meV versus the 160 meV obtained without additive. Milot et al. quantified the background hole doping densities and showed that the hole doping leads to a clear Burstein–Moss effect that increases the absorption onset of 300 meV for hole densities near 10^20^ cm^−3^.^[^ ^73^ ^]^ This effect also masks the intrinsic bandgap of FASnI_3_, which was reported to be 1.2 eV at 5 K and 1.35 eV at room temperature. A similar trend was observed by Kahmann et al., who investigated the photophysics of FASnI_3_ thin films of different film quality deposited via three distinct protocols.^[^ ^74^ ^]^ The authors showed that samples of low quality and high doping density have short PL decay and low PLQYs. The intrinsic properties of the PL peak position and FWHM were obscured in the low‐quality samples, whereas these properties could be investigated in high‐quality samples obtained with a 2D‐3D strategy. Intriguingly, the PL showed a long‐lived low‐energy state below 110 K only for the higher quality samples, suggesting presence of triplet states or a slight indirect bandgap as the origin. This indirect bandgap could be the result of a Rashba‐type splitting as calculated by DFT calculations, although disorder could also play a role.^[^ ^52^ ^]^ In addition, the luminescence intensity of high quality FASnI_3_ thin films and single crystals exhibited irregular trends upon cooling with a strong intensity increase down to 185 K, followed by a negative thermal quenching down to 125 K. The reason for this effect is not entirely clear but it is probably linked to the suppression of nonradiative recombination pathways.^[^ ^52^ ^]^

**Figure 7:**
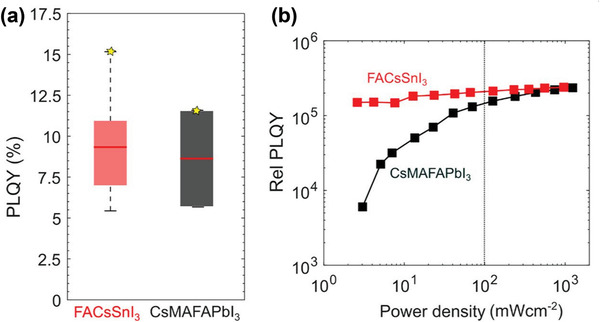
a) External PLQY and b) relative PLQY of FA_0.85_Cs_0.15_SnI_3_ and (FA_0.85_MA_0.15_)_0.95_Pb(I_0.85_Br_0.15_)_3_ thin films. Reproduced under the terms of the Creative Common Attribution 4.0 International license.^[^ ^75^ ^]^ Copyright 2021, ACS.

Poli et al. analyzed the PLQY of FA_0.85_Cs_0.15_SnI_3_ compared to (FA_0.85_MA_0.15_)_0.95_Pb(I_0.85_Br_0.15_)_3_ films.^[^ ^75^ ^]^ Both films showed similar PLQYs at excitation density close to 1 sun equivalent excitation. However, the relative PLQY of Pb‐based compounds increased as a function of the excitation power, while the PLQY of the Sn‐based films had a feeble slope (**Figure** **7**). These are typical trends in the presence of deep and shallow carrier trap states for Pb‐ and Sn‐based perovskite thin films, respectively. The predominant shallow defects in Sn‐based perovskites show once more its potential for high efficiency devices.

The next generation of photovoltaic devices could potentially exceed the Shockley–Queisser limit by extracting hot carriers, i.e., carriers with an excess energy in the band. However, to make the extraction possible a sufficiently slow relaxation of the hot carriers is a prerequisite.^[^ ^27^ ^]^ Fang et al. observed evidence of PL from hot carriers in FASnI_3_ with unreported long lifetime of a few nanoseconds.^[^ ^29^ ^]^ The asymmetry of the PL spectrum at the high‐energy edge is accompanied by an unusually large blue shift of the PL with increasing the excitation power (150 meV at 24 K and 75 meV at 293 K). This effect is caused by the slow hot‐carrier relaxation and subsequent filling of band edge states. Most importantly, the hot‐carrier PL was reported not only upon high‐density pulsed excitation but also with continuous wave excitation of concentrated 44 suns, which is essential to collect hot carriers from a practical point of view. Interestingly, Kahmann et al. have reported the hot carrier relaxation time to depend on the quality of the sample.^[^ ^74^ ^]^

## 3 “Hollow” Perovskites

### 3.1 Physical Properties

#### 3.1.1 Structural Properties

Recently, a new class of 3D tin halide perovskites, referred to as “hollow” perovskites, were reported by the Kanatzidis’ group. This type of perovskites is derived from the 3D ABX_3_ compounds by replacing tin halide octahedra with small organic cations such as ethylenediammonium (*en*), propylenediammonium (PN) or trimethylenediammonium (TN) in the crystal structure without altering the 3D dimensionality.^[^ ^76^, ^77^, ^78^ ^]^ These cations can replace tin halide octahedra, hence resulting in hollow vacancies and increased structural disorder in the 3D framework.^[^ ^79^ ^]^ Ke et al. first demonstrated the benefit of the hollow structure in Sn‐based perovskites and demonstrated that these hollow structures can improve the device efficiency and stability.^[^ ^76^, ^77^ ^]^

**Figure 8:**
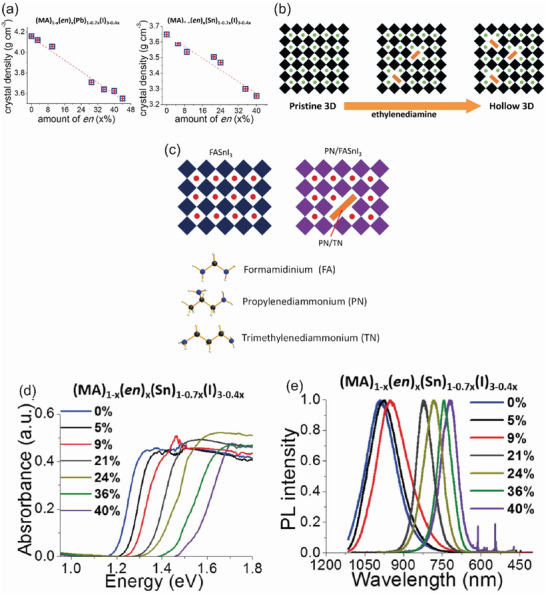
a) Correlation of the percentage of *en* on the measured crystal density in (MA)_1‐_ *_x_* (*en*)*_x_* (Sn)_1‐0.7_ *_x_* (I)_3‐0.4_ *_x_*. b) Schematic illustration of the structure of the 3D pristine MASnI_3_ perovskite and its transformation from a dense structure to a hollow 3D structure with increasing amount of *en*. Black cubes: [SnI_6_]^4−^ octahedra; green spheres: MA cations; orange rectangles: *en* cations. c) Schematic illustration of the structure of the 3D pristine FASnI_3_ perovskite, 3D hollow FASnI_3_ with PN or TN and molecular structures of FA, PN, and TN. d) Absorbance and e) PL spectra of the compound (MA)_1‐_ *_x_* (*en*)*_x_* (Sn)_1‐0.7_ *_x_* (I)_3‐0.4_ *_x_* with increasing *en* concentration. (a,b,d,e) Reproduced with permission.^[^ ^79^ ^]^ Copyright 2018, American Chemical Society. (c) Reproduced with permission.^[^ ^78^ ^]^ Copyright 2018, American Chemical Society.

We start by discussing the inclusion of *en* reported by the pioneering work of Spanopoulos et al. The authors estimated the *en* radius to be 3.33 Å according to the model proposed by Kieslich et al.^[^ ^79^, ^80^ ^]^ Considering this value, the authors calculated a tolerance factor of 1.21 which largely deviates from the range of 0.8 < *t* < 1.0 required to form the 3D perovskite structure. This large discrepancy was partially alleviated by mixing *en* with MA in (MA)_0.6_(*en*)_0.4_(Sn)_0.72_(I)_2.84_, although the tolerance factor of 1.08 also lies outside the optimum range for 3D perovskites formation. Therefore, the structure to accommodate the large *en* molecules and maintain its 3D dimensionality had to replace some A, B, and X ion sites. Detecting the crystallographic site of *en* is difficult because of the disorder and mixed occupancy with MA cations. However, the density of hollow perovskites decreases systematically as a function of *en* concentration, in agreement with the hollow nature of these perovskites (**Figure** **8**a). This high loss cannot be explained by a simple substitution of two MA molecules with one *en* cation because the mass difference in this case would be negligible. Therefore, this result highlights that the *en* cation creates octahedral vacancies. A schematic representation of this hollow structure is shown in Figure 8b. Based on these results, Sponopoulos et al. proposed the general composition of hollow MASnI_3_ using *en* ^2+^ cations.^[^ ^79^ ^]^ There are two possibilities to take in account: all Sn^2+^ are displaced by *en* ^2+^ in a stoichiometric manner, or, *en* ^2+^ displaces two MA^+^ molecules and a neutral SnI_2_ unit. For the first case, the authors observed that it is unlikely for the *en* cation to replace only Sn^2+^ in the structure due to its larger size. This is only possible if there is an additional removal of an A^+^ cation or an I^−^ ion. For the second case, *en* cations can replace a SnI_2_ and two MAI moieties in a statistical manner in any combination that can afford a charge balanced compound, exhibiting a good agreement between the calculated and the experimental crystal densities. Hence, this second case is the most likely explanation for the formation of the hollow MASnI_3_.

Despite the larger sizes of TN and PN cations compared to *en*, Ke et al. incorporated them into the 3D perovskite structure, as shown in Figure 8c.^[^ ^78^ ^]^ All the crystals showed diffraction peaks characteristic of the 3D FASnI_3_ perovskite structure. The unit cell parameters of {PN}FASnI_3_ and {TN}FASnI_3_ suggested a small expansion of the unit cell size with respect to the 3D system after the incorporation of PN and TN cations, although their NMR results suggested that not all PN or TN in the precursor solution were incorporated in the final crystal, slightly favoring TN over PN.

#### 3.1.2 Electronic and Optical Properties

Although hollow perovskites have a 3D structure, the optical and electronic properties of the hollow structures are different from the standard 3D compounds. Sponopoulos et al. observed that the bandgap was increased by ≈40% with respect to the pristine perovskite at an *en* concentration equal to 0.4 m. Importantly, the direct nature of the bandgap was conserved (Figure 8d).^[^ ^79^ ^]^ The widening of the bandgap is attributed to the disruption of the crystal lattice rather than to a thermal expansion mechanism of the structure.^[^ ^81^ ^]^ Due to the presence of Sn^2+^ and I^−^ vacancies, the orbital overlap among the remaining Sn/I is reduced and this causes the narrowing of the VB and CB bandwidth, leading to the experimentally observed increase of the bandgap.^[^ ^76^ ^]^ As expected by the widening of the bandgap, the photoluminescence at room temperature shows a blue shift with increasing fraction of *en* (Figure 8e).^[^ ^79^ ^]^ Furthermore, the Stokes shift increases, reaching a maximum of 0.22 eV for the (MA)_0.6_(en)_0.4_(Sn)_0.72_(I)_2.84_ compound. The decrease of the photoluminescence intensity with increasing *en* concentration is linked to the effective disconnection of the perovskite structure. The disorder in the structure is expected to determine the presence of charged point defects in large concentration, which may act as nonradiative recombination centers.

Similarly to MA compounds, (FA)_1‐x_(*en*)*_x_* (Sn)_1‐0.7_ *_x_* (I)_3‐0.4_ *_x_* materials exhibited a large blue shift of the absorption edge and PL peak with increased *en* concentration.^[^ ^76^ ^]^ This trend suggested an opening of the bandgap from ≈1.3 eV for the black FASnI_3_, to ≈1.9 eV for the 5:1 molar ratio of red (FA)_1‐_ *_x_* (*en*)*_x_* (Sn)_1‐0.7_ *_x_* (I)_3‐0.4_ *_x_* molar ratio. DFT calculations again showed a reduction of the electronic bandwidths, with the VB showing a larger decrease than the CB. The reduction of the VBM increases the stability of the perovskite because the absolute work function is shifted to lower energy, becoming less prone to oxidation.^[^ ^76^ ^]^

TN cations influence the optical properties of the 3D perovskites as well. Ke et al. found that the absorption onset of the 10% and 40% TN films is slightly blue‐shifted by ≈20 and ≈30 nm, respectively.^[^ ^78^ ^]^ On the contrary, the absorption onset of the 10% and 40% of PN films is the same as the onset of the pristine FASnI_3_ film. The smaller bandgap of TN compared to *en* hollow perovskites suggested that also octahedral vacancies form in films cast with TN albeit in a smaller number.^[^ ^78^ ^]^

## 4 Low‐Dimensional Layered Perovskites

Continuous research efforts pushed the PCE of tin perovskite solar cells above 14%, making them one of the most promising “lead‐free” materials. However, despite their increasing PCEs, tin‐based devices are prone to degradation. In the search for higher stability against high temperatures and moisture, low‐dimensional layered perovskites gained great attention both in lead‐ and tin‐based perovskites. These layered structures consist of alternating inorganic and organic layers, which naturally form 2D quantum wells, 1D quantum rods, or 0D quantum dots, depending on their chemical composition. The composition of these layered perovskites is not limited to the size of the cation on the A position, as it is for the 3D perovskite structure, therefore offering the opportunity for endless new compositions and structures. Hence, an exciting playground is established for chemists and physicist to investigate their structural and optical properties.

### 4.1 Physical Properties

#### 4.1.1 Structural Properties

**Figure 9:**
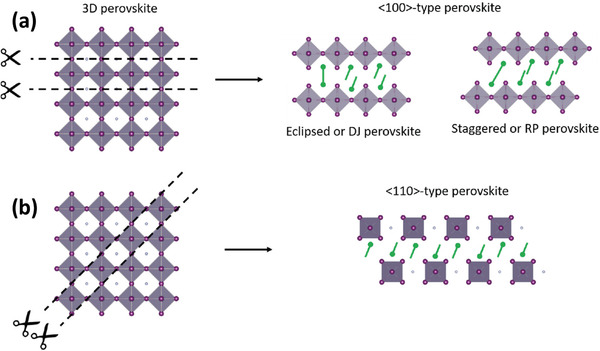
Cutting the 3D parent structure along the a) 〈100〉 and b) 〈110〉 planes results in lower dimensional perovskites: the 2D quantum wells and 1D quantum rods, respectively. The 〈100〉‐type perovskites are commonly categorized into the Dion–Jacobson (DJ) or Ruddlesden–Popper (RP) phase perovskites.

As discussed in the previous chapter, the ABX_3_ perovskite structure has strict requirements on ionic size of the A‐site cation to ensure that the 3D structure is obtained. When the size restrictions of the A‐site cation are lifted, i.e., a larger organic cation is used, the 3D perovskite structure breaks into lower dimensional structures. 2D layered perovskites can be envisioned by slicing the parent 3D structure along specific crystallographic directions. The layered perovskites are then formed by introducing either monovalent (A′) or divalent (A″) organic cations in between the perovskite‐like layers; for example, **Figure** **9** illustrates a schematic representation of two types of layered perovskites, the 〈100〉 type and the 〈110〉 type. The 〈100〉 type is the most frequently encountered low‐dimensional perovskite, while only a handful of compounds are known to result in the 〈110〉‐type perovskite. A large number of distinct structures have already been reported, and the interested reader is referred to the excellent open‐access database published by Marchenko et al. to access the structural parameters of the systems discussed below.^[^ ^82^ ^]^

**Figure 10:**
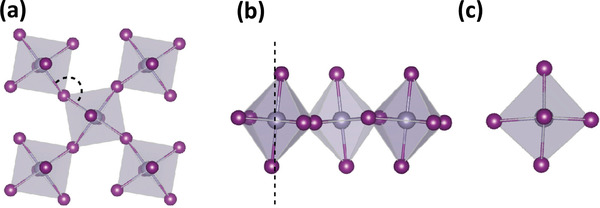
Layered perovskites can show a) an in‐plane distortion, b) an out‐of‐plane distortion, and c) an octahedral distortion. The in‐plane distortion reduces the Sn–X–Sn angle from the ideal 180°. The out‐of‐plane distortion introduces a tilting of the octahedra, and the octahedral distortion accounts for the deviation of the octahedral geometry of the inorganic cages.

As opposed to their 3D counterpart, layered perovskites often deviate from the ideal perovskite connectivity, where the organic cations introduce distortions of the layers. We define three types of distortions, as shown in **Figure** **10**. The in‐plane distortion is observed when the angle between octahedra (the Sn–X–Sn angle) is smaller than the ideal 180°, as shown in Figure 10a. The out‐of‐plane distortion is shown in Figure 10b and can be described by the out‐of‐plane tilting of the octahedra. The individual octahedra can also be distorted, commonly resulting in a variation of the Sn–X bond lengths or X–Sn–X bond angles, as shown in Figure 10c. In the following section, the distortion of these inorganic layers based on the choice of the organic cation is discussed.

##### The 〈100〉‐Type Perovskite

By slicing the cubic perovskite structure along the 〈100〉 plane, the 〈100〉‐type perovskites are obtained. The inorganic sheets are commonly separated by large mono (A′) or diammonium (A″) organic cations, resulting in a layered structure. These layered perovskites are denoted by the chemical formula (A′)_2_(A*_n_*_‐1_)Sn*_n_* X_3_ *_n_*_+1_ or (A″)(A*_n_*_‐1_)Sn*_n_* X_3_ *_n_*_+1_, where *n* represents the number of inorganic layers sandwiched between the large organic cations. The *n* = 1 of the 〈100〉‐type perovskite is regarded as ideal 2D inorganic sheets. By increasing the thickness of the inorganic layers (*n* > 1), the so‐called “quasi‐2D” structure is obtained. In this section, we will focus on the *n* = 1 case first and will discuss the quasi‐2D structures thereafter.

In the case of monoammonium cations, the inorganic layers are held together through weak van der Waals interactions between the organic cations, while the hydrogen bonding of the ammonium group to the halides anchors the organic cations to the inorganic layers. Examples of two frequently used monoammonium cations are phenethylammonium (PEA) and butylammonium (BA).^[^ ^83^, ^84^ ^]^ Diammonium cations fix the inorganic layers position through the hydrogen bonding of the ammonium groups on two adjacent inorganic layers, increasing the strength of the interlayer bonding compared to monoammonium cations. The two adjacent inorganic layers can be stacked with an offset of the apical iodines in the stacking direction (staggered configuration) or without any offset (eclipsed configuration), as shown in Figure 9a. These two configurations are commonly denoted as the Ruddlesden–Popper (RP) phase and the Dion–Jacobson (DJ) phase perovskites, respectively.

The RP phase perovskites were named after Ruddlesden and Popper who confirmed that the structure of Sr_2_TiO_4_ was of the K_2_NiF_4_‐type structure, as proposed earlier by Balz and Plieth.^[^ ^85^ ^]^ Strictly speaking, this structure is characterized by a tetragonal unit cell where two adjacent perovskite layers are offset by (1/2,1/2,1/2). DJ phase perovskites inherited their name based on two separate reports of Dion et al. and Jacobson et al. on tetragonal A′Ca_2_Nb_3_O_10_ (A′ = Rb^+^, K^+^) crystal structures.^[^ ^86^, ^87^ ^]^ In contrast to RP phase perovskites, DJ phase perovskites can have two motifs: one with an offset of the perovskite layers of (1/2,0,0) or one with no offset (0,0,0), depending on the used alkali metal. Nowadays, layered perovskites using two large monoammonium cations are often used as synonyms for RP phase perovskites, while perovskites using one large diammonium cation are directly associated with DJ phase perovskites.^[^ ^88^, ^89^ ^]^ Especially in the solar cell community the terms RP and DJ are frequently used to indicate this distinction. The organic cations in layered metal halide perovskites offer a great variety in stacking motifs, and a wide range of offsets have been observed.^[^ ^89^ ^]^ In addition, it has been advocated that the divalent organic cations are not comparable to the metals used in oxide perovskites, hence one should not use the term DJ phase perovskites to describe the 〈100〉‐type perovskites.^[^ ^90^ ^]^ As there is no clear‐cut consensus on the correct naming, so we choose to discuss the structures below according to the cut of the crystallographic plane.

**Figure 11:**
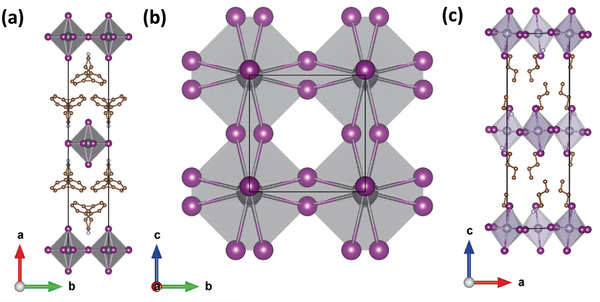
(PEA)_2_SnI_4_ crystal structure projected along a) the *c*‐axis and b) the *a*‐axis. Both the organic cations and equatorial iodines are disordered over two positions. (BA)_2_SnI_4_ crystal structure projected along c) the *b*‐axis. The black lines indicate the unit cell. Structures have been reproduced using crystallographic parameters.^[^ ^91^, ^92^ ^]^

Many of the pioneering works of tin‐based 〈100〉‐type perovskites date back to the 1990s.^[^ ^93^, ^94^ ^]^ Papavassiliou et al. were the first to synthesize (PEA)_2_SnI_4_ and (PEA)_2_SnBr_4_ in pure crystalline form.^[^ ^95^ ^]^ The authors reported that (PEA)_2_SnI_4_ is isostructural with its Pb counterpart based on powder XRD results, which was confirmed in a subsequent study.^[^ ^91^ ^]^ **Figure** **11**a,b shows the crystal structure of (PEA)_2_SnI_4_ along two different projections. Besides the layered nature of the crystal, the organic cations are disordered over two positions as well as the equatorial iodines. This is a common observation in many layered perovskites and might be related to disorder and unresolved superstructures.^[^ ^91^, ^96^, ^97^ ^]^ The inorganic layers of (PEA)_2_SnI_4_ consist of corner shared octahedral cages, which are distorted with respect to the ideal cubic perovskite structure. An in‐plane rotation is observed, reducing the Sn–I–Sn angle from the ideal 180° to 156.5°. This distortion accommodates for the relatively large charge density of the terminal ammonium group and can vary with the use of the organic cation.^[^ ^98^ ^]^ Papavassiliou et al. also reported the first use of an alkylammonium chain with ten carbon atoms in Sn‐based perovskite, although polycrystalline materials were obtained inhibiting single crystal analysis techniques.^[^ ^99^ ^]^ Mitzi et al. were able to synthesize phase pure single crystals of a series of layered perovskites using butylammonium, the crystal structure of (BA)_2_SnI_4_ is shown in Figure 11c.^[^ ^83^, ^92^ ^]^ The basal plane was enlarged by approximately a square root of 2 compared to PEA‐based structure due to distortion of the inorganic layers combined with ordering of the organic cations. (BA)_2_SnI_4_ has a larger out‐of‐plane tilting but a smaller in‐plane distortion compared to (PEA)_2_SnI_4_.

Due to the specific distortion introduced by each organic cation, the organic cations were said to template the inorganic layers.^[^ ^98^ ^]^ Interestingly, the reverse is also true. For example, the PEA cation adopted the gauche conformation in tin‐based layered perovskites, whilst an anticonformation was observed for copper‐based 〈100〉‐type perovskites.^[^ ^100^ ^]^

The conformation of the organic cation was further investigated by synthesizing a series of PEA derivatives, namely (2‐XPEA)_2_SnI_4_, where X is F, Cl, and Br and were substituted on the orthoposition of the phenyl ring.^[^ ^101^ ^]^ For F and Cl substitution, the commonly seen gauche conformation was observed, similar to PEA. However, Br substitution changed the conformation to anti, as the steric interaction of the substituted atom prevents the gauche conformation. The effect of steric hindrance also has a pronounced effect on the Sn–I–Sn angle. The angle decreased significantly from 154.8° for 2‐ClPEA to 148.7° for 2‐BrPEA, introducing a larger in‐plane distortion of the inorganic layers.

**Figure 12:**
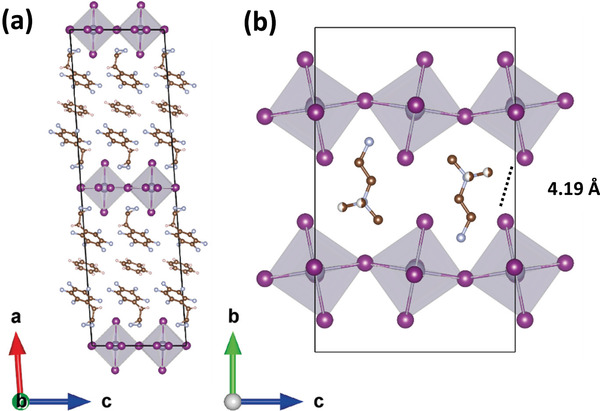
a) Intercalated benzene rings are stabilized between 2,3,4,5,6‐pentafluorophenethylammonium cations. b) Trimethylammonioethylammonium dications distorts the inorganic layer such that the apical iodines point outward to accommodate the bulky quaternary ammonium, while the iodines close to the slender primary ammonium group point inward. Structures have been reproduced using crystallographic parameters.^[^ ^102^, ^103^ ^]^

Besides the size of the substituted atom, the substitution position also affects the crystal structure.^[^ ^97^ ^]^ The position of the fluorine substitution on the phenyl ring determines an increase of the average in‐plane Sn–I–Sn angle from 153.3° to 154.2° and 156.4° for the ortho, meta, and para substitution, respectively. The almost identical Sn–I–Sn angle for the para‐substituted fluorine atom compared to the nonsubstituted organic cation suggests the little steric hindrance of the fluorine atom. A similar trend was obtained for the Sn—I bond distances, where slightly larger bond lengths were observed for the ortho and meta position to accommodate the size of the fluorine atom. In the early 2000s, Mitzi et al. showed that more complex structures could be obtained by intercalating hexafluorobenzene in between PEA cations or benzene in between 2,3,4,5,6‐pentafluorophenethylammonium (5FPEA), as illustrated in **Figure** **12**a.^[^ ^102^ ^]^ The authors reported that the fluoraryl–aryl interactions were essential for obtaining the intercalated structures, as they were unable to stabilize benzene in between PEA cations. Intriguingly, the intercalated molecules could be removed and added reversibly. A deintercalation endothermic reaction was observed by heating thin films above 143 °C, while putting the films in a solution of the intercalating molecules restored the intercalated structure.

Thus far, we have considered only primary ammonium groups interacting with the inorganic lattice. Figure 12b shows the structure using trimethylammonioethylammonium as the large organic cation.^[^ ^103^ ^]^ This divalent cation has a primary ammonium group at one end and a quaternary ammonium group on the other end, which interact each with the two adjacent inorganic layers. The relative short organic cation ensured a close proximity of iodine–iodine of 4.19 Å. The bulky quaternary cation induced an outward tilting of the apical iodines, while for the narrow primary cation the iodines point inward. Surprisingly, this structure results in larger Sn–I–Sn angles of 162.3° and 171.5° compared to most primary monoammonium cations having angles between 150° and 160°.^[^ ^98^ ^]^ The distortion of the inorganic layers could be further reduced by using 2,2′‐biimidazolium cations, reaching an Sn–I–Sn angle of 171.83°.^[^ ^104^ ^]^ While the angle between the octahedral cages was close to the ideal angle of 180°, the individual octahedral cages were greatly distorted with Sn—I bond lengths varying between 2.93 and 3.55 Å. This example illustrates that the three types of distortion presented above are often interconnected.

An even greater octahedral distortion was achieved with 3‐iodopyridinium (iPy), where two bond lengths were significantly shorter (2.9 Å) compared to the two longer bond lengths of 3.47 and 3.66 Å.^[^ ^105^ ^]^ Although the octahedral distortion has generally been associated with the stereo‐activity of the electron lone pair, increasing from Pb to Sn and Ge, it is not clear a priori what type of distortion will be present for distinct organic cations.^[^ ^92^, ^104^ ^]^

Already in one of the first studies it was noted that layered perovskites were affected upon exposure to ambient conditions.^[^ ^84^ ^]^ These colorful crystals can degrade quickly into black oxidized species within minutes of exposure to air.^[^ ^106^ ^]^ Layered perovskites are an excellent class of materials to engineer many properties including their stability, as the organic cations can be designed to prevent fast oxidation of the crystals for example, when they are hydrophobic. Recently, it was shown that 2‐(4‐(3fluoro)stilbenyl)ethanammonium (FSA) tin iodide remained stable at ambient conditions (60% relative humidity) for 11 days, while (PEA)_2_SnI_4_ showed signs of degradation after 1 day.^[^ ^107^ ^]^ Notably, (FSA)_2_SnI_4_ crystals were even stable in water for up to 60 min. The authors of this work, proposed that the increased stability was achieved due to increased π–π and the interaction of fluorine with water.

In the discussion above, we focused on layered perovskites using iodine as opposed to bromine. Reports on the crystal structures of bromine are scarce; below we briefly discuss these reports. In general, substituting iodine with bromine results in small structural changes in 〈100〉‐type perovskites. For example, (PEA)_2_SnBr_4_ showed a similar layered structure as the iodine counterpart.^[^ ^108^ ^]^ This similarity was utilized in a series of thin films, where the bromine was gradually interchanged with iodine.^[^ ^109^ ^]^ Bromine‐based compounds have also similar flexibility in the choice of organic cations. Recently, a study reported a set of layered perovskites using alkyl ammonium chains with 8, 12, and 18 carbons.^[^ ^110^ ^]^ As expected, increasing the length of the alkyl chain resulted in a larger distance between the inorganic layers. Noteworthy, the authors observed a decreased crystallinity with increasing alkyl lengths, and crystals with less than eight carbons in the alkyl chain could not be obtained.

An especially interesting property of bromine‐based perovskites is the higher stability of these compounds. Papavassiliou et al. mentioned an early work that bromine compounds are more stable in air than the iodine variants.^[^ ^95^ ^]^ Recently, octylammonium tin bromide crystals showed excellent stability in air for over 240 days.^[^ ^111^ ^]^ Additionally, it was shown that these crystals could be used as scintillators for X‐ray imaging applications, a feature encountered among various tin–bromide compounds.^[^ ^112^, ^113^ ^]^ Furthermore, (PEA)_2_SnBr_4_ has even been used in aqueous environment for the production of hydrogen.^[^ ^114^ ^]^ Fully inorganic layered structures have inherent higher stability, however the choice of cations is much more limited, imposing a challenge on the synthesis of these crystals. We are aware of only one report on such a Sn‐based perovskite, that is Cs_2_SnCl_2_I_2_.^[^ ^115^ ^]^

##### The 〈110〉‐Type Perovskite

**Figure 13:**
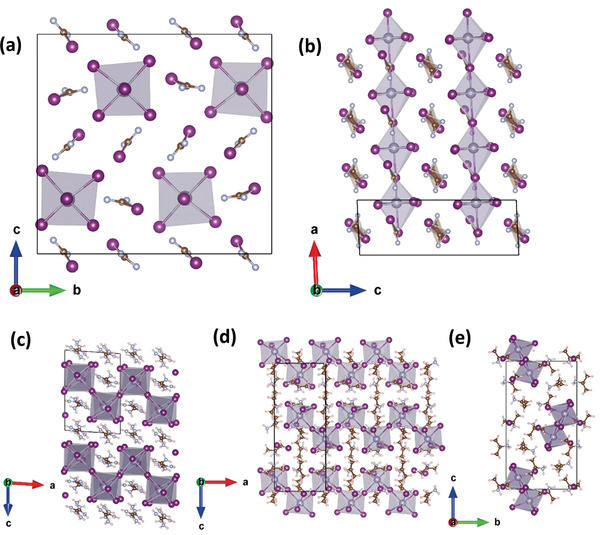
(IFA)_3_SnI_5_ projected along the a) *a‐*axis and b) *b‐*axis. This *n* = 1 〈110〉‐type perovskite forms 1D inorganic chains. Structures have been reproduced using crystallographic parameters.^[^ ^116^ ^]^ (GA)_2_SnI_4_ projected along the c) *b*‐axis, and (IPA)_3_SnI_5_ projected along the d) *b*‐axis and e) *a*‐axis. Structures have been reproduced using crystallographic parameters.^[^ ^106^ ^]^

Evidently, the 〈110〉‐type perovskite is obtained when slicing the parent 3D structure along the 〈110〉 direction. These structures are denoted by the chemical formula (A′)_2_(A)*_n_* Sn*_n_* X_3_ *_n_*_+2_. The 〈110〉‐type perovskite are not as frequently synthesized as the 〈100〉‐type perovskite due to the difficulty of predicting the 〈110〉‐type structure. **Figure** **13** shows the structure of iodoformamidinium tin iodide (IFA)_3_SnI_5_, which can be considered as the *n* = 1 case.^[^ ^116^ ^]^ The key difference in this 〈110〉‐type structure compared to the 〈100〉‐type is that along the *b* axis the octahedra are separated by the iodoformamidinium cations. Therefore, the octahedra form 1D chains along the *a*‐axis, see Figure 13b. The Sn–I–Sn angle of these chains is 177.3°, hence slightly corrugated chains are obtained.^[^ ^116^ ^]^ In addition to the corrugation of the chains, Figure 13a shows that the octahedra are distorted with the longer and shorter bonds opposing each other. The three longer bonds suggested that the lone pair is localized in this direction, where a larger effect was seen in Sn compounds compared to Pb variants. More recently, it has been shown that this distortion could be reduced by substituting the iodoformamidinium in the *ab* plane with formamidinium, forming a mixed organic cation composition.^[^ ^117^ ^]^ By contrast, a larger octahedral distortion was obtained in the compound [CH_3_SC(NH_2_)_2_]_3_SnI_5_ with one of the Sn—I bonds being exceptionally large, namely, 4.04 Å.^[^ ^118^ ^]^

So far, the *n* = 1 member has only been stabilized by a few organic cations, while for the *n* = 2 members several examples exist.^[^ ^106^, ^119^, ^120^ ^]^ The *n* = 2 member of this family is formed by connecting the 1D chains in a zigzag formation, as shown in Figure 13c for guanidinium tin iodide (GA)_2_SnI_4_.^[^ ^106^ ^]^ This chemical formula can be confusing as it resembles the chemical formula for the *n* = 1 case in 〈100〉‐type perovskites. This confusion stems from the reduction of the chemical formula (GA)_2_(GA)_2_Sn_2_I_8_ to (GA)_2_SnI_4_. However, it is evident that these two types of low‐dimensional perovskite are very different due to the zigzag motif of the 〈110〉‐type. This difference is further corroborated by the electronic properties, which will be discussed in the next section. Recently, a study showed that half of the interlayer of guanidinium could be replaced by 1‐methylimidazolium (Mi), resulting in the chemical formula (GA)_1.5_(Mi)_0.5_SnI_4_.^[^ ^119^ ^]^ The authors proposed that the larger 1‐methylimidazolium could not fit into the intralayer guanidinium sites, therefore the interlayer crystallographic sites remain. Indeed, Mi replaced only half of the GA sites, showing that further compositional variation is possible.

##### Exotic Layered Structures

Besides the general 〈100〉 and 〈110〉 families discussed above; a large number of layered compounds exist that are rarely observed but show interesting structural features. The following two studies show features also found in the general class of 〈110〉‐type perovskites, therefore they are discussed first.

**Figure 14:**
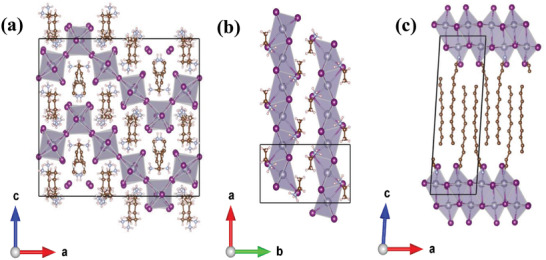
a) 〈330〉‐type perovskite with pentanediammonium cations, b) perovskitoids structure showing exclusively face‐sharing octahedra using ethylammonium cations, and c) n‐dodecylammonium templating a double layered inorganic layer showing both corner‐ and edge‐sharing connectivity. Structures have been reproduced using crystallographic parameters.^[^ ^106^, ^123^, ^124^ ^]^

Interchanging GA with isopropylammonium (IPA) resulted in a new structure (IPA)_3_SnI_5_, which is shown in Figure 13d,e.^[^ ^106^ ^]^ This structure consists of a 1D zigzag chain as opposed to the zigzag sheet in Figure 13c and can be envisioned as a single layer of the *n* = 2 structure, i.e., cutting the layered structure perpendicular to the *b* axis. 1D zigzag chains are also observed in (GA)_2_SnBr_4_ and (GA)_2_SnCl_4_, however, square pyramidal tin halide cages are formed instead of octahedral tin halide cages.^[^ ^121^, ^122^ ^]^ These structures are highly different compared to the iodine variant and illustrate the profound impact of the halides on the structure. An exotic 〈330〉‐type structure could be obtained by using pentanediammonium as the organic cation.^[^ ^123^ ^]^ This structure links the corner shared octahedra by alternating two *trans* conformations of the octahedra with one *cis*, as shown in **Figure** **14**a. Intriguingly, this structure could only be obtained if methylammonium was included in the synthesis, while it is absent in the crystal structure. Synthesis without methylammonium resulted in a 〈100〉‐type layered perovskites as one would intuitively expect from an alkyldiammonium cation.

Thus far, we have only considered octahedra that are linked via corner sharing, while with edge and face sharing vast new families of compounds can be created. We note that as one deviates from purely corner‐shared octahedra, it is more appropriate to denote these as perovskite‐related structures. Stoumpos et al. synthesized two new families of metal halide perovskite‐related structures, which they named “perovskitoids” and “hexagonal polytypes.”^[^ ^106^ ^]^ The former consists of exclusively face sharing octahedra and form 1D chains as shown in Figure 14b, while the latter can be considered as the intermediate regime between perovskitoids and fully corner‐shared octahedra. Hence, the family of hexagonal polytypes consists of alternating corner‐ and face‐sharing octahedra. Interestingly, these hexagonal polytypes create large cavities that can hold up to three ethylammonium cations.^[^ ^106^ ^]^ Xu and Mitzi reported a perovskite‐related structure which consisted of both corner‐ and edge‐sharing octahedra as shown in Figure 14c.^[^ ^124^ ^]^ This structure consisted of a double layer of octahedra separated by *n*‐dodecylammonium (DoA) cations and is described by the chemical formula DoASnI_3_. This structure is unusual as one would expect a 〈100〉‐type layered structure using long alkylammonium chains.^[^ ^124^ ^]^

The fashion in which the inorganic octahedra are connected is highly tunable and results in fascinating structures as described above. Recently, another family of compounds consisting of completely isolated octahedra, which are appropriately termed 0D compounds, appeared in the literature.^[^ ^108^, ^115^, ^125^, ^126^, ^127^, ^128^, ^129^ ^]^ Zhou et al. first reported this type of structure for *N*,*N*′‐dimethylethylenediammonium tin bromide.^[^ ^130^ ^]^ The isolated tin bromide octahedra are molecular‐like 0D structures, which are different from spatially confined quantum dots. These materials are highly promising for light emitting applications, as high photoluminescence quantum yields (PLQYs) are expected. Indeed, the high PLQYs were reported in a follow‐up study, with values of ≈95% and 75% for bromine and iodine crystals, respectively.^[^ ^127^ ^]^ Alongside high PLQYs, these materials showed excellent stability under illumination; making them relevant for commercial lighting applications. We refer to Section 4.1.2 for further discussion on their optical properties.

A recent study by Xu et al. showed that a 0D perovskite structure could be obtained by intercalating dichloromethane in the (PEA)_2_SnI_4_ structure.^[^ ^108^ ^]^ Dichloromethane greatly impacted the dimensionality of the system, as it changed from 2D to 0D, which is opposite to the fluoroaryl–aryl interactions described previously. Contrary to the study by Xu et al., Zhou et al. did not observe the intercalation of dichloromethane in their structure, although it is possible that it plays an important role in stabilizing the 0D structure.^[^ ^130^ ^]^ Methanol can also be used to synthesize these 0D structures, resulting in distorted octahedra with two shortened and two extended Sn—Br bonds in the equatorial plane.^[^ ^129^ ^]^ 0D structures can also be obtained using only inorganic components, where Cs or Rb replaces the organic cation.^[^ ^115^, ^131^ ^]^ This is expected to further improve their thermal stability and has shown to be promising for thermometry applications.^[^ ^128^ ^]^

It is clear from the discussion above that many different Sn‐based layered structures have been synthesized, and with an increased interest and effort many new structures are expected to be reported in the future. One interesting aspect of Sn‐based layered perovskites is that various polymorphs can be obtained by varying the synthesis procedure, which has two important consequences: first, detailed and accurate descriptions of synthetic procedures are of particular importance; second, verification of the crystal structures by single crystal XRD methods are always necessary. The need for verification is also highlighted by the distinct crystal structures reported for (PEA)_2_PbI_4_, namely, *C2/m*, *P2_1_/c*, and *P‐1*, suggesting that slight differences in structures could be also obtained in Sn‐based compounds.^[^ ^96^, ^132^, ^133^ ^]^ Accurate structure determination is essential to understand the electronic and optical properties of layered perovskites, which will be discussed in detail in the following sections.

##### Quasi‐2D Perovskites

3D perovskites and the *n* = 1 of the 〈100〉‐type perovskites can be considered as the two extremes of a whole series of low‐dimensional perovskites, where the former incorporates only small organic cations, and the latter consists entirely of large organic cations. A mixture of small and large cations could be used to synthesize layered perovskites with varying thickness of the inorganic sheets, following the chemical formula (A′)_2_(A*_n_*_‐1_)Sn*_n_* X_3_ *_n_*_+1_. Structures with values of *n* between 2 and 5 are often referred to as “quasi‐2D.” This family of materials is highly relevant for device applications, as they are intended to combine the excellent optoelectronic properties of 3D perovskites with the improved stability of layered perovskites.

In 1994, Mitzi et al. reported a series of quasi‐2D structures (*n* from 2 to 5) using butylammonium and methylammonium cations.^[^ ^83^ ^]^ For the *n* = 5 case, a 90% pure crystalline phase was observed with the remaining fraction being *n* = 4, indicating that phase‐pure high‐*n* value crystalline phases are difficult to obtain. This was underlined by a recent report stating that accurate weighing of the precursors is essential.^[^ ^134^ ^]^ While for Pb‐based compounds structures with *n* = 7 have been obtained, for Sn‐based perovskites the highest pure phase obtained is *n* = 5.^[^ ^135^ ^]^ In these multilayered compounds one needs to consider that the outer octahedral layers, i.e., near the large organic cations, are structurally different than the inner layers.^[^ ^83^ ^]^ For the *n* = 3 case, the inner layer shows no octahedral distortion, while the apical Sn–I bonds of the outer layer are distorted with bond lengths of 3.00 and 3.28 Å. Recently, the structures using butyldiammonium were reported with *n* ranging from 1 to 3, where the relatively short organic cation imposed a shorter distance between the inorganic layers than expected from the van der Waals distance.^[^ ^136^ ^]^ Synthesis of higher *n*‐value of the 〈110〉‐type perovskite was achieved by combining iodoformamidinium and methylammonium cations.^[^ ^137^ ^]^ Here, the iodoformamidinium is located at the interlayer crystallographic sites, while the methylammonium is positioned at the intralayer sites. Increasing *n* from 2 to 4 resulted in a decrease of the monoclinic angle from 92.96° to 91.83°, confirming that higher *n*‐values will approach the higher symmetry of 3D perovskites.

The number of reports on structural characterization of quasi‐2D perovskites has been limited to a few cases. This can be ascribed to the difficulty of obtaining pure crystalline phases of quasi‐2D perovskites in single crystals. Although difficult, the study of these quasi‐2D perovskites is very important, as many device applications lately used these quasi‐2D perovskites. With new studies on phase‐pure quasi‐2D single crystals, one could identify the intrinsic properties of these intermediate structures. In turn, this could advance our understanding of polycrystalline thin films by differentiating intrinsic effects from the one determined by grain boundaries and mix phases.

#### 4.1.2 Electronic and Optical Properties

Layered perovskites consist of inorganic structures of decreased dimensionality; hence the electronic and optical properties of these materials are greatly affected. Initially it was thought that the Sn‐based layered perovskites were semiconducting for low *n*‐values with increasing metallic behavior for larger *n*‐values.^[^ ^83^, ^137^ ^]^ This trend was correlated with the reduced distortion of the lattice for larger *n* values, approaching the ideal cubic perovskite structure.^[^ ^92^ ^]^ In 2007, Takahashi et al. proposed that spontaneous p‐type doping caused the high conductivity of the layered perovskites.^[^ ^105^ ^]^ A further increase in conductivity was achieved by artificially incorporating Sn^4+^ into the structure, confirming their hypothesis. Now there is a consensus that the metallic behavior of tin perovskites originates from heavy p‐type doping, however the distortion of the lattice still determines much of the electronic and optical properties. In the following section we will focus in correlating the electronic and optical properties of layered perovskites to their structure.

##### Band Structure, Density of States, and Effective Masses

**Figure 15:**
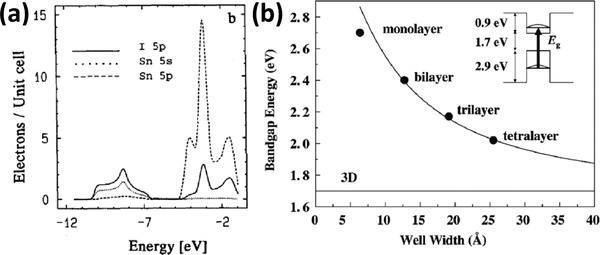
a) DOS assuming only the ionic SnI_4_^2−^ layers as calculated by extended Hückel tight binding method. Reproduced with permission.^[^ ^91^ ^]^ Copyright 1994, Elsevier Ltd. b) The bandgap as a function of the thickness of a layered perovskite, where the solid line is the calculated bandgap based on the effective mass approximation. For a monolayer the approximation breaks down. Reproduced with permission.^[^ ^140^ ^]^ Copyright 2003, Elsevier Science Ltd.

The much larger HOMO–LUMO gap of the organic cations compared to the bandgap of the inorganic octahedral structure gives rise to the fact that the electronic properties near the band edge of low‐dimensional layered perovskites are composed of tin and halide orbitals. The first reported calculations on layered perovskites used this fact effectively by only considering the ionic SnI_4_^2−^ layers to calculate the band structures and DOS of layered tin perovskites using the extended Hückel tight binding method.^[^ ^91^ ^]^ These calculations revealed the salient features of the reduced dimensional structures: a direct bandgap, an increased bandgap, and a reduced bandwidth.^[^ ^138^, ^139^ ^]^ **Figure** **15**a shows the DOS coming out of these calculations and reveals that the valence band maximum (VBM) consists predominantly of antibonding Sn 5s and I 5p orbitals, whereas the conduction band minimum (CBM) is mainly composed of antibonding Sn 5p and I 5p orbitals. The composition of the band edges allows us to understand the impact of the structure on the bandgap, investigated for 〈100〉‐type perovskites by Knutson et al.^[^ ^98^ ^]^ It appears that the in‐plane distortion has the most pronounced effect on increasing the bandgap when compared with the out‐of‐plane distortion. A study involving pentane‐diammonium (PeDA) and iPy as the organic cations revealed that the larger bandgap of (iPy)_2_SnI_4_ was due to the large octahedral distortion, despite a reduced out‐of‐plane distortion.^[^ ^105^ ^]^

These semiempirical calculations are effective in explaining the observed trends; however, one should be careful with over‐interpretation at this level of theory using the effective mass approximation. For example, these calculations overestimate the influence of superlattice effects, where the formation of mini bands is predicted, but not yet observed experimentally with commonly used organic cations.^[^ ^141^ ^]^ Figure 15b shows the results obtained by Tanaka et al., which illustrates that the effective mass approximation used in these calculation fails to reproduce the bandgap for a monolayer (*n* = 1).^[^ ^140^ ^]^ Therefore, especially calculations of the exciton binding energy obtained with these methods are not reliable.^[^ ^91^, ^142^ ^]^ As the effective mass approximation fail for monolayered perovskites, one should use DFT methods for a more accurate description.^[^ ^139^ ^]^ However, here other challenges arise. For example, implementation of DFT structural optimizations should be carefully checked as additional in‐ and out‐of‐plane distortions can significantly impact the electronic structure.^[^ ^139^ ^]^ Second, reliable estimates of effective masses depend on the level of theory, which require implementation of relativistic and many‐body effects. However, for many layered perovskites this level of theory is too computationally demanding, hence quantitative predictions should be treated with caution.^[^ ^139^ ^]^ This lack of reliable estimates results in different values reported for the electron and hole effective mass of (BA)_2_SnI_4_, which have been calculated to be 0.29/0.37 *m*_e_ (HSE + SOC) and 0.33/0.21 *m*_e_ (HSE), respectively.^[^ ^142^, ^143^ ^]^ Strikingly, experimentally determined reduced effective masses of (PEA)_2_SnI_4_ showed lower values compared to FASnI_3_, which is the opposite result if one considers the quantum confinement in this system.^[^ ^144^ ^]^

After taking note of these precautions, calculations can reveal a wealth of information. In the previous section, we discussed that the intercalation of a small solvent molecule, such as dichloromethane, could induce a transition from a 2D layered structure to a 0D structure. DFT band structure calculations at the PBE level of these 0D and 2D layered tin bromide perovskites showed that, as expected, reducing the dimensionality decreases the dispersion and increases the bandgap.^[^ ^108^ ^]^ The band structure calculations of the 0D structure showed that the structurally isolated octahedra are also electronically decoupled. In the 2D case, the lack of dispersion from Γ to μ, corresponding to the out‐of‐plane direction, verifies the 2D electronic nature of the 〈100〉‐type perovskites. These calculations validate the experimental observations that no interaction between adjacent inorganic layers was observed.^[^ ^102^, ^145^ ^]^ Interestingly, the CBM of the 0D structure does include contributions of the organic cation, although the optical properties are still largely determined by the inorganic octahedra with the hole localized on the equatorial Sn–Br plane and the electron localized on the apical Sn—Br bonds.

##### Optical Properties

###### <100>‐Type Perovskites

The dimensional versatility of layered tin perovskites makes the optical properties of these materials highly interesting. The 〈100〉‐type perovskites are often referred to as ideal 2D systems, which are intrinsically excitonic at room temperature. To understand their excitonic nature, we need to consider both the quantum confinement and the dielectric confinement effect. The quantum confinement effect refers to the dimensional reduction of the inorganic layers when sandwiched between large organic cations. This results in the widening of the electronic bandgap with a simultaneous increase of the exciton binding energy (*E*_b_). These two effects partially counteract the effect on the bandgap; however, the net result is a widening of the optical bandgap for low‐dimensional perovskites. For an ideal 2D confined system, one would expect that the *E*_b_^2D^ quadruples with respect of the *E*_b_ in the 3D system due to the increased Coulomb interaction of the charge carriers.^[^ ^146^ ^]^ However, the large difference in the dielectric constant of the inorganic and organic layer results in the experimentally determined *E*_b_ exceeding a factor of four. This enhanced confinement effect is denoted as the dielectric confinement, or image charge effect. Simply put, the lower dielectric constant of the organic layers screens the charge carriers less effectively, which results in an enlarged *E*_b_.

Given that the number of inorganic layers *n* determines the quantum confinement, one can tune the optical bandgap by varying *n*. In the (BA)_2_(MA)*_n_*_‐1_Sn*_n_* I_3_ *_n_*_+1_ series, increasing the confinement from *n* = 5 to 1 shifts the absorption onset from 1.37 to 1.83 eV.^[^ ^134^ ^]^ The pronounced excitonic peak for the *n* = 1 case nicely illustrates the excitonic nature of the 〈100〉‐type perovskites. Besides the number of the inorganic layers and choice of halides, it is the distortion of the inorganic sheets that influences the optical bandgap, much like the electronic bandgap discussed in the previous section.^[^ ^98^ ^]^ This observation might seem obvious at first, however we neglect the impact of the distortion on the *E*_b_. This effect has not been studied in detail, but similar trends in both the optical bandgap and the electronic bandgap upon distortion suggests that the *E*_b_ is not changed significantly. This interesting aspect requires further studies as the large *E*_b_ of hundreds of meV could inhibit the performance of photovoltaic devices. Device applications that require small optical bandgaps should search for layered materials with reduced distortion of the inorganic layers, such as benzimidazolium and benzodiimidazolium with optical bandgaps of 1.81 and 1.79 eV, respectively.^[^ ^147^ ^]^ Much like the 3D perovskites the optical bandgap can be shifted to higher energies when exchanging iodine for bromine.^[^ ^95^, ^109^ ^]^ This interesting feature was already observed in the early 1990s for decylammonium tin halide perovskites, shifting the emission peak from 605 to 517 nm.^[^ ^99^ ^]^ Hence, both the choice of the organic cation as the halide influences the bandgap, offering possibility for tuning in a broad range.

**Figure 16:**
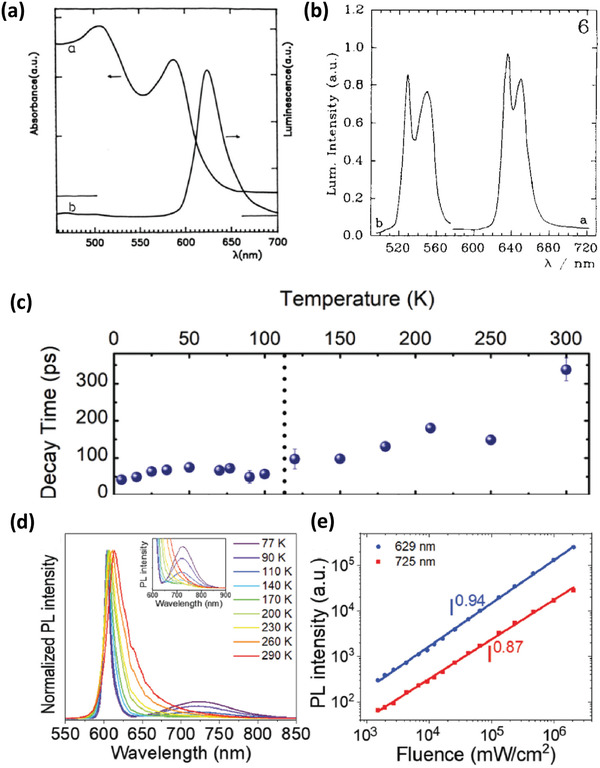
a) Absorbance and PL spectra of PEA_2_SnI_4_ at room temperature. A narrow linewidth is obtained with a shoulder at 650 nm. Reproduced under the terms of the Creative Commons Attribution license.^[^ ^95^ ^]^ Copyright 1993, Royal Society of Chemistry. b) PL of (PEA)_2_SnI_4_ and (PEA)_2_PbI_4_ b at 80 K after several days in air. Reproduced with permission.^[^ ^84^ ^]^ Copyright 1994, Taylor & Francis. c) PL decay time of (PEA)_2_SnI_4_ with decreasing temperature. Reproduced with permission.^[^ ^150^ ^]^ Copyright 2020, The Royal Society of Chemistry. d,e) PL of a stilbene derivative 〈100〉‐type perovskite with decreasing temperature (d) and the power dependence of the two observed emission bands (e).^[^ ^152^ ^]^ Reproduced with permission.^[^ ^152^ ^]^ Copyright 2019, Wiley‐VCH.

An additional strategy to adjust the optical properties of low‐dimensional perovskites includes doping by either homovalent or heterovalent ions. For example, doping of (PEA)_2_SnBr_4_ with Bi^3+^ resulted in a slight blue shift of the PL, in addition to a reduction of the emission from the low energy tail.^[^ ^148^ ^]^ Doping of Mn^2+^ in octylammonium tin bromide resulted in a red shift of the PL present in the pure material, showing orange‐red light.^[^ ^149^ ^]^ Although additional tuning of the optical properties seems possible, these studies show that the PLQY decreases with the introduction of the dopant, hence other dopants should be explored. The absorbance and luminescence of (PEA)_2_SnI_4_ is shown in **Figure** **16**a, a prototypical case of the 〈100〉‐type perovskites, which we will discuss in detail.^[^ ^95^ ^]^ The luminescence shows a small Stokes shift combined with a narrow emission linewidth, a common trait of this class of materials. A closer look at the spectral shape reveals that the luminescence has an asymmetric emission extending to larger wavelengths, with a shoulder at ≈650 nm. In a follow up study, the sample was exposed to air for several days and measured at 80 K as shown in Figure 16b.^[^ ^84^ ^]^ Now the shoulder is clearly resolved and its intensity increased when the temperature decreased, suggesting that defects introduced by the degradation of the sample accounts for the low‐energy emission. The fact that Sn‐based perovskites are rather unstable under light, oxygen, and moisture contributes to this interpretation.^[^ ^84^, ^99^, ^106^ ^]^ A similar observation was made by using butylammonium, thus implying a similar degradation mechanism among all 〈100〉‐type perovskites.^[^ ^92^ ^]^

In 2020, 25 years after the reports discussed above, Folpini et al. reported an intrinsic dark exciton state in (PEA)_2_SnI_4_, laying ≈10 meV under the bright exciton state.^[^ ^150^ ^]^ This intrinsic property has important implications on the relaxation dynamics of the bright exciton state. For instance, the PL decay lifetime is expected to increase when the temperature is lowered. By contrast, the authors observed a reduction of the PL lifetime from room temperature to 100 K, as shown in Figure 16c. This extraordinary behavior can be explained by considering that the dark state has a much slower recombination rate than the bright state. At room temperature, the excitons in the lower‐laying dark state are thermally activated to the bright state causing a delayed radiative recombination, which subsequently results in a long PL lifetime. As the temperature is decreased till 100 K, the back transfer from the lower‐laying dark state to the bright state is impeded, resulting in a sharp decrease of the PL lifetime. Therefore, the authors proposed that the dark state mediates the recombination and acts as a reservoir, supplying excitons for the light emission at room temperature.^[^ ^150^ ^]^ This highlights the importance of fundamental understanding of the light‐emission processes in this material, particularly, as (PEA)_2_SnI_4_ has been applied in light‐emitting devices emitting at 630 nm with a maximum EQE of 0.72%.^[^ ^109^, ^151^ ^]^

A recent study observed a broad emission band at longer wavelength alongside the narrow excitonic emission in 2‐(4‐stilbenyl)ethanammonium tin iodide as shown in Figure 16d.^[^ ^152^ ^]^ Due to the increase of the broad emission at lower temperatures, the authors attributed the emission to self‐trapped excitons, following a recent trend in Pb‐based perovskites.^[^ ^153^ ^]^

Figure 16e shows the power dependence of the narrow and broad emission. In contrast to the authors’ claim of self‐trapped exciton emission, one could argue that the power dependence of the broad emission, which is significantly smaller than one, could also point to defect related emission.^[^ ^154^ ^]^ This extrinsic nature of the PL has been observed in a variety of 〈100〉‐type lead iodide perovskites.^[^ ^155^, ^156^ ^]^ Interestingly, 〈100〉‐type lead perovskites have shown more interesting properties, such as biexciton emission and hot‐exciton emission.^[^ ^96^, ^157^, ^158^ ^]^ The similarity of the optical properties of tin‐ and lead‐based compounds might indicate that more interesting properties are waiting to be discovered in tin‐based systems, which will simultaneously advance our understanding also of lead‐based perovskites.

###### Quasi‐2D Perovskites

**Figure 17:**
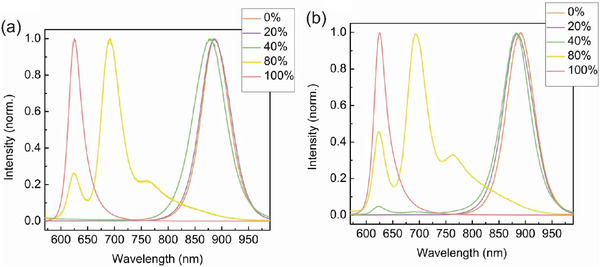
PL spectra of films with different PEA ratios when excited a) from the front surface and b) from the back surface. Reproduced with permission.^[^ ^160^ ^]^ Copyright 2017, American Chemical Society.

By design, the quasi‐2D structures allow to tune the absorption and PL emission. For example, Liao et al. noticed a blue shift from 890 to 625 nm of the photoluminescence peak with increasing PEA ratio due to the formation of low‐dimensional perovskites (**Figure** **17**a,b).^[^ ^159^, ^160^ ^]^ These (PEA)_2_(FA)*_n_*_‐1_Sn*_n_* I_3_ *_n_*_+1_ perovskite films were excited from both the front (perovskite/air interface) and the back surface (perovskite/substrate interface).^[^ ^160^ ^]^ For 40% PEA, the perovskite films showed three distinct PL peaks at 625, 693, and 762 nm at the back surface, which are signatures of low‐dimensional perovskites with *n* equal to 1, 2, and 3, respectively. However, the measurements from the front did not show emission peaks at 625 or 693 nm. Consequently, the authors argued that the 2D layers prefer to locate at the back of these films. Thus, it is clear that these quasi‐2D films do not form phase pure films, their formation and structure will be discussed in detail in the next section.

The use of large organic cations in small concentrations, as additives, generally only has a minor, but important, effect on the optical properties of perovskites. Liao et al. deposited a small amount of phenethylammonium bromide (PEABr) on top of FASnI_3_, resulting in a larger bandgap of 1.4 eV compared to the 1.34 eV of the pristine material.^[^ ^161^ ^]^ The photoluminescence spectra of these films revealed stronger photoluminescence intensity with a clear blue shift, which can be attributed to the formation of a low‐dimensional perovskite phase. Kayesh et al. studied the impact of the additive 5‐ammonium valeric acid iodide and did not observe a change in the absorption band edge or PL peak position, however a reduction of nonradiative recombination was observed, probably due to a passivation of the surface defect and of the grain boundaries.^[^ ^162^ ^]^ Similarly, Jokar et al. reported no change in the optical bandgap when using butylammonium iodide (BAI), however a blue shift was observed using ethylenediammonium diiodide (FASnI_3_–yEDAI_2_).^[^ ^163^ ^]^

**Figure 18:**
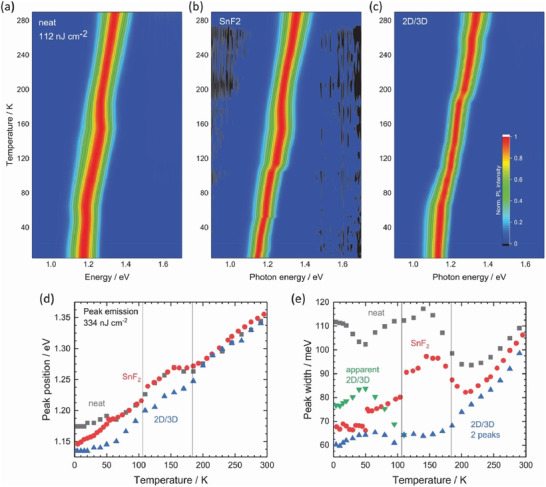
Normalized temperature dependent PL spectra of a) FAnI_3_, b) the SnF_2_, and c) the 2D/3D sample. d) Peak position and e) width of the three samples versus the temperature. Reproduced under the terms of the Attribution Non‐Commercial No Derivatives 4.0 International license.^[^ ^74^ ^]^ Copyright 2019, Wiley‐VCH.

Shao et al. found that adding a small quantity of PEAI in FASnI_3_ does not affect the absorption onset of the perovskite material. However, the mixed dimensional perovskite exhibited a higher extinction coefficient and refractive index than the 3D film probably due to its superior crystallinity.^[^ ^164^ ^]^ Similarly, Kim et al. found that the optical bandgap was not affected by the addition of PEAI or FASCN, highlighting that quantum and dielectric confinement effects with the low concentration of long organic cations are negligible.^[^ ^35^ ^]^ Kahmann et al. compared the photophysics of 3D and 2D/3D films, without clear low‐dimensional PL signatures, from room temperature to 4.3 K (**Figure** **18**).^[^ ^74^ ^]^ The 2D/3D sample exhibited a slightly red‐shifted and narrower emission peak at room temperature due to decreased scattering by the background ions and carriers compared to the 3D film, which suggests a superior crystalline quality of the 2D/3D film. This hypothesis was supported by the two orders of magnitude higher PL lifetime of the 2D/3D film at low excitation fluences as well as higher lifetimes for the hot carrier PL at high excitation fluences compared to the 3D films. Generally, both films showed a similar reduction of the bandgap with decreasing temperature, where the 2D/3D film exhibited a more pronounced red shift. The temperature‐dependent steady‐state PL spectra indicate three (possibly four) phase transitions for all the films. Initially the linewidth decreases with the decreasing temperature. Toward the orthorhombic–orthorhombic phase transition at 180 K, the 2D/3D film exhibited a reduced peak width due to suppressed phase inhomogeneity possibly caused by the templating effect of the 2D phase, which is opposed to the distinct widening of 3D film. When the temperature is below 110 K, all samples give rise to different trends. Unlike the 3D film, which shows a strong reduction of the linewidth around 110 K and further decreases to ≈70 meV below 50 K, the 2D/3D sample shows a pronounced broadening of the emission, which varies from 65 meV at 110 K to 83 meV at 50 K. This apparent widening of the PL peak is due to the emergence of a secondary peak at lower energy, of which the origin is still matter of speculations.^[^ ^74^ ^]^

###### 〈110〉‐Type Perovskites

Stoumpos et al. discovered important structure–optical properties relation in 〈110〉‐type perovskites, essential for advancing our understanding of this interesting class of materials.^[^ ^106^, ^108^ ^]^ For *n* = 1 in the 〈110〉‐type perovskites, the structure consists of 1D chains instead of the 2D layers of the 〈100〉‐type, hence a widening of the optical bandgap of these materials is observed. For *n* = 2, the 1D chains are connected to form corrugated 2D sheets as discussed for (GA)_2_SnI_4_. Interestingly, a significantly larger bandgap is observed for (GA)_2_SnI_4_ than for (BA)_2_SnI_4_, a 〈100〉‐type perovskite. The authors proposed that the corrugation of adjacent inorganic chains will reduce the orbital overlap, which in turn opens the bandgap.^[^ ^106^ ^]^

###### 0D Perovskites

**Figure 19:**
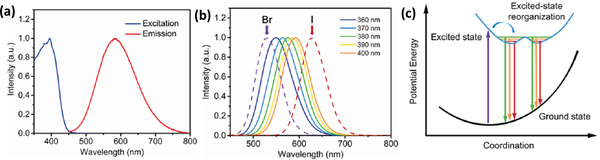
PL excitation and emission of mixed iodine/bromine a) at room temperature and b) at 77 K. Changing the excitation wavelength changes the emission. The dotted lines are the pure iodine and bromine samples. c) Proposed mechanism of the tunable emission. Reproduced with permission.^[^ ^125^ ^]^ Copyright 2017, American Chemical Society.

As expected, the optical bandgap of 0D perovskites is widened compared to higher dimensional structures discussed above. **Figure** **19**a shows a typical emission spectrum of 0D perovskites.^[^ ^125^ ^]^ The emission features a large Stokes shift, large FWHM, PLQY close to unity, and PL decay lifetimes up to several microseconds.^[^ ^108^, ^113^, ^125^, ^126^, ^127^, ^129^, ^130^, ^131^, ^165^ ^]^ The facile synthesis, high stability, and high PLQY of these materials reveal a promising alternative to rare‐earth phosphors for white‐light applications.^[^ ^111^, ^125^, ^127^ ^]^ DFT calculations indicated that excitation of the 0D material strongly deformed the isolated octahedra, elongating and contracting the tin halide bonds, whereby the exciton is fully localized on the inorganic structure.^[^ ^108^, ^126^ ^]^ This localization means that the exciton becomes self‐trapped, which subsequently emits light with a large Stokes shift and FWHM. Figure 19b shows the emission from a 50/50 mixed iodine/bromine after excitation at wavelengths ranging from 360 to 400 nm.^[^ ^125^ ^]^ Interestingly, the emission wavelength depends on the excitation wavelength at 77 K, while this dependence was absent for the pure bromine and iodine samples. The authors proposed that the excitation energy dependence is the result of the structural deformation of either Sn—Br or Sn—I bonds, schematically depicted in Figure 19c. These two deformations could be selectively excited at 77 K, as thermal energy is insufficient to reach a thermally activated equilibrium, whereas at room temperature this dependence could not be observed as sufficient thermal energy is present to reach equilibrium. This equilibrium of distorted structures is reflected in the larger FWHM of the mixed halide compared to its pure halide counterparts.

We note that there are several examples of 〈100〉‐type tin bromide perovskites showing luminescence which closely resembles the emission features of 0D perovskites, suggesting a common origin.^[^ ^110^, ^111^, ^166^ ^]^ Others, reported no such emission in (BA)_2_SnBr_4,_ suggesting that the zero‐dimensionality of these structures is a prerequisite for this type of emission.^[^ ^129^ ^]^ We note that the broad emission is observed across many types of metal halide perovskites, but it remains unclear if a common origin can be accounted for it or whether other factors, such as dimensionality or extrinsic factors as defects play an important role.^[^ ^167^ ^]^

To conclude this section, the large variety of compositions and structures of low‐dimensional layered perovskites enables endless options to tune the optical properties and to investigate the structural optical properties relation. Much of the optical properties of tin‐based compounds, of which many of the structures have been determined, have not been studied in detail or their optical properties are not well understood. We foresee that in the near future, as an increasing number of researchers are focusing on these systems, their properties will be unraveled.

## 5 Crystallization and Film Formation

Understanding the crystallization dynamic is critical for the formation of high‐quality perovskite thin films. Sn‐perovskites undergo nonuniform nucleation and growth due to faster crystallization process as compared to Pb‐based materials. Such a fast crystallization process is prone to produce structural defects such as vacancies, pinholes, open grain boundaries, as well as orientation and stacking disorder of the grains. These structural defects cause an increase in nonradiative recombination centers and may determine short circuits and low *V*_oc_ in solar cells. The key to realize high quality tin perovskite films is through the understanding of the dynamic crystallization process, which gives rise to possible strategies to control it. Unlike the numerous studies that appeared in the last few years on the thin film formation of Pb‐based perovskites, systematic investigations about the crystallization mechanism of Sn ‐based perovskites thin film are scarcely reported mostly due to the difficulty in studying their intrinsic properties due to the facile oxidation of Sn^2+^.^[^ ^168^, ^169^ ^]^

### 5.1 3D Perovskites

**Figure 20:**
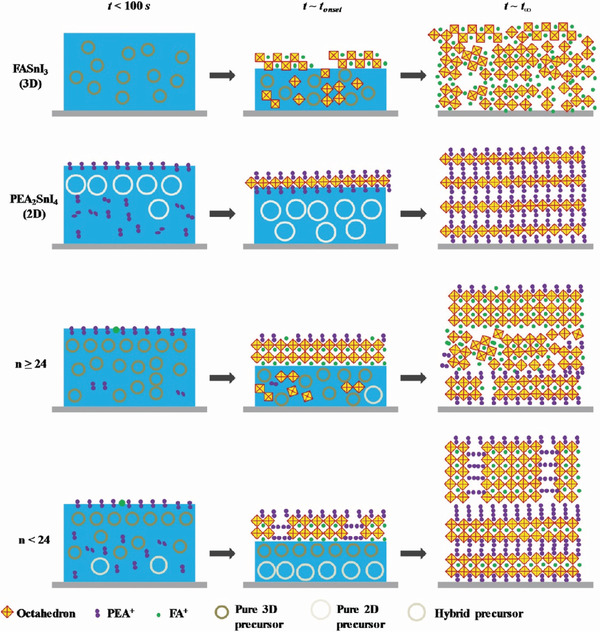
Schematic of the crystallization process of FA‐based Sn‐perovskites films deposited by spin‐coating using different concentrations of PEA. Reproduced under the terms of the Creative Common Attribution 4.0 International license.^[^ ^169^ ^]^ Copyright 2020, Wiley‐VCH.

There is an ongoing debate about the crystallization process of FASnI_3_, which seems to be dependent on the spacer cations, the additives, and the substrates. Some groups reported that the nucleation and crystallization of the 3D perovskite initiated from the substrate–solution interface, while others reported a process starting from the solution–air interface.^[^ ^76^, ^77^, ^78^ ^]^ Dong et al. for the first time systematically monitored the structural evolution of FASnI_3_ during the spin‐coating process, which was performed in a nitrogen protected atmosphere at room temperature, by using in situ grazing incidence wide‐angle X‐ray scattering (GIWAXS) technique.^[^ ^169^ ^]^ In the first stage, the crystallization process starts at the liquid/air interface from a disordered precursor solution due to the oversaturation of the ionic species as the solvent evaporates, which provides the anisotropic environment for oriented nucleation and growth. In the second stage, the significant bulk crystallization of FASnI_3_ led to isotropic nucleation and growth, introducing random orientation of the 3D grains. The authors found that 2D and quasi‐2D compounds with *n* ≤ 24, using the long organic cation PEA^+^, crystallized predominantly in a surface‐controlled manner, leading to highly oriented grains. The authors argued that the long organic cation promotes the surface crystallization and suppresses the bulk crystallization. Interestingly, at the interface with the substrate, a quasi‐2D phase with *n* = 2 is found that grows parallel to the substrate and is the last to crystallize. **Figure** **20** graphically describes these different growth mechanisms. Similarly, Meng et al. reported a surface‐controlled growth of FASnI_3_ by incorporating a small quantity of a tailor‐made fluorinated organic cation, pentafluorophen‐oxyethylammonium iodide (FOEI), which reduced the surface energy of the solution/air interface and served as a template to link SnI_6_^4−^ monomers for further crystal nucleation and growth.^[^ ^170^ ^]^ Consequently, adding a small amount of FOEI molecules creates highly crystalline and oriented films.

The one‐step method is a widely used technique to deposit perovskite thin films.^[^ ^171^ ^]^ When a solvent with a low or moderate boiling point is used, Sn‐perovskite crystallizes during the depositing process. Hao et al. demonstrated the crystallization and film formation of MASnI_3_ by controlling the solvent evaporation rate during the film deposition process, which allowed them to deposit well‐crystallized films due to the high enthalpy of crystallization.^[^ ^34^ ^]^ Moreover, the authors reported pinhole‐free films using a strongly coordinated solvent such as dimethyl sulfoxide (DMSO), where a stabilized transitional SnI_2_·3DMSO intermediate phase allowed for control of the crystallization process.^[^ ^34^ ^]^ MASnI_3_ films deposited from DMF showed inhomogeneous surface coverage with pinholes due to the fast crystallization process during spin‐coating. Even though *N*‐methyl‐2‐pyrrolidone was shown to slow down the crystallization of the film, which crystallized only upon thermal annealing, however, no significant improvement in the film coverage was obtained. This revealed that the intermediate phase with DMSO is an important transitional step toward uniform and high surface coverage thin films.

Since it is challenging to achieve smooth and pinhole free surfaces by conventional one‐step spin‐coating method, other deposition methods had to be developed, among them we have the antisolvent, the two‐step spin‐coating, and the thermal evaporation methods. Liu et al. investigated the influence of the antisolvents diethyl ether, toluene, and chlorobenzene, on the tin perovskite film morphology.^[^ ^172^ ^]^ Dense and uniform Sn‐based perovskite film were achieved using chlorobenzene. A so‐called hot antisolvent method by heating the antisolvent further improves the surface coverage, the grain size, and the uniformity of the perovskite film.^[^ ^173^ ^]^ Unlike the conventional one‐step method, a solvent–solvent extraction method consists of dipping the spin‐coated wet film directly in an antisolvent bath, produced MASnI_3_ films with a roughness of 7.5 nm with crystals oriented along the 〈001〉 direction.^[^ ^174^ ^]^ Beside the aforementioned solution processes, thermal evaporation was also used to get a high uniformity and full coverage of Sn‐based perovskite films.^[^ ^175^ ^]^ The vapor‐assisted process is a combination of a dip‐ or spin‐coating technique with vapor deposition. This method has the potential to create large perovskite crystallites with a smooth morphology, with bigger grain size of >200 nm and improved stability respect to the one‐step methods.^[^ ^176^, ^177^ ^]^ For example, the vacuum‐assisted solution processing (VASP) method, which involves spin‐coating of the perovskite precursor solution to obtain an intermediate phase and then transferring the wet film in a vacuum chamber for controlled crystallization, has been successfully used to deposit Pb‐based perovskite films.^[^ ^178^, ^179^ ^]^ Li et al. applied the VASP method to fabricate CsSnI_3_ perovskite films.^[^ ^180^ ^]^ First, they spin‐coated a SnI_2_ solution with 30 mol% of SnF_2_ onto a mesoporous TiO_2_ substrate. After thermal annealing, films were transferred into the vacuum chamber for CsI deposition followed by a second annealing step to establish a good interdiffusion of the precursors. During this last processing step, the films turned from yellow to black, indicating that SnI_2_ and CsI can only react at high temperatures. Through SEM characterization of the films, they found that the thickness of evaporated CsI is a key parameter to fabricate good quality films. When the CsI film is too thin (≈40 nm) there is not enough CsI to compensate for SnI_2_, leading to the presence of pinholes. On the other hand, when the CsI film thickness is too high (≈80 nm), white impurities assigned to CsI emerge. The density of these impurities increased as the thickness of CsI further reached 100 nm. An optimum CsI thickness of 66 nm leads to highly oriented CsSnI_3_ films with significantly reduced size and quantity of pin holes with respect to the one‐step spin‐coating method.^[^ ^181^ ^]^ Besides these main processes, other techniques such as solvothermal reaction and melt synthesis have been implemented.^[^ ^182^, ^183^, ^184^ ^]^

### 5.2 Layered Perovskites

Layered perovskites are highly versatile in terms of deposition methods, which can be used for their deposition in thin films. Among the most used of these techniques are: spin coating, doctor‐blade coating, dip coating, thermal evaporation, and thermal ablation.^[^ ^145^, ^169^, ^185^, ^186^, ^187^ ^]^ In the late 1990s various of these deposition methods were explored. A dip‐coating method used two steps for deposition: first a layer of SnI_2_ was evaporated, which was followed by dipping the thin film in a solution containing the large organic cation.^[^ ^145^ ^]^ The authors reported formation of the layered perovskite within seconds, although these films were much rougher compared to spin‐coated films.

**Figure 21:**
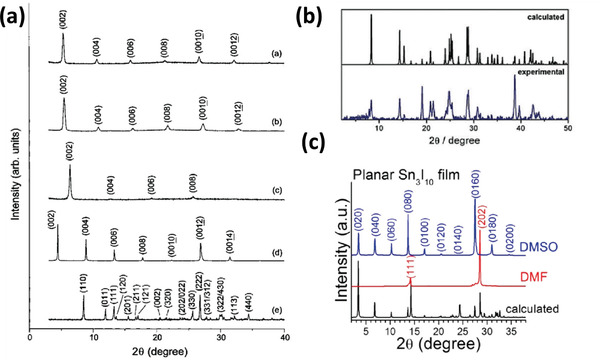
(a) Highly oriented thin films obtained by thermal ablation. a) (PEA)_2_PbBr_4_, b) (PEA)_2_PbI_4_, c) (BA)_2_SnI_4_, d) (BA)_2_(MA)Sn_2_I_7_, and e) NH_3_(CH_2_)_6_NH_3_BiI_5_. Reproduced with permission.^[^ ^145^ ^]^ Copyright 1999, American Chemical Society. (b) Thin films of benzodiimidazolium tin iodide show a much more isotropic orientation of the crystal domains compared to PEA and BA. Reproduced with permission.^[^ ^147^ ^]^ Copyright 2019, Wiley‐VCH. (c) *n* = 3 thin films using BA and MA cations deposited with pure DMSO or DMF. The orientation of the layers is dependent on the solvent used. Reproduced with permission.^[^ ^134^ ^]^ Copyright 2017, American Chemical Society.

Although spin‐coating is an excellent method for small‐scale laboratory research, it is not suitable for large‐scale production or coating textured surfaces. In addition, wetting issues can arise from these solution‐based methods. Vacuum deposition techniques could be an interesting alternative to obtain dense and smooth films.^[^ ^187^ ^]^ However, a challenge can arise if the organic salts can decompose during slow heating, hence ablation of the materials by flash evaporation is an interesting alternative.^[^ ^186^ ^]^ XRD patterns of thin films obtained by this ablation method are shown in **Figure** **21**a. Small grain sizes were obtained (<75 nm) with layer thicknesses between 10 and 200 nm, depending on the amount of starting material. High intensity reflections of the (001) peaks were measured, showing that these layered perovskites form highly oriented layers with the inorganic layers parallel to the substrate. This commonly observed parallel orientation of layered perovskites is beneficial for thin film transistors but can prevent charge transport in the perpendicular direction for solar cells and LEDs. For this reason, increased efforts were directed to obtain the preferential vertical orientation. In a study using benzodiimidazolium as the large cation, the layered tin perovskite thin film did not show a preferential alignment (Figure 21b). It was suggested that the increased hydrogen bonding was the cause of the isotropic crystallization.^[^ ^147^ ^]^ The effect of solvents was studied by Cao et al. by fabricating thin films from either pure DMSO or pure DMF solutions.^[^ ^134^ ^]^ Figure 21c shows that films casted from a DMSO solution resulted in layers parallel to the substrate, while deposition from a DMF solution resulted in a perpendicular growth of the layers. In addition, the authors could obtain phase pure layers of *n* = 4 by casting thin films from solvation of phase‐pure single crystals, which suggested that “perovskite seeds” in the solution can direct the crystal growth.

The fast crystallization of Sn‐based perovskites often imposes challenges to obtain smooth and dense films. For example, poor surface coverage was observed with the use of butylammonium.^[^ ^188^ ^]^ This problem could be solved by introducing the ion liquid methylammonium acetate into the solvent mixture, resulting in full surface coverage with a large average grain size of 9 µm. Others have shown that phenethylammonium allows for dense and uniform films when deposited from precursors dissolved in DMSO.^[^ ^164^ ^]^ The faster crystallization of thin films also allows for the growth of various structures and compositions that could not be obtained otherwise. For instance, perovskite films based on 2,3,4,5,6‐pentafluorophenethylammonium and 2‐naphthyleneethylammonium in various ratios could be obtained, whereas in single crystals only a 1:1 ratio could be obtained.^[^ ^189^ ^]^ Others showed that the faster crystallization could impose a 3D vacant structure instead of the thermodynamically stable 〈100〉‐type perovskites normally obtained by using hydroxyethyl ammonium as the organic cation.^[^ ^190^ ^]^

### 5.3 Quasi‐2D

In general, it is arduous to synthesize phase‐pure quasi‐2D, perovskites with a fixed *n*‐value. The fabricated quasi‐2D perovskite film normally consists of a mixture of different *n* phases. Myae Soe et al. found that the highest *n* number for a phase pure quasi‐2D structure is *n* = 7 either as film, powder or single crystals.^[^ ^135^ ^]^ The authors suggested that this difficulty of obtaining phase pure materials could originate from the similar formation enthalpies of the distinct *n*‐values, particularly in the case of high *n* number. In case of a large difference in the formation enthalpy, the formation of *n* number phases with the lowest formation enthalpy will be favored. In addition, the distribution of multiple phases in a sample is rarely in an ordered arrangement, which is detrimental for the performances of devices made with them. It is important to underline, that when the *n*‐value increases, the proportion of the large cation decreases, and the formation of 3D phases becomes noticeable. In general, the formation of lower dimensional phases is not proportional with the *n*‐value. The *n* = 2 and 4 phases have the smallest formation enthalpy among the *n* < 6 numbers, while the *n*‐values of 1, 3, and 5 have higher formation enthalpies than the *n* = 2 and 4 phases.^[^ ^135^ ^]^ For this reason, the resultant films form heterojunction structures. Liao et al. reported one of the first low‐dimensional Sn‐based perovskite films, implementing PEA on the A′‐site.^[^ ^160^ ^]^ They found that the FASnI_3_ bulk perovskite is separated into low‐dimensional nanolayers ((PEA)_2_(FA)*_n_*_‐1_Sn*_n_* I_3_ *_n_*_+1_), where the average *n* value could be tuned by changing the stoichiometric ratio of PEA to FA from 0% to 100%. The authors found two peaks near 14° in XRD pattern of the mixed FA‐PEA samples, assigned to the multilayer 2D components (*n* > 5). The 0% PEA sample displayed Debye–Scherrer rings with an isotropic intensity distribution, indicating complete random orientation of the sample grains. On the contrary, in the 20% sample sharp and discrete Bragg spots are observed, which indicate that crystal grains are highly oriented, with their (10$\bar{1}$) plane parallel to the substrate. Films with higher PEA ratios showed diffraction rings with stronger intensity in the *q*_z_ direction, which indicated the preferential orientation of PEA bilayers parallel to the substrate.

**Figure 22:**
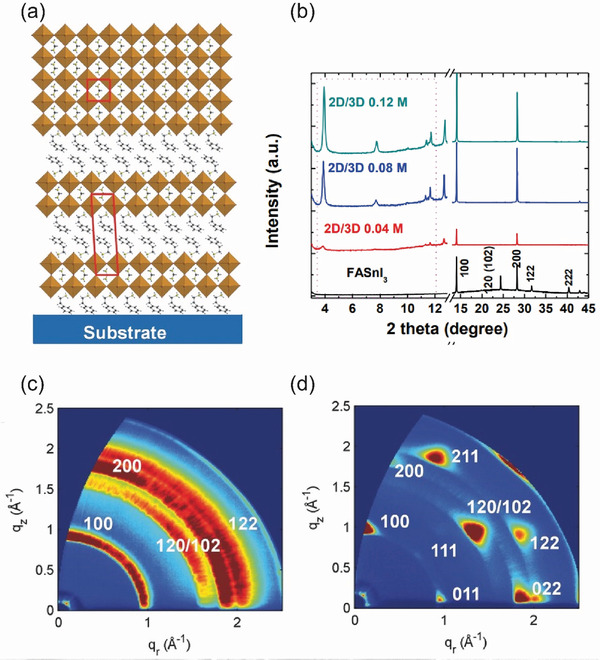
a) Schematic crystal structure of 2D/3D mixture (2D 0.08 m). b) XRD patterns of FASnI_3_ films with different concentration of 2D Sn perovskite (the diffraction intensity of the reference film and the diffraction intensity in the 2θ < 12° region for the 2D/3D mixed films are magnified by a 100 times). GIWAXS images recorded at an incident angle of 0.25° of c) 3D reference and d) 2D/3D mixture. (a–d) Reproduced under the terms of the Attribution Non‐Commercial No Derivatives 4.0 International license.^[^ ^164^ ^]^ Copyright 2018, Wiley‐VCH.

In another work, Shao et al. added much lower concentrations of PEAI (from 0 to 0.16 m) to FASnI_3_ to form 2D/3D structures **Figure** **22**a.^[^ ^164^ ^]^ In these samples the authors observed dominant (100) and (200) peaks at angles of 14.0° and 28.22° with enhanced intensity with respect to the 3D reference sample (Figure 22b). Moreover, the suppression of the (120)/(102), (122) and (222) peaks, revealed enhanced crystallinity and preferential crystallization of the 3D phase with the (*h*00) planes parallel to the substrate. The weak peaks at 2θ values <12° indicated the presence of the 2D perovskite with double layers of SnI_6_ octahedra in the thin film (Figure 22a). The reference 3D FASnI_3_ film exhibited Debye–Scherrer‐like rings (Figure 22c) due to random orientation of the grains. By contrast, the 2D/3D films showed well‐defined Bragg spots due to the preferential orientation of the 3D phase with respect to the substrate (Figure 22d).

The two diffraction peaks originating from the 2D phase become visible only when X‐rays achieve full penetration of the film, suggesting that the 2D phase is in the proximity of the substrate. Wang et al. introduced a removable pseudohalogen, ammonium thiocyanate (SCN), to manipulate the crystal growth process of PEA*_x_* FA_1‐_ *_x_* SnI_3_ perovskite films.^[^ ^191^ ^]^ They demonstrated that the addition of SCN separates the nucleation and crystallization growth processes, leading to the formation of a 2D–3D hierarchy structure. GIWAXS measurements showed that the SCN addition formed a 2D‐quasi–2D–3D structure with a parallel orientation of the layers with respect to the substrate. The GIWAXS spectra of the PEA_0.15_FA_0.85_SnI_3_ film indicated the absence of single‐layer perovskite (PEA_2_SnI_4_), while a quasi‐2D perovskite (PEA_2_FASn_2_I_7_) dominated the surface. In order to get a higher degree of crystallinity in the out‐of‐plane direction for PEA*_x_* FA_1‐_ *_x_* SnI_3_, Kim et al. added formamidinium thiocyanate (FASCN) as an additive.^[^ ^35^ ^]^ Compared to the 10% PEAI film without additive, more distinct and clear Bragg spots of the (100) and (200) planes were observed, implying enhanced crystal orientation with respect to the direction perpendicular to the substrate. The improved crystal orientation in the out‐of‐plane direction is essential for charge transport in diode like devices (vide infra). Jokar et al. showed that butylammonium iodide, another large organic cation, enhanced the crystallinity and orientation along (100) and (200) planes.^[^ ^163^ ^]^

### 5.4 Defect Physics

Solution processed Sn‐based perovskites are prone to form point defects such as vacancies, interstitial atoms, and antisite substitutions. The type of the dominant defects, which determine the physical properties of tin perovskite films, depends on the growth conditions, the A cations as well as the X halides. This section is aimed to gain insight into the defect physics of FASnI_3_, MASnI_3_, and CsSnI_3_ by discussing the role of the growth conditions, organic cations, as well as halide anions in the defect formation and photophysical and chemical properties of these materials.

**Figure 23:**
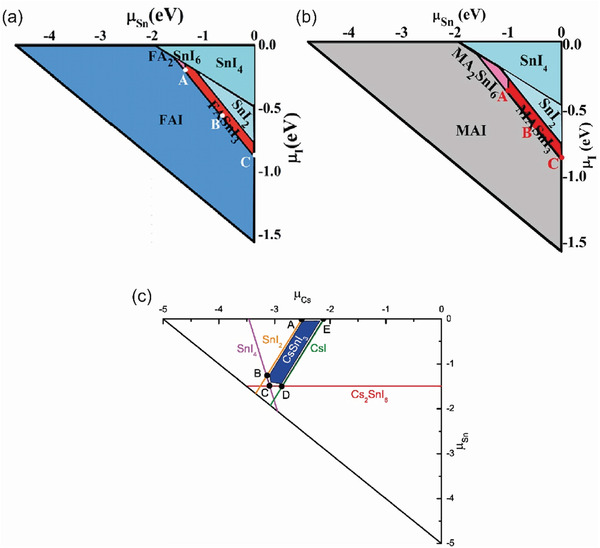
Calculated phase maps with chemical potential region (in red for MASnI_3_ and FASnI_3_ and blue for CsSnI_3_) that stabilizes perovskite phase against other competitive phases for a) MASnI_3_, b) FASnI_3_, c) CsSnI_3_, and d) Cs_2_SnI_6_. (a,b) Reproduced with permission.^[^ ^64^ ^]^ Copyright 2017, The Royal Society of Chemistry. (c) Reproduced with permission.^[^ ^193^ ^]^ Copyright 2014, American Chemical Society.

Gaining insight into the phase maps of MASnI_3_, FASnI_3_, and CsSnI_3_ is very important for understanding how to obtain clean phases of these materials, as shown in **Figure** **23**. In these maps the chemical potential of different compound elements (Sn, I, Cs) is reported on *x*‐ and *y*‐axes. The chemical potential is strictly related to the formation energy of the different phases, and through these maps we can better understand which could be the optimum processing condition window available for that specific material to avoid the formation of the undesirable secondary phase. Shi et al. compared MASnI_3_ and FASnI_3_ and observed that the two organic cations play an important role in the defect properties of Sn halide perovskites.^[^ ^64^ ^]^ The thermodynamic stability regions for the two materials are filled in red in Figure 23a,b.

These regions are very narrow thus a strict control of the synthetic conditions is required to avoid the formation of secondary phases of SnI_2_, SnI_4_, FAI, and FA_2_SnI_6_. The region of thermodynamic stability has been obtained considering the thermodynamic equilibrium condition, according to which the existence of FASnI_3_ should satisfy:

(5) $$\mu_{\text{FA}}+\mu_{\text{Sn}}+3\;\mu_{I}=\;\Delta H_{f}\;\left({\text{FASnI}}_{3}\right)=\;-4.67\;\text{eV}$$

where μ_FA_, μ_Sn_, and μ_I_ are the chemical potential of the various components referring to the most stable phase, and Δ*H*_F_(FASnI_3_) is the formation enthalpy of the perovskite material. This equation gives information on the value of the chemical potential of the compounds involved in the perovskite system. To avoid the formation of the secondary phases of SnI_2_, SnI_4_, FAI, and FA_2_SnI_6_, the following equations must be satisfied:

(6) $$\mu_{\text{FA}}+\mu_{I}<\Delta H_{f}\;\left(\text{FAI}\right)=\;-2.91\;\text{eV}$$

(7) $$\mu_{\text{Sn}}+2\mu_{I}<\Delta H_{f}\;\left({\text{SnI}}_{2}\right)=\;-1.55\;\text{eV}$$

(8) $$\mu_{\text{Sn}}+4\mu_{I}<\Delta H_{f}\;\left({\text{SnI}}_{4}\right)=\;-1.94\;\text{eV}$$

(9) $$2\mu_{\text{FA}}+\mu_{\text{Sn}}+6\mu_{I}<\Delta H_{f}\;\left({\text{FA}}_{2}{\text{SnI}}_{6}\right)=\;-7.98\;\text{eV}$$

The narrow chemical range is partially cut by the competing SnI_4_ and FA_2_SnI_6_ phases. The thermodynamically stable region of CsSnI_3_ is also narrow, limited by other competitive elemental phases involving Cs, Sn, and I and the unwanted secondary phases (SnI_4_, Cs_2_SnI_6_) (Figure 23c).^[^ ^193^ ^]^ It is the chemical environment that triggers the formation of unwanted secondary phases. For example, a Cs‐excessive environment favors the formation of CsI, while a Cs‐deficient environment promotes the formation of SnI_2_ and SnI_4_ phases.

**Figure 24:**
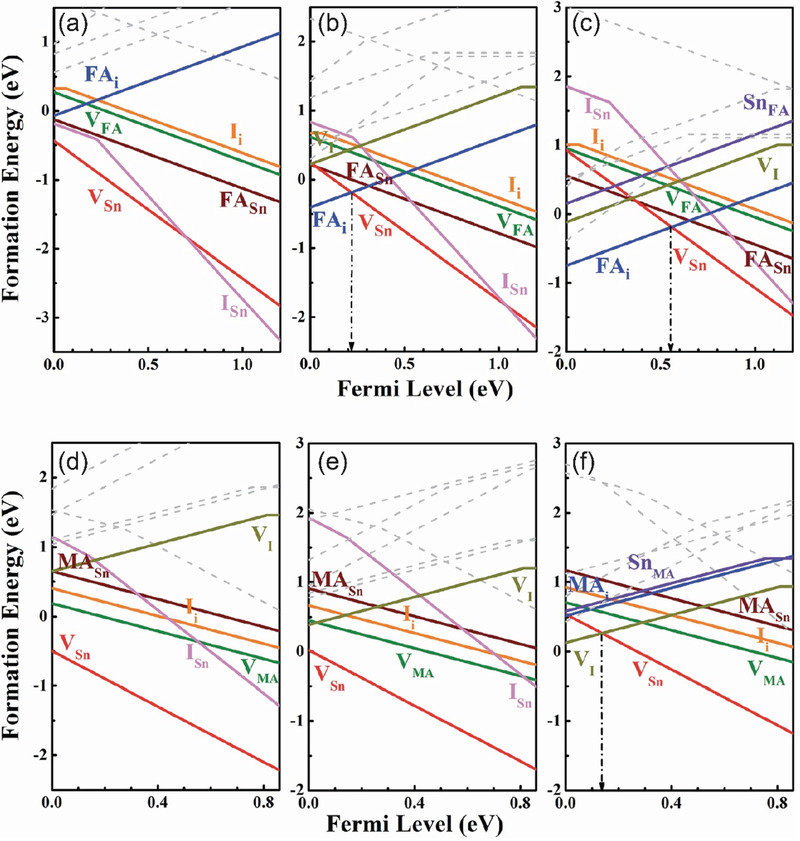
a–c) The formation energies of intrinsic point defects in FASnI_3_ under different chemical potential conditions. A – I‐rich/Sn‐poor condition (a), B – moderate condition (b), and C – I‐poor/Sn‐rich condition (c), and the formation energies of intrinsic point defects in MASnI_3_ at different chemical potential d) A, e) B, and f) C. Gray dashed lines represent the remaining defects with high formation energies. (a–f) Reproduced with permission.^[^ ^64^ ^]^ Copyright 2017, The Royal Society of Chemistry.

As mentioned, three kinds of defects are important to be considered: atomic vacancies, interstitial atoms, and antisite substitutions. For FASnI_3_ specifically, we need to consider FA, Sn, and I vacancies (*V*_FA_, *V*_Sn_, and *V*_I_), FA, Sn, and I interstitials (FA_i_, Sn_i_, and I_i_), FA on Sn and Sn on FA cation substitutions (FA_Sn_ and Sn_FA_) and four antisite substitutions (FA on I (FA_I_), Sn on I (Sn_I_), I on FA (I_FA_), and I on Sn (I_Sn_)). The formation energies of these defects depend on the chemical environment in which the perovskite film is deposited. In Figure 23a,b, three main conditions are considered: I‐rich/Sn‐poor (point A), I‐poor/Sn‐rich (point B), and moderate concentration of I and Sn (point C). For these three conditions, the formation energies versus the Fermi level energy are shown in **Figure** **24**a–c. Under point A and B conditions (Figure 23b), the electron acceptor defects such as Sn vacancies have much lower formation energy than that of electron donor defects such as FA_i_, which leads to a significant amount background holes in FASnI_3_ and pins the Fermi energy to the VBM.^[^ ^64^ ^]^ At the C point, FA interstitial defects, which form due to the weak van der Waals interaction between the organic molecule FA and Sn–I framework, compensate for the Sn vacancies. Consequently, the Fermi level is pinned at the middle part of the bandgap and FASnI_3_ behaves as an intrinsic semiconductor. These results clarify that the defect properties and conduction behavior of FASnI_3_ perovskite depend on the growth conditions.

In the case of MASnI_3_, there are also 12 possible intrinsic defects: three vacancies *V*_MA_, *V*_Sn_, and *V*_I_; three interstitial defects MA_i_, Sn_i_, and I_i_, and six substitutions MA_Sn_, Sn_MA_, FA_I_, Sn_I_, I_FA_, and I_Sn_.^[^ ^64^ ^]^ Among these point defects, *V*_Sn_ is always the dominant defect of MASnI_3_ independently on the growth conditions due to the lowest formation energy, leading to p‐type conduction (Figure 24d–f). It is worth mentioning that Sn vacancies in MASnI_3_ films have lower formation energy (more negative) than that of FASnI_3_ due to stronger s–p antibonding coupling of the shorter Sn—I bond. This explains the higher density of Sn vacancies in this system, which results in a higher density of background hole density in the MASnI_3_ when compared to FASnI_3_.

Similarly, the important defects for CsSnI_3_ include vacancies as *V*_Cs_ *V*_Sn_, *V*_I_, interstitial Cs_i_, Sn_i_, and I_i_, and antisite substitution Cs_Sn_, Sn_Cs_, Cs_I_, I_Cs_, Sn_I_, and I_Sn_.^[^ ^193^ ^]^ Moving from Sn‐rich to Sn‐poor conditions, the formation energy of the electron acceptor states decreases, while the formation energy of the electron donor states increases. This shows that the chemical environment affects the Fermi level and the conduction behavior. Under Sn‐rich conditions, *V*_Cs_ has the lowest formation energy among all the vacancy defects, while Sn_I_ has the lowest value among all the antisite or interstitial defects. Under this condition, the Fermi level is pinned at the middle of the bandgap, and CsSnI_3_ behaves as an intrinsic semiconductor. On the other hand, under the Sn‐rich environment, the high concentration of SnI_2_ causes nonradiative recombination, which shortens the carrier lifetime. Conversely, under Sn‐poor conditions, a high concentration of the electron acceptor defect *V*_Sn_ causes a large quantity of the background holes. In this case, the Fermi energy level is pinned to the VBM, and CsSnI_3_ has a strong p‐type conduction behavior. Therefore, a moderate excess of Sn could be used to fabricate CsSnI_3_ solar cells.

In the past several years, intensive research activities have been devoted to suppress the tin vacancies and oxidation by the use of reducing agents, reducing atmospheres as well as mixed 2D/3D perovskites. SnX_2_ (X is Cl, F or I) compounds added to the precursors helped to reduce defects by inhibiting the discoloration of perovskite solution, preventing oxidation, and filling tin vacancies.^[^ ^194^, ^195^, ^196^ ^]^ However, the concentration of these additives, and in particular of SnF_2_, is limited to be 10 mol%, as a higher concentration causes phase segregation, leading to an unfavorable morphology.^[^ ^197^ ^]^ Pyrazine may act as a mediator to restrict the phase separation induced by an excess of SnF_2_.^[^ ^198^ ^]^ When pyrazine and SnF_2_ were used together, they reduced the number of defects more effectively than SnF_2_ only. Marshal et al. compared the effect of SnCl_2_, SnF_2_, and SnI_2_ on CsSnI_3_.^[^ ^199^ ^]^ SnCl_2_‐treated perovskites showed an improved stability due to the interplay of different factors. First, Cl does not easily displace the iodine in the lattice due to size mismatch, therefore SnCl_2_ is pushed toward the surface of the thin film. Second, SnCl_2_ favors the formation of good morphology due to higher solubility in DMF compared to SnF_2_. An excess of SnI_2_ also compensates and suppresses Sn^2+^ vacancies.^[^ ^200^ ^]^ In the trial of reducing the Sn^2+^ oxidation, Gu et al. added metallic Sn powder into the FASnI_3_ precursor solution.^[^ ^201^ ^]^ The authors proposed that Sn powder reduced Sn^4+^ due to the smaller redox potential of Sn^2+^/Sn (−0.13 V) following the redox reaction

(10) $${\text{Sn}}^{4+}+\text{Sn}\to 2{\text{Sn}}^{2+}$$

Song et al. used hydrazine to create a reducing vapor atmosphere in the spin‐coater chamber during the deposition of perovskite film, which decreased the Sn^4+^/Sn^2+^ ratio by more than 20%.^[^ ^202^ ^]^ Here, the hydrazine reduces the Sn^4+^ species by oxidation–reduction as in the following reaction

(11) $$2{\text{Sn}}^{4+}+N_{2}H_{4}\to 2{\text{Sn}}^{2+}+N_{2}+4H^{+}$$

In another work, Li et al. first deposited hydrazinium tin iodide and then put it in a closed vessel with MA gas to form MASnI_3_ film through a cation displacement reaction.^[^ ^203^ ^]^ This approach appeared to be effective to in situ reduce Sn^4+^ species. Kayesh et al. used hydrazinium chloride as an additive, producing a homogenous film of FASnI_3_.^[^ ^204^ ^]^ In addition to the reducing agents mentioned above, agents that contain Lewis base groups, such as P–O, S–O, and C–O, not only reduces Sn vacancies in the perovskite film by the coordination between the Lewis base and the tin vacancy, but also suppress the SnF_2_ segregation phase. For example, the addition of hypophosphorous acid in a solution containing the Sn perovskite precursors changes the color from bright yellow to dark brown as a result of the generation of new compounds via Sn–O–P–O–Sn coordination bond connection.^[^ ^205^ ^]^ More recently, Cao et al. introduced ammonium hypophosphite (AHP) as reducing additive in FASnI_3_ thin films.^[^ ^206^ ^]^ The reducing process follows the equation

(12) $${\text{Sn}}^{4+}+3H_{2}{\text{PO}}_{2}^{-}\to {\text{Sn}}^{2+}+2{\text{HPO}}_{3}^{2-}+{\text{PH}}_{4}^{+}$$

where the oxidized Sn^4+^ is converted back to Sn^2+^. Furthermore, the addition of AHP was shown to suppress the phase separation of SnCl_2_ and to assist the growth of the perovskite grains, similarly to what has been discussed above for pyrazine. Recently, Tai et al. introduced hydroxybenzene sulfonic acid or its salt into the FASnI_3_ perovskite solution along with excess SnCl_2_.^[^ ^207^ ^]^ They compared three different additives, namely, phenolsulfonic acid, 2‐aminophenol‐4‐sulfonic acid, and the potassium salt of hydroquinone sulfonic acid (KHQSA). The hydroxybenzene group (—OH) and the sulfonate group (SO_3_^−^) of these additives act as an oxygen scavenger, by donating hydrogen atoms and electrons. In addition, the SO_3_^−^ group interacted with Sn^2+^ to encapsulate the perovskite grains with a SnCl_2_–additive complex, which improves the stability of FASnI_3_ films upon oxygen exposure. In this sense, KHQSA is the most effective among the three additives. Wang et al. observed a similar trend by introducing phenylhydrazine hydrochloride (PHCl) into FASnI_3_ films to reduce Sn^2+^ oxidation.^[^ ^208^ ^]^ The phenylhydrazine ions (PH^+^) were successfully incorporated into the crystal lattice without forming a 2D structure. The addition of PHCl showed both the effect of trap state passivation but also significantly reduced the content of Sn^4+^, which may also result in self‐repairing capability of the device. As mentioned before, SnX_2_ (X = F, Cl, or Br) tends to create a secondary phase, which inhibits charge transfer across perovskite films. To solve this problem, Wang et al. used as antioxidant gallic acid (GA) together with SnCl_2_.^[^ ^209^ ^]^ GA can limit SnCl_2_ aggregation through the formation of a SnCl_2_–GA complex that envelops the perovskite grain surface, which protects the perovskite from oxidation and suppresses defects. Moreover, GA acts as a secondary antioxidant to defer Sn^2+^ oxidation originating from the reducing hydroxyl groups (—OH) attached to the aromatic ring.

## 6 Charge Recombination and Transport

To better design efficient solar cells, it is important to understand the relevant physical processes upon photocarrier generation. Transport of charge carriers, carrier lifetime, mobility and diffusion length (*L*_D_), doping density, and recombination are all fundamental parameters that describe the photocarrier physics. The carrier dynamics are governed by the following rate equation which considers all possible recombination mechanisms (monomolecular, bimolecular, Auger)

(13) $$\frac{dn}{dt}=\;G-\;k_{1}n-\;k_{2}n^{2}-k_{3}n^{3}$$

where *G* is the charge‐density generation rate and *k*_1_, *k*_2_, and *k*_3_ are the monomolecular (traps), bimolecular (electron–hole), and Auger recombination rate constants, respectively.

### 6.1 Monomolecular Recombination

**Figure 25:**
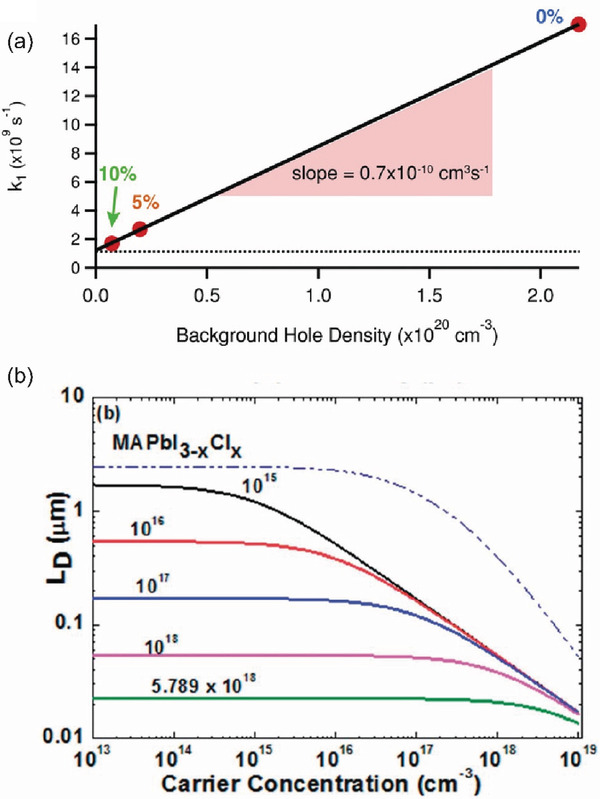
a) Relationship between doping density and the monomolecular charge‐carrier recombination rate, k1. The red markers are experimental data, and the solid black line is a linear fit. b) Diffusion length against the carrier concentration, *n*, for different hole doping levels. (a) Reproduced under the terms of the Creative Common Attribution 4.0 International license.^[^ ^73^ ^]^ Copyright 2021, American Chemical Society. b) Reproduced with permission.^[^ ^212^ ^]^ Copyright 2014, The Royal Society of Chemistry.

Monomolecular recombination involves an electron (hole) in the CB (VB) and the hole (electron) captured by traps within the bandgap (trap‐assisted recombination) or an exciton formed by a coulombically bounded electron–hole pair. However, in 3D tin halide perovskites, the carriers are free charges instead of excitons due to the very small exciton binding energy. Consequently, trap‐assisted recombination is the dominant physical process upon low‐excitation density (photogenerated charge carrier density ≤ 10^15^ cm^−3^). The trap‐assisted recombination rate *k*_1_ is influenced by the density of carriers, the trap capture cross‐section, and the position of the trap level inside the bandgap, which in turn depend on the processing technique and the conditions used to deposit the materials.For MASnI_3_, Johnston and Herz reported a *k*_1_ value of 8 × 10^9^ s^−1^ which is almost three orders of magnitude higher than those of MAPbI_3_.^[^ ^210^ ^]^ This is mainly due to the self‐doping behavior of tin perovskites in presence of tin vacancies and Sn^4+^, inducing a high density of holes. Milot et al. studied the photocarrier decay dynamics in FASnI_3_ films, using time‐resolved THz photoconductivity analysis, and reported a *k*_1_ value of 1.2 × 10^9^ s^−1^.^[^ ^211^ ^]^ FASnI_3_ exhibits a lower monomolecular recombination rate compared to MASnI_3_, which is associated with the lower background of holes. Moreover, the authors observed that the monomolecular charge recombination process is composed of two parts. The first one is a dopant‐mediated part with a radiative recombination constant of 1.1 × 10^8^ s^−1^, and has a linear dependence with the doping density, while the second part that compose the monomolecular charge recombination process is a contribution unrelated to the self‐doping aspect of FASnI_3_. As mentioned previously, the unintentional hole‐doping of FASnI_3_, is much higher with respect to lead halide compounds. Milot et al. showed a linear dependence of the experimental value of *k*_1_ on the doping density in polycrystalline FASnI_3_ films (**Figure** **25**a).^[^ ^73^ ^]^ The doping level was reduced from 2.2 × 10^20^ cm^−3^ without SnF_2_ down to 7.2 × 10^18^ cm^−3^ with 10 mol% SnF_2_, reducing *k*_1_ from 1.7 × 10^10^ s^−1^ down to 1.7 × 10^9^ s^−1^, respectively. For what concerns CsSnI_3_ only few studies are present in literature. In this type of perovskite, the dominant carrier recombination process is the monomolecular recombination due to the elevated intrinsic hole concentration (4 × 10^17^–5 × 10^17^ cm^−3^). Wu et al. extracted a *k*_1_ of 2 × 10^10^ s^−1^ for a polycrystalline CsSnI_3_ film.^[^ ^184^ ^]^

### 6.2 Bimolecular Recombination

Bimolecular charge carrier recombination is the radiative recombination between the free holes and electrons in a semiconductor. Johnston and Herz reported a *k*_2_ value of 1.4 × 10^−9^ cm^3^ s^−1^ for MASnI_3_ which is comparable to that of 3D MAPbI_3_.^[^ ^210^ ^]^ This comparison has to be made with care, because the *k*_2_ parameter exhibits a dependence on the crystallinity and quality of the material and therefore on the deposition process.^[^ ^74^ ^]^ Moreover, the authors observed that the substrate influenced the perovskite crystallization. For example, *k*_2_ increases when the material is infused into a mesoporous metal oxide scaffold compared to a compact film, as the crystallite size is reduced.^[^ ^210^ ^]^ Milot et al. reported a bimolecular recombination rate constant for FASnI_3_ of 2.3 × 10^−10^ cm^3^ s^−1^ comparable to that of lead‐based counterpart, revealing a strong radiative band‐to‐band recombination.^[^ ^211^ ^]^ Xing et al. analyzed CsSnI_3_ compounds determining values for the bimolecular recombination rate constant of 6 (± 2) × 10^−8^ cm^3^ s^−1^ and 1 × 10^−8^ cm^3^ s^−1^ for a CsSnI_3_ solution‐processed films without and with 20% of SnF_2_, respectively.^[^ ^213^ ^]^ The reason behind the large bimolecular recombination rate of Sn‐based perovskites is still unclear and further studies have to be done.

### 6.3 Auger Recombination

Auger recombination involves the recombination of an electron–hole pair, accompanied by energy and momentum transfer to a third electron or hole. Auger recombination strongly depends on the charge‐carrier density and on the electronic band structure as energy and momentum must be conserved. If only two bands (conduction and valence band) are involved, it is difficult to reach this condition. However, the split‐off of the bands emerging from the spin–orbit coupling allows to reach this condition. Johnston and Herz reported some optical‐pump−terahertz‐probe photoconductivity spectroscopy measurements on MAXI_3_ (X = Pb or Sn) films deposited on a mesoporous scaffold of TiO_2_.^[^ ^210^ ^]^ A value of *k*_3_ in the order of 10^−28^ cm^6^ s^−1^ is reported for Pb‐based perovskites, while the value of Auger recombination constant for MASnI_3_ is in the range of 10^−30^ cm^6^ s^−1^. A value for *k*_3_ of 9.3 × 10^−30^ cm^6^ s^−1^ was found also by Milot et al. for FASnI_3_ films.^[^ ^211^ ^]^ Interestingly, the value of *k*_3_ for the Sn compounds is more than an order of magnitude lower with respect to lead‐based perovskites. It has been argued that this lower value with respect to Pb‐based perovskites may be due to the lower spin–orbit coupling with the lighter Sn atoms. This low Auger recombination rate constant suggested that tin perovskites could reach a high radiative efficiency at high charge density, making them suited for lasing applications thanks to the combination of high charge carrier mobilities with low Auger recombination but strong radiative bimolecular recombination rates.

### 6.4 Carrier Transport, Diffusion Length, and Mobility

The carrier diffusion length *L*_D_ is expressed by the following equation

(14) $$L_{D}\left(n\right)=\sqrt{\frac{\mu k_{B}T}{eR\left(n\right)}}=\sqrt{\frac{D}{R_{T}\left(n\right)}}$$

*L*_D_ of tin perovskite films shows a strong dependence on deep acceptor defects, which trap the photogenerated electrons and cause nonradiative recombination with the background holes.^[^ ^212^ ^]^ Noel et al. observed a dependence between the doping density and diffusion length.^[^ ^212^ ^]^ When the hole doping level is 5.789 × 10^18^ cm^−3^ the measured diffusion length was around 20 nm. The authors calculated a diffusion length of 1 µm when the doping density was below 10^15^ cm^−3^ (Figure 25b). *L*_D_ also depends on the device structure used. For example, MASnI_3_ on mesoporous TiO_2_ had a charge carrier *L*_D_ of 30 nm, while on a planar substrate the charge carrier diffusion length of 200 nm was measured.^[^ ^197^, ^212^ ^]^ Fact that can be due to the different crystallinity of the material on different substrates.

Ma et al. compared the diffusion lengths of different MASnI_3_ films, observing that the use of SnF_2_ helps to reduce the background carrier density and improve the diffusion length. Moreover, they showed that using tin powder source with higher purity (SnI_2_ 99.999%) can help to increase the diffusion length. This highlights that the carrier diffusion length depends on the material quality. The authors also used fluorescence quenching measurements to estimate electron and hole diffusion length.^[^ ^197^ ^]^ They observed a value for the electron and hole diffusion constant equal to 1.28 ± 0.73 cm^2^ s^−1^ and 0.59 ± 0.27 cm^2^ s^−1^, respectively, that are higher than those of the lead counterpart (0.036 and 0.022 cm^2^ s^−1^ for electron and hole, respectively). Moreover, they calculated the electron and hole diffusion lengths (*L*_D_^e^ = 279 ± 88 nm and *L*_D_^h^ = 193 ± 46 nm), by using an electron mobility of 2000 cm^2^ V^−1^ s^−1^ and a hole mobility of 300 cm^2^ V^−1^ s^−1^, values in accordance with other studies.^[^ ^28^ ^]^ A charge diffusion length of 35 nm at solar illumination levels was found for FASnI_3_ thin film.^[^ ^211^ ^]^ This value was improved to 265 nm by adding 10% of SnF_2_, reducing the recombination rate of the charge carriers in FASnI_3_ film. CsSnI_3_ polycrystalline thin film was found  to have a high background hole concentration of 9.2 × 10^18^ cm^−3^.^[^ ^184^ ^]^ With this high hole concentration, the authors estimated the minority carrier (electrons) diffusion length for CsSnI_3_ polycrystalline thin filmsto be about 16 nm with a carrier mobility of 1.8 cm^2^ V^−1^ s^−1^. The short diffusion length and low mobility of this highly defective CsSnI_3_ film cause severe charge recombination and result in poor performance when used in solar cells. On the contrary, the electron (minority‐carrier) diffusion length in CsSnI_3_ single crystals (SCs) is around 930 ± 70 nm that is significantly longer than the polycrystalline films because of the lower number of defects.^[^ ^184^ ^]^ Therefore, future research should focus on minimizing the defect density in tin‐based perovskites to improve the charge‐carrier mobility and diffusion length, which are essential parameter to obtain well performing devices.

## 7 Solar Cells

**Figure 26:**
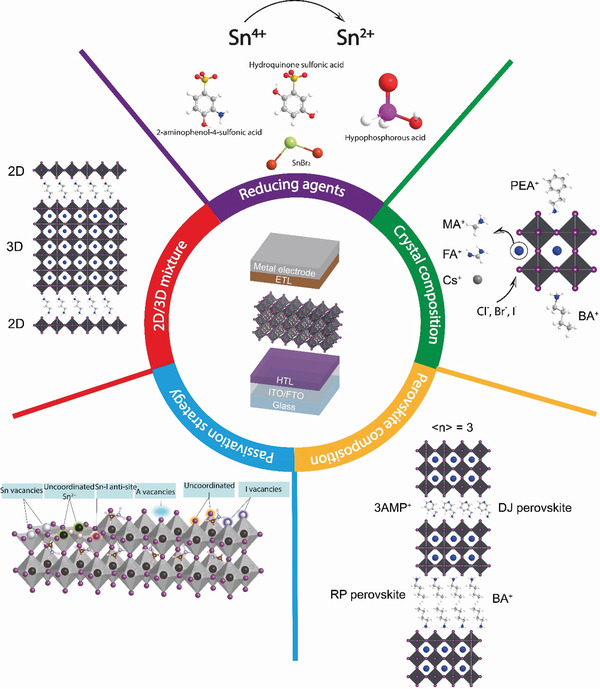
Summary of the different aspects to work on to improve perovskite solar cells performances. Passivation strategy. Adapted with permission.^[^ ^214^ ^]^ Copyright 2019, The Royal Society of Chemistry.

In this paragraph we report the main strategies that have been used to obtain efficient Sn‐based solar cells, the approaches are largely heterogeneous and sometimes difficult to group together in a rational fashion. We have however tried grouping conceptually similar approaches; the schematic in **Figure** **26** has the purpose of summarizing the different strategies.

### 7.1 Device Architecture

The most used device structure for Pb‐based perovskite solar cells is the mesoporous structure, which is composed by a light absorbing layer placed between a compact TiO_2_ layer used as electron transport layer (ETL), a mesoporous TiO_2_ used as scaffold and spiro‐OMeTAD as hole transport layer (HTL). Initially, this architecture was also used for Sn‐based perovskites, however this device structure has obvious drawbacks such as the high‐temperature processing of the TiO_2_ scaffold and the potential damage or oxidation of the Sn perovskite due to the p‐dopant (lithium and cobalt salts) used in the HTL.^[^ ^61^, ^212^ ^]^ To overcome this problem, other device structures were investigated and at the moment the most used is a planar structure, where PEDOT:PSS and fullerenes are used as HTL and ETL, respectively.^[^ ^215^ ^]^ This planar structure gave rise to the highest efficiency and has the advantage of involving only low temperature processing steps.

### 7.2 3D Solar Cells

#### 7.2.1 3D Solar Cells

In this section, a comprehensive overview is given for the evolution of 3D Sn‐based perovskite solar cells. High‐quality thin films are the basis on which high‐efficiency devices are made, therefore many of the solar cells discussed below use techniques for the active layer deposition that have been described in Section 5. Here, more than on the quality of the active layer, we focus on the performance parameters instead. Devices based on MASnI_3_, FASnI_3_, and CsSnI_3_ are separately discussed as material composition greatly affects the efficiency of the solar cells.

##### MASnI_3_

Kanatzidis’ and Snaith's groups were the first to report methylammonium tin halides MASnI_3‐_ *_x_* Br*_x_* (*x* = 0, 1, 2, and 3) and MASnI_3_, respectively, as the active layer in a solar cell. They obtained efficiencies of 5.73% and 6.4%, with MASnI_3‐_ *_x_* Br*_x_* (*x* = 2) and MASnI_3_, respectively.^[^ ^61^, ^212^ ^]^ Kanatzidis’ group fabricated the MASnI_3‐_ *_x_* Br*_x_* solution by mixing in a mortar stoichiometric amounts of MAX and SnX_2_ (X = Br, I).^[^ ^30^ ^]^ They deposited a perovskite film onto a mesoporous TiO_2_ scaffold by spin‐coating the MASnI_3‐_ *_x_* Br*_x_* solution from DMF and observed that an increasing Br content decreases the short‐circuit current (*J*_sc_) due to the blue shift of the absorption onset. Reported stability tests showed that the solar cells maintained 80% of their starting efficiencies after 12 h of being encapsulated and held in a glovebox filled with nitrogen. Snaith's group directly spin‐coated a stoichiometric mixture of SnI_2_ and MAI dissolved in DMF onto a mesoporous TiO_2_ film.^[^ ^61^, ^212^ ^]^ They reported an open‐circuit voltage (*V*_oc_) of 0.88 V, which was only a 0.35 V loss considering the 1.23 eV bandgap of MASnI_3_. This loss in *V*_oc_ value could be justified by the short diffusion length caused by the fast recombination of the charge carriers. Despite the non optimized device structure, deposition methods, and the poor knowledge of tin perovskite chemistry and properties, these two works succeeded to get acceptable efficiencies, revealing the high potential of Sn‐based perovskite as absorbing layer. Nonetheless, the MASnI_3_ solar cells presented poor air long‐term stability compared to the Pb analogues, which was explained by the rapid Sn^2+^ oxidation into the most stable Sn^4+^ state.

To enhance solar cells efficiency, a deep knowledge of tin perovskite chemistry is needed. For example, solvent engineering has been shown to be a suitable method to enhance the efficiency of tin perovskite solar cells by improving crystallization and film quality. As we mentioned in Section 5, DMSO serves as the coordinating solvent for the fabrication of MASnI_3_ thin films.^[^ ^34^ ^]^ Photovoltaic devices fabricated by using this technique showed a high *J*_sc_ of 21.4 mA cm^−2^, even without the use of an HTL. Moreover, MASnI_3_ devices showed one order of magnitude higher photocarrier density compared with MAPbI_3_.

By using a mixture of methanol and 1,4‐dioxane as the solvent for perovskite precursors, Greul et al. demonstrated high‐quality MASnI_3_ and MASnI_3‐_ *_x_* Br*_x_* films.^[^ ^216^ ^]^ These solvents are less toxic than DMF and DMSO, and provide good solubility for the Sn precursors. The proper coordination of dioxane with Sn^2+^ contributed to homogeneous and dense MASnI_3_ films with small crystallites. In order to fabricate smooth MASnI_3_ films with uniform morphology and high coverage, different fabrication methods were employed. For example, solvent bathing method involving toluene and hexane, vacuum‐assisted solution processing, or the addition of reducing vapor agent (hydrazine) were implemented to fabricate high quality MASnI_3_ films with lower defect density. Despite an increment in the device stability, the efficiency was limited to values ≈2% due to the high background hole density of the MASnI_3_ compounds.^[^ ^174^, ^200^, ^216^, ^217^ ^]^

The anion engineering technique has turned to be a potential way to improve the performance of Sn‐based solar cells. Tsai et al. reported a carbon‐based mesoscopic solar cells with MASnIBr_2‐_ *_x_* Cl*_x_* as the active layer.^[^ ^182^ ^]^ The best device was realized with MASnIBr_1.8_Cl_0.2_ perovskite achieving a PCE of 3.1%, where they argued that the Cl had the effect of retarding the charge carrier recombination, and decreasing the charge accumulation. However, the limited efficiency highlights that the understanding of the halides influence on the Sn perovskite active layer is still poor.

##### FASnI_3_

Koh et al. in 2015 employed, for the first time, FASnI_3_ as the active layer in a solar cell by spin‐coating precursors solution on the mesoporous TiO_2_.^[^ ^196^ ^]^ By adding 20 mol% of SnF_2_, the solar cells yielded an overall PCE of 2.10%. Subsequent to these promising results, the scientific community focused on improving the performances of these devices, where additive engineering was one of the main applied methods. In 2016, Gu et al. introduced metallic Sn powder into the FASnI_3_ solution to fabricate solar cells with an inverted structure, where poly(3,4‐ethylenedioxythiophene)–poly(styrenesulfonate) (PEDOT:PSS) and fullerene C_60_ were used as HTL and ETL, respectively.^[^ ^201^ ^]^ The Sn powder leads to a reduction of Sn^4+^ and improved the PCE to 6.75%. The authors also underlined the importance of an optimized device structure and that the purity of the Sn precursor SnI_2_, as Sn^4+^ can be formed during storage. In the same year, Lee et al. used pyrazine additives to obtain a homogeneous dispersion of SnF_2_.^[^ ^198^ ^]^ By virtue of the lower concentration of Sn^4+^ found in the treated films, the solar cells showed a PCE of 4.8%. To further confirm the efficiency of this method, Liao et al. demonstrated high quality, uniform and fully covered FASnI_3_ perovskite films by using SnF_2_ and antisolvent engineering which showed a suitable charge carrier density value of 1 × 10^17^–2 × 10^17^ cm^−3^.^[^ ^219^ ^]^ From these past studies, it is clear that the chemical approach by using additives is an effective method to reduce the Sn^2+^ oxidation.

**Figure 27:**
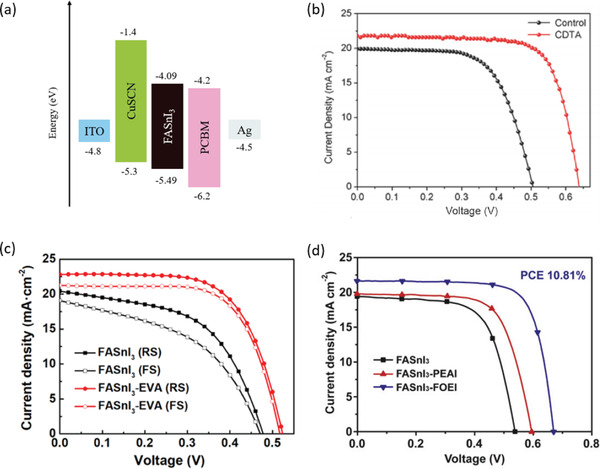
a) Schematic energy diagram of the FASnI_3_ solar cells with structure ITO/CuSCN/FASnI_3_/PCBM/Ag. b) *J*–*V* plots of the best devices based on control and CDTA‐treated samples measured under forward scan. c) *J*–*V* curves of solar cells based on FASnI_3_ and FASnI_3_–EVA perovskite films (reverse and forward scan measurements). d) The *J*–*V* curves of the best devices based on FASnI_3_, FASnI_3_–PEAI, and FASnI_3_–FOEI under 1 sun conditions (the reverse scan). (a) Reproduced with permission.^[^ ^206^ ^]^ Copyright 2019, Royal Society of Chemistry. (b) Reproduced with permission.^[^ ^221^ ^]^ Copyright 2020, Springer Nature. (c) Reproduced with permission.^[^ ^222^ ^]^ Copyright 2020, American Chemical Society. (d) Reproduced with permission.^[^ ^170^ ^]^ Copyright 2020, Elsevier Inc.

In 2019, Cao et al. added ammonium hypophosphite to FASnI_3_ perovskite precursors and used CuSCN as hole transport material due to its suitable energy level (**Figure** **27**a).^[^ ^206^ ^]^ The reference device without additive exhibited poor performances with an average PCE of 2.34% ± 0.71%. By adding 5 mol% of ammonium hypophosphite, the device delivered an average efficiency of 6.25% ± 0.67% and a champion PCE of 7.34% with negligible hysteresis, alongside an outstanding long‐term stability both under N_2_ and ambient conditions. A larger quantity of ammonium hypophosphite led to a decrease of the performance that was ascribed to the presence of pinholes in the rough film. Moreover, this study was one of the first paper which used a different HTL from TiO_2_ or PEDOT:PSS, underling the relevance of using a different HTL to get higher stability and device efficiency. Later on, Li et al. also implemented trihydrazine dihydriodide as an additive which substantially reduced the concentration of Sn^4+^ in the starting solution as well as in the perovskite film, and facilitated the formation of a compact and dense morphology with large crystalline domains.^[^ ^220^ ^]^

With these high‐quality FASnI_3_ films in a planar solar cell structure, they achieved an improved efficiency of 8.48%. Wang et al. introduced a coadditive approach with both gallium acid and an excess of SnCl_2_ that lead to the formation of an amorphous GA–SnCl_2_ complex.^[^ ^209^ ^]^ They demonstrated that the formation of a nanostructure helps to improve the device stability, by creating a self‐encapsulation that avoid moisture to enter in the perovskite structure. Owing to this complex nanostructure, unencapsulated devices exhibited no degradation after 1500 h in N_2_ atmosphere and retained over ≈80% of their initial efficiency after 1000 h in ambient air with a humidity of 20%. When using only SnCl_2_, the large bandgap of SnCl_2_ prohibits the transfer of both charge carriers from FASnI_3_ to the charge transport layers, while the barrier height for electron transfer is dramatically decreased by the GA–SnCl complex. The PSCs with a device structure of ITO/NiO*_x_*/FASnI_3_/PCBM/BCP/Ag showed a significant improvement in the PCE from 3.38% without GA additive to 9.03% for devices with 1 mol% GA. Another example of the self‐encapsulation of the perovskite grains was given by Tai et al.,^[^ ^207^ ^]^ which reported the fabrication of air‐stable FASnI_3_ solar cells by introducing hydroxybenzene sulfonic acid as antioxidant additive into the perovskite precursors solution alongside the use of SnCl_2_. Similarly to GA, the authors argued that the additive strategy would lead to the in situ encapsulation of the FASnI_3_ grain by a SnCl_2_–additive complex layer, which could be highly advantageous for the stability of the inner perovskite. Furthermore, the lower density of Sn^4+^ in the treated perovskite film led to an improvement in the solar cells, which had an average PCE of 5.73% ± 0.63% and a maximum efficiency of 6.76% in an ITO/NiO*_x_*/FASnI_3_/PCBM/Ag device structure.

Another aspect to be considered to improve tin‐based perovskite solar cells is the fast crystallization process which leads to low quality perovskite films. Recently, many research groups implemented π‐conjugated Lewis‐base molecules to slow down the crystallization process of the Sn‐based perovskite or to suppress the Sn^2+^ oxidation. For example, Wu et al. treated FASnI_3_ films with 2‐cyano‐3‐[5‐[4‐(diphenylamino)phenyl]‐2‐thienyl]‐propenoic acid (CDTA).^[^ ^221^ ^]^ They reported that the aromatic π‐conjugated system combined with the various electron‐donating functional groups of CDTA control the interaction with the SnI_2_ precursor. This strong electron‐donating behavior contributes to a more stable Lewis adduct during the nucleation process. Consequently, the treated FASnI_3_ film showed a pinhole‐free and homogeneous morphology, accompanied by a suppression of the in‐plane and out‐of‐plane rotation of [SnI_6_]^4−^. Furthermore, the hydrophobic CDTA molecules formed mainly on the surface of the perovskite film, which is beneficial for retarding the penetration of moisture and oxygen into the FASnI_3_ thin film.^[^ ^223^ ^]^ As a result, the devices with a ITO/PEDOT:PSS/FASnI_3_/C_60_/BCP/Ag structure showed a high PCE of 10.32% with respect to the pristine PCE of 6.70% (Figure 27b), and also exhibited a good stability under continuous light soaking at the maximum power point for 1000 h. Apart from Lewis base molecules, Meng et al. added poly(vinyl alcohol) (PVA) inside the FASnI_3_ solution.^[^ ^224^ ^]^ PVA is a polymer characterized by a high density of hydroxyl groups, which can form directional hydrogen bonding interactions involving the polar hydrogen group O^δ−^–H^δ+^ and the electronegative iodide ion I^δ−^. The strong O—H—I^−^ hydrogen bonding interactions between PVA and FASnI_3_ suppresses the iodine migration, with the resulting increase of the long‐term stability of devices. In addition, an improvement with respect to the pristine device was observed with a maximum PCE of 8.9% attributed to the reduced number of defects. In 2020, Wang et al. demonstrated the efficacy of phenylhydrazine hydrochloride molecules to reduce the Sn^4+^ presence in FASnI_3_ films and reduce the degradation of the active layer.^[^ ^208^ ^]^ Furthermore, the authors fabricated highly performing solar cells with a PCE of 11.4%, underling the effectiveness of the additive method.

Passivating defects and trap states formed at the surface and grain‐boundary is a widely used method to increase the performances of solar cells. The high solubility of Sn‐based perovskites in many polar solvents makes it difficult to apply a passivation protocol involving a solution‐based treatment. In literature, only a few cases of this method can be found. For example, Kamarudin et al. demonstrated a post‐treatment of tin perovskite film by bidentate amine, ethane‐1,2‐diamine (edamine), to passivate the surface defects in order to increase the *V*_oc_.^[^ ^225^ ^]^ An optimal concentration of 0.05 × 10^−3^ m of edamine converted the unreacted SnI_2_ into a perovskite phase and improved the active layer surface morphology and grain size. Furthermore, the amine group in the ethane‐1,2‐diamine molecules helped to stabilize the undercoordinated Sn in the perovskite by donating free electron pairs. As a result of these physical improvements, the solar cells reached a PCE of 9.37%. Interestingly, the edamine‐passivated devices showed a higher efficiency of 10.18% after 7 days storage in a N_2_ atmosphere. Liu et al. reported a pretreatment method for fabricating FASnI_3_ film by spin‐coating a solution of n‐propylammonium iodide (PAI) in a mixed solvent of chloroform and DMSO.^[^ ^226^ ^]^ Using DMSO as solvent in a surface passivation method is arduous due to the high solubility of tin perovskite compounds in dimethyl sulfoxide and the difficulty to remove this solvent by thermal annealing. These problems may first of all affect the device reproducibility. However, the authors claimed that DMSO can dissolve the crystals at the surface, creating a liquid phase environment. In these conditions, PAI cations aggregated around the newly formed FASnI_3_ nucleates to form a template, which promotes the perovskite to grow along the (100) plane. Here, the PA cations do not enter in the crystal lattice nor form a 2D structure. This method produced films with a much lower roughness of 3.37 nm compared to the 22.49 nm of the pristine perovskite, and a defect density of one order of magnitude lower. Consequently, solar cells exhibit significantly improved PCE of 11.73% compared with the reference efficiency of 7.93%. The encapsulated devices maintained 95% of their initial efficiency after 1000 h of operation in the N_2_ atmosphere.

Cao et al. reported a strategy where a seed growth method is used to regulate the crystal growth.^[^ ^227^ ^]^ First, a layer of perovskite film was deposited through a typical antisolvent method without the thermal annealing step. Subsequently, one more deposition process was repeated using the same precursor solution and method. AFM images showed that the residual perovskite materials from the precoated procedure were not agglomerated, which was hypothesized to act as a seed layer for the subsequent growth of perovskite films with bigger grains (≈342 nm). The seed growth method also induced a preferential orientation of the (100) plane. The control device exhibited a champion PCE of 5.37% with a *V*_oc_ of 0.47 V and an FF of 57.3%, while, the solar cell fabricated by the seed growth method showed an improved PCE of 7.32% along with a *V*_oc_ of 0.49 V, and an FF of 66.2%. The enhanced *V*_oc_ and FF were attributed to a reduced defect density of the larger grains. It is reasonable to wonder about the reproducibility of this method, as it seems difficult to control the process of partial dissolution of the perovskite layer.

Liu at al. implemented poly(ethylene‐*co*‐vinyl acetate) (EVA) as a self‐sealing polymer, which interacts with SnI_2_ to form a Lewis acid–base complex.^[^ ^222^ ^]^ This new system acts as a template to slow down the crystallization rate and regulate the growth orientation of FASnI_3_ thin films. The EVA treatment resulted in a champion PCE value of 7.72% with a weakened hysteresis effect (Figure 27c). Furthermore, the EVA treatment effectively prevents moisture and oxygen from permeating into the grain boundaries of the perovskite layer.

As we discussed in Section 5, the controlled surface crystallization strategy using FOEI molecules was implemented to obtain higher quality FASnI_3_ perovskite films.^[^ ^170^ ^]^ These organic molecules migrate to the solution–air interface to reduce the perovskite surface energy during the spin‐coating process and form a very thin layer, which serves as a template for nucleation and growth of highly oriented films. This surface‐controlled growth resulted in smoother films with a root mean square roughness of 8.95 nm versus the 25.97 nm of the pristine film. The best solar cell with FOEI showed a PCE of 10.81% (Figure 27d). More impressively, these devices almost maintained their initial efficiency after 500 h of light soaking under AM1.5G due to defects passivation by the ammonium cation and the effective barrier against water by the hydrophobic fluorinated cation.

Another approach to improve the solar cell efficiency is to work on the perovskite deposition technique. In 2017, Zhu et al. implemented the first sequential deposition route for FASnI_3_ thin films.^[^ ^228^ ^]^ The first‐step consisted of a SnY_2_ (Y = I^−^, F^−^) solution with trimethylamine (TMA) to form SnY_2_–TMA complexes, followed by dip coating in an FAI precursor solution to convert the film into FASnI_3_. The SnY_2_–TMA complexes retard the fast reaction between SnI_2_ and FAI. The weaker affinity of TMA with SnI_2_ than FA^+^ ions facilitates the intramolecular exchange with FA^+^ ions and allows the formation of dense FASnI_3_ films with less pinholes. The n–i–p structure shows a best PCE of 4.34% while the p–i–n structure displays a best PCE of 7.09%, both structures showing a higher performance than the control devices.

Liu et al. implemented a hot antisolvent treatment and a vapor method to improve the PCE of solar cells from 5.7% to a maximum PCE of 7.2%.^[^ ^173^ ^]^ In order to improve the oxygen stability of Sn‐based perovskite solar cells, He et al. used 4‐fluorobenzohydrazide (FBH) as additive in the antisolvent to form a carbonylate antioxidant capping layer on the FASnI_3_ perovskite surface.^[^ ^229^ ^]^ This method effectively reduced the Sn^4+^ generation in the perovskite film during the entire fabrication process carried out at different oxygen concentrations. As a consequence, the solar cells fabricated in this way achieved a champion efficiency of 9.47% at 0.1 ppm of oxygen and 9.03% at 100 ppm. Furthermore, this FBH‐based device when fully encapsulated maintained over 93% of its initial PCE after 600 h under continuous light soaking.

##### CsSnI_3_

CsSnI_3_ perovskite solar cells achieved much lower PCE than the aforementioned tin perovskite solar cells, mainly due to a fast degradation from the photoactive B‐γ‐CsSnI_3_ to the yellow polymorph structure, which subsequently transforms to the photo inactive Cs_2_SnI_6_ due to a rapidly self‐doping reaction from Sn^2+^ to Sn^4+^. However, these all‐inorganic perovskites still attract significant attention because of their higher stability. Kanatzidis and co‐workers were the first to implement CsSnI_3_ as an HTL in a solid‐state dye‐sensitized solar cell, which showed a promising PCE of 3.72%.^[^ ^194^ ^]^ They further improved the efficiency of the dye‐sensitized solar cell to 9.28% by doping the CsSnI_3_ perovskite films with 5% SnF_2_. In 2012, Chen et al. pioneered using CsSnI_3_ as active layer in a Schottky type solar cell, where the CsSnI_3_ active layer was placed between ITO and gold/ITO contact.^[^ ^230^ ^]^ A low PCE of 0.9% and FF of 22% were attributed to a combined effect of the low shunt resistance and the large series resistance. Starting from these pioneering works, various solutions were applied to improve CsSnI_3_‐based perovskite solar cells. For example, as additive engineering was used in MASnI_3_ and FASnI_3_ perovskite, thus several groups started to analyze the impact of the previously cited additives on the CsSnI_3_ compounds. Kumar et al. used the SnF_2_ additive to increase the performance of solar cell by using a one‐step spin‐coating method.^[^ ^195^ ^]^ The champion device with 20% of SnF_2_ exhibited a PCE of 2.02%, and relatively good stability with a negligible variation in performance after 250 h of storage in a glovebox. Heo et al. investigated the impact of the SnX_2_ (X = F, Cl, and Br) additives on the stability and efficiencies of CsSnI_3_ perovskite solar cells.^[^ ^231^ ^]^ The solar cells fabricated using SnF_2_ exhibited a PCE of 3.40%, while the devices with SnCl_2_ and SnBr_2_ exhibited PCEs up to 3.90% and 4.30%, respectively. Interestingly, the PCEs of devices using SnF_2_ and SnCl_2_ dropped by more than 50% after 10 h of storage in the N_2_ atmosphere, while the solar cell with SnBr_2_ still maintained 97.9% of its initial efficiency. Supported by DFT calculations, the authors explained this improved stability for the SnBr_2_ treated devices by the fact that SnBr_2_ prevents the phase transformation of black CsSnI_3_ perovskite to the yellow phase and passivated surface defects. Marshall et al. found that an excess of SnI_2_ in the precursor solution reduced the defect density of CsSnI_3_ films.^[^ ^232^ ^]^ Furthermore, replacing the C_60_ ETL by indene‐C_60_‐bis‐adduct (IC_60_BA) further improved the PCE of the solar cells from 1.5% to 2.76%. The better energy alignment of IC_60_BA resulted in an increased *V*_OC_ from 0.28 to 0.55 V. Song et al. obtained a PCE of 4.81% by using an excess of SnI_2_ and hydrazine as reducing agent, simultaneously.^[^ ^200^ ^]^ The same authors also found that adding piperazine into the precursor solution suppresses the formation of the detrimental Sn^4+^, which reduces the conductivity of CsSnI_3_ and eliminates the shorting paths in solar cells.^[^ ^233^ ^]^ Consequently, CsSnI_3_‐based perovskite solar cells showed a PCE of 3.83%.

Cations and ions engineering also had a function in boosting the CsSnI_3_‐based perovskite solar cells. Counterintuitively, a partial substitution of Sn with Ge was reported to give rise to more stable CsSn_0.5_Ge_0.5_I_3_ than CsSnI_3_ and CsGeI_3_ when subjected to air and light.^[^ ^234^ ^]^ This was explained as due to the formation of a thin layer of ≈5 nm of stable Sn‐doped GeO_2_ on the film surface, which not only suppressed the formation of volatile and unstable Ge suboxides but also enhances the air stability of GeO_2_. The solar cells with CsSn_0.5_Ge_0.5_I_3_ light harvesting layer fabricated in air showed a higher PCE of 7.11% than the 3.72% of the device fabricated in N_2_ atmosphere, indicating the role of GeO_2_ in the protection of the active layer. Moreover, the authors demonstrated that the addition of GeI_2_ boosts the device efficiency only if a limited amount of O_2_ and H_2_O (>0.1 ppm) is present during the film deposition, which trigger the GeO_2_ formation.

Incorporation of Br^−^ ions in CsSnI_3_ was demonstrated to be a valid method to increase the *V*_oc_ of solar cells by increasing the material's bandgap.^[^ ^235^ ^]^ However, the pure bromide‐based compound, CsSnBr_3_, showed an additional phase of CsSn_2_Br_5_ in the film impeding to achieve the expected *V*_oc_. Gupta et al. reported a promising perovskite solar cell by using CsSnBr_3_ as the active layer.^[^ ^236^ ^]^ The highest PCE achieved was 2.1% with the addition of 20 mol% of SnF_2_. The addition of SnF_2_ decreased the VBM of the compound, which in turn increased the *V*_oc_ since a better energy alignment with the HTL was obtained.

One important lesson learned from Pb‐based PSCs is that optimization of perovskite fabrication techniques can effectively increase the device performance. Therefore, more fabrication methods needed to be developed to obtain high‐quality CsSnI_3_ films. Li et al. applied the VASP method to fabricate CsSnI_3_ perovskite films as described in section crystallization and film formation.^[^ ^180^ ^]^ The fabricated solar cells exhibited low efficiency (≈3%), revealing the difficulties of getting high quality evaporated films.^[^ ^181^, ^237^ ^]^

Another way to improve the efficiency of this type of devices is to fabricate lower dimensional perovskites. These methods are difficult to implement due to the instability and the difficulty to fabricate perovskite nanostructures. For this reason, there are only few studies present in literature. For example, Chen et al. reported about the preparation of CsSnX_3_ (X = Cl, Br, and I) quantum rods with uniform diameters to improve solar cells efficiency.^[^ ^183^ ^]^ The CsSnX_3_ films were spin‐coated starting from a rod dispersion and followed by a thermal annealing at 110 °C. The best solar cell with the structure of ITO/TiO_2_/CsSnI_3_/spiro‐OMeTAD/Au reached an efficiency of 12.96%. However, this high efficiency has not been reproduced by others, and the use of TiO_2_ and spiro‐OMeTAD in the structure that tends to react with Sn‐based materials casts further doubt on the reported performance. Wang et al. reported a simple process to synthesize lower dimensionality CsSnI_3_ perovskite structures.^[^ ^238^ ^]^ They fabricated CsSnI_3_ perovskite quantum dots by a one‐pot synthesis using the addition of an antioxidant solvent additive, namely, triphenyl phosphite (TPPi). They observed that the microcrystalline domains of 1 µm decreased to 100 nm upon the addition of TPPi. The solar cells fabricated with the device structure of ITP/PEDOT:PSS/CsSnI_3_/PCBM/Ag exhibit an improvement when the nanostructures are implemented. The nonquantum dot devices with 4 vol% TPPi exhibited a PCE of 1.20%. Instead, for the quantum dots‐based devices, PCE was enhanced up to 5.03%. Moreover, the quantum dot‐based devices showed good stability, retaining 72% of the initial efficiency after 30 days of storage in a N_2_ glovebox.

### 7.3 Mixed Cation Solar Cells

Mixing the cation has been shown as an effective method to improve the efficiency of lead perovskite solar cells. In the case of the tin‐based counterparts, many research groups have also investigated the effect of the mixed cations, among the most used are: MA/FA, ethylenediammonium (*en*), hydrazinium (HA^+^), dimethylammonium (DMA^+^), 2‐hydroxyethylammonium (HEA^+^), Cs^+^, Rb^+^, Na^+^, K^+^, Cs^+^, BA^+^, and EA^+^. Some of these cations gave rise to hollow or 2D/3D structure, while others maintained the 3D structure by successful modulation of the crystal tolerance factor, strain, and structure of perovskite films. Ferrara et al. first demonstrated FA_1‐_ *_x_* MA*_x_* SnBr_3_ thin films.^[^ ^239^ ^]^ Later on, Zhao et al. partially substituted MA with FA in FA*_x_* (MA)_1‐_ *_x_* SnI_3_, leading to increased lattice parameters, improved film morphology, and reduced carrier recombination.^[^ ^240^ ^]^ These improvements led to a champion device for *x* = 0.75 with a PCE of 8.12%. Liu et al. further improved the efficiency of FA_0.75_MA_0.25_SnI_3_ perovskite solar cells by studying the effect of the antisolvents diethyl ether, toluene, and chlorobenzene.^[^ ^172^ ^]^ They observed that using the latter, the film morphology was improved and the charge carrier recombination was reduced. The champion device exhibited a PCE of 9.06%. Tsarev et al. partially substituted MA with HA in MASnI_3_ in order to enhance the operational stability of the solar cells.^[^ ^241^ ^]^ The authors observed that by introducing 20% of HA enhanced the photostability, with a negligible degradation after 100 h of light exposure. Pisanu et al. used DMA in MASnBr_3_ to improve the air stability, which they propose to come from the hydrogen bonding network.^[^ ^242^ ^]^ Ma et al. implemented hexamethylenediamine diiodide (HDAI_2_) as a passivation agent in FASnI_3_ perovskite solar cells.^[^ ^243^ ^]^ They selected this cation due to the two ammonium groups of HDA^2+^ that can form hydrogen bonds with the [SnI_6_]^4−^ octahedra to neutralize defects. Another advantage of this cation is that it links with the amino groups to the inorganic octahedra to form the 2D layered perovskite. Surprisingly, the XRD and GIWAXS measurement did not reveal any 2D layered perovskite, possibly due to the low concentration of HDA^2+^ cations used. The champion device with HDAI_2_ passivating agent exhibited a PCE of 7.6% with a higher FF and *V*_oc_ than the neat FASnI_3_ devices. Yu et al. obtained a MA_0.25_FA_0.75_SnI_2.75_Br_0.25_ film by mixing MABr, FAI, and SnI_2_.^[^ ^244^ ^]^ Here, the MA cations were responsible for the higher oriented crystallization with the preferential facet of (001), while the Br^−^ anions played an important role in bandgap modulation. The champion device showed a PCE of 9.31% with an improvement in FF and *V*_oc_ with respect to the pristine solar cell. Moreover, this device under 1 sun was more stable, and it retained 80% of its initial efficiency after 300 h versus 120 h for the pristine device.

Inspired by successful improvement on Pb‐based solar cells, some research groups demonstrated that Cs cations can also be incorporated in pure Sn‐based devices to increase their performance. For example, Gao et al. incorporated a small quantity of CsI in FASnI_3_ to increase the thermodynamic stability of the perovskite film.^[^ ^245^ ^]^ The device with 8% of CsI had an efficiency of 6.08%, which retained 90% of its initial PCE after storage in N_2_ for 2000 h. Bernasconi et al. partially substituted Cs with Rb, obtaining an inorganic Cs_1‐_ *_x_* Rb*_x_* SnBr_3_ perovskite.^[^ ^246^ ^]^ With 8% of RbCl they fabricated a champion device with a PCE of 5.89%, due to a better crystal quality and reduced Sn^2+^ oxidation. Hayase's group studied the relationship between the lattice strain and photovoltaic performances.^[^ ^247^ ^]^ They analyzed the effect of Na^+^, K^+^, Cs^+^, BA^+^, and EA^+^ in Q_0.1_(FA_0.75_MA_0.25_)_0.9_SnI_3_, where Q are the mentioned cations. They found that the lowest lattice strain value is obtained by using 0.1% of EA, which is important to increase the carrier mobility. This decreased lattice strain was directly correlated with the champion device showing an efficiency of 5.41%. In a follow‐up work, they included EAI in a Ge‐doped tin‐based perovskite, where the final composition of (FA_1‐_ *_x_* EA*_x_*)_0.98_EDA_0.01_Ge_0.05_SnI_3_ resulted in the previous record efficiency for Sn‐based perovskites of 13.24%.^[^ ^248^ ^]^ Here, EA was crucial to tune the energy levels for efficient charge extraction by tuning the lattice distortion. Liu et al. fabricated an efficient and stable tin perovskite solar cell based an amorphous‐polycrystalline structure composed of a tin triple‐halide amorphous layer and CsFASnI_3_ polycrystals, confirming the importance of forming a protecting layer for the perovskite film.^[^ ^249^ ^]^ Through XPS analysis and time‐of‐flight secondary ion mass spectroscopy measurements, these authors observed that the amorphous structure is composed of the tin triple halide of F, Cl, and I, and it is located only on the top of the perovskite film. This structure blocks the outside moisture, oxygen, and the ion diffusion inside the device. A champion device with a PCE of 10.4% was reported. More impressively, the solar cell also retained over 95% of its initial PCE after 1000 h of operation at the maximum power point under simulated sunlight of AM1.5G.

### 7.4 “Hollow” Tin Perovskite Solar Cells

As we analyzed in Section 3.1.1, the large cations, such as *en*, introduced a lot of SnI_2_ vacancies in the 3D structure, creating hollow perovskite. The discontinuity in the crystal structure opened a new way to fabricate Sn‐based perovskite solar cells with enhanced stability and performance. In fact, these hollow perovskite films exhibit superior morphology and simultaneously reduced background carrier density and increased carrier lifetime. Ke et al. reported improved performance of the solar cells when the hollow perovskite film was fabricated with 15% *en*.^[^ ^76^ ^]^ The pristine device exhibited a low PCE of 0.17% and a considerable leakage current. This was attributed to the poor surface coverage of perovskite film which created contacts between HTL and ETL. By contrast, the devices fabricated with 15% of *en* exhibited a PCE of 5.49%. The authors also used a reducing vapor atmosphere based on hydrazine during the fabrication of the hollow perovskite films, achieving a higher PCE of 6.63%. Furthermore, the unencapsulated and hydrazine treated MASnI_3_ devices showed better stability under constant AM1.5G illumination in air at room temperature, retaining ≈60% of its initial condition after 10 min. In order to prove the effectiveness of *en*, the authors fabricated a CsSnI_3_‐based device in the same fashion, achieving a PCE of 3.79% with respect to 0.45% for the pristine device. For FASnI_3_‐based solar cells an optimum of 10% of *en* cation resulted in a maximum PCE of 7.14%.^[^ ^76^ ^]^ The unencapsulated *en*‐FASnI_3_ perovskite films showed better stability over 6 h of air exposure, exhibiting no serious degradation. In addition, the thermal stability of the devices was improved, and the treated films retained their black phase after a thermal annealing of 40 min at 100 °C in air. On the contrary, the pristine films decomposed and became transparent. Under constant AM1.5G illumination in air at room temperature, the pristine devices degraded rapidly with the efficiency dropping from 1.28% to 0% after 20 min. On the other hand, the unencapsulated *en‐*FASnI_3_ device retained 50% of its initial efficiency under the same conditions. In 2018, the same group reported hollow FASnI_3_ perovskite incorporating another two diammonium cations of propylenediammonium and trimethylenediammonium.^[^ ^78^ ^]^ They reported an improvement in efficiency for the treated devices from 2.53% to 5.85%, and 5.53% for PN‐FASnI_3_ and TN‐FASnI_3_, respectively. The authors attributed this improvement to a lower trap density and a lower dark current that suggested improved charge transport and decreased recombination losses.

### 7.5 2D/3D Tin Perovskite Solar Cells

After the pioneering works of Kanatzidis and Snaith in 2014, many research groups struggled with improving the PCE of the 3D tin PSCs in the successive years. A breakthrough came in 2018 by incorporating a small amount of large organic cations into the precursor solution of 3D perovskite to form 2D/3D perovskites. Shao et al. first reported a 2D/3D tin‐based PSCs with a PCE of 9% by partially substituting FA with a tiny amount of PEA. The authors argued that the higher degree of crystallinity was the main reason for the better performance of 2D/3D devices. The improvement in the *V*_oc_ was related to the suppression of Sn vacancies due to an extended ordering and packaging of the crystal planes which improves the robustness of the perovskite structure. Moreover, the 2D/3D device maintained 59% of its initial PCE after exposure to air for 76 h thanks to the hydrophobic nature of PEA. Following the aforementioned work, the authors systematically investigated the impact of the stoichiometry of the 2D/3D (PEA_0.08_FA*_x_* SnI_3_, where *x* refers to the concentration of the FA cation in the case of fixed PEA concentration) tin perovskite on solar cell performance.^[^ ^250^ ^]^ They found that the concentration of the small FA^+^ cation influences the quantity of the 2D component and the crystallinity of the 3D component. 2D/3D films containing excess FA (*x* > 1.0) mainly consist of poorly crystalline and randomly oriented 3D phases, with much higher trap density compared to the reference film. The corresponding solar cells therefore suffers from severe trap‐assisted charge recombination and deliver a poor PCE of <1%. FA‐deficient 2D/3D films (*x* ≤ 0.8) form highly crystalline and oriented 3D grains, and at the same time a large quantity of 2D (*n* ≤ 2) phases throughout the entire film. Consequently, these parallel oriented 2D phases hinder charge transport in FA‐deficient solar cells, resulting in severe recombination of free holes and electrons and inferior PCE compared to the reference devices. Furthermore, Shao et al. first demonstrated that EA enhances the crystallinity and orientation of the 2D/3D film, which reduces the trap density by a factor of two and enables efficient tin perovskite solar cells employing cheaper SnI_2_ with lower purity.^[^ ^251^ ^]^

Later, Ning et al. reported a hierarchical 2D‐quasi 2D–3D structure using NH_4_SCN as an additive to regulate the film structure.^[^ ^191^ ^]^ With this approach they achieved a high PCE of 9.41% with an increase in FF and photocurrent, due to an enhancement of the carrier mobility and a reduction of the background carrier density. Moreover, it also remarkably improves the ambient stability of this device due to the growth of a very thin layer of 2D PEA_2_SnI_4_ on top of the 3D FASnI_3_ which retained a remarkable 90% of its initial PCE after 600 h of storage in air. Later on, Jiang et al. replaced PCBM and NiO_x_ with ICBA and PEDOT:PSS in the device structure, leading to a record V_OC_ value of 0.94 V and a PCE of 12.4%.^[^ ^252^ ^]^ Furthermore, the encapsulated device showed a high shelf stability by maintaining 90% of its initial efficiency for over 3800 h of storage in N_2_ atmosphere. This study was one of the first work in which the authors used a different ETL from C_60_, getting a high *V*_OC_ value. These results underline the importance of selection of suitable ETLs and HTLs for tin perovskite materials, which is still an under investigated strategy.

Chen et al. reported a 2D/3D structure formed by spin‐coating a thin PEABr film and a FASnI_3_ layer sequentially, where PEA partially substituted FA in the 3D perovskite system, hence forming PEA_2_SnBr_4_ layers at the PEDOT:PSS interface.^[^ ^253^ ^]^ The low‐dimensional perovskite layer enabled the uniform growth of FASnI_3_ film, reducing the trap state density. These improvements enabled solar cells with a PCE of 7.05% alongside a stabilized power output and a negligible hysteresis. PEA was also implemented with a mixed cation tin‐based perovskite material. For example, Rath et al. used a triple cation MA_0.75_FA_0.15_PEA_0.1_SnI_3_ to develop a 2D/3D perovskite material.^[^ ^254^ ^]^ In their study, they deposited the perovskite film using a double antisolvent process and a preheated substrate at 70 °C to obtain a homogeneous and pinhole‐free film. The device showed an astonishing stability in the N_2_ atmosphere, retaining 87% of its initial efficiency after 5000 h. In addition to NH_4_SCN, FASCN is used as an additive in 2D/3D perovskite films to improve the crystallinity in the out‐of‐plane direction and to protect the perovskite film from oxidation.^[^ ^35^ ^]^ The PSC using FASCN additive exhibited an improved efficiency of 8.17% with respect to 5.74% for the pristine device without additive. In addition, the device retained 90% of its initial efficiency after 1000 h of storage in a nitrogen atmosphere, probably due to the improved crystallinity of 2D/3D perovskite film.

In addition to PEA, other long organic cations were also implemented to realize a 2D/3D perovskite structure. For example, Jokar et al. implemented ethylene diammonium diiodide and butylammonium iodide to passivate the surface defect states and reduce Sn^2+^ oxidation.^[^ ^163^ ^]^ These two cations led to an improvement of the solar cell efficiency from 4.0% for the 3D to 7.4% for the 2D/3D device, which continuously increased to 8.9% when the device was stored in the N_2_ atmosphere for 1400 h. Jokar et al. also combined guanidinium and ethylene diammonium cations into the FASnI_3_ precursors and reported a PCE of 8.5%, which reached a value of 9.6% after being stored in nitrogen atmosphere for 2000 h.^[^ ^255^ ^]^ Kayesh et al. analyzed the impact of 5‐ammonium valeric acid (5‐AVAI) on FASnI_3_ perovskite solar cells.^[^ ^162^ ^]^ They observed that the hydrogen bonds of 5‐AVAI with the SnI_6_^4−^ octahedra improved the crystal growth of the perovskite film and resulted in a lower Sn^4+^ content. Moreover, they obtained a PCE of 7.0% in a device with an area of 0.25 cm^2^. Remarkably, the devices exhibited a negligible degradation either under 1 sun continuous illumination at maximum power point tracking for 100 h or after a prolonged air exposure. The 5‐AVAI molecules placed at grain boundaries prevented oxygen or moisture from entering the perovskite bulk. Ran et al. introduced the conjugated 3‐phenyl‐2‐propen‐1‐ammonium (PPA) in the FASnI_3_ perovskite.^[^ ^256^ ^]^ PPA not only passivated the FA vacancies at the surface of the film, but also decreased the crystallization rate, leading to a larger grain size. Additionally, perovskite films had a preferential crystallization toward (100) direction. The champion device fabricated with 15% of PPA reached a PCE of 9.61%.

Chen et al. added a bulky divalent organic cation, 4‐(amino‐(aminomethyl)‐piperidinium (4‐AMP) into FASnI_3_ to fabricate solar cells.^[^ ^257^ ^]^ A 15 mol% of 4‐AMP resulted in a champion device with a PCE of 10.9%. Interestingly, this concentration was not enough to create detectable low‐dimensional phases. Hence, the authors argued that the 4‐AMP cations interact with surfaces and grain boundaries of the perovskite film.

Recently, Jokar et al. developed a sequential method to grow 2D/3D perovskites.^[^ ^258^ ^]^ The FA/GA cocation 3D film (E1G20) was deposited using a one‐step procedure. The 2D layer was formed by spin‐coating a solution of a long organic cation and hexafluoro‐2‐propanol (HFP) as solvent, of which the latter formed strong interaction with the former bulky ammonium cations and slows down its reaction with the 3D perovskite film, producing a 2D phase or quasi‐2D phase. The thin 2D perovskite film passivated the boundaries of the 3D grains and protected the active layer from moisture penetration, enhancing the PL intensity and charge carrier lifetime. The best device achieved a PCE of 10.6% using anilinium as passivation cation, and the unencapsulated device was stable in ambient conditions (RH 40%, *T* = 20 °C) for over 150 h.

Adding long fluorinated cations into the perovskite structure is a proper method to improve device stability and efficiency. The substitution of hydrogen atoms by highly electronegative fluorine atoms can induce higher dipole moment and strong polarity of the perovskite crystal lattice.^[^ ^259^ ^]^ The improved polarization helps the charge separation and transport in the device. Moreover, the fluorinated cations can create stronger van der Waals interaction or hydrogen bonding, improving the device stability. This method led to the actual record efficiency. Yu et al. implemented 4‐fluoro‐phenethylammonium cations (FPEABr) in FASnI_3_ perovskite material, creating a microstructure where 2D phase embraces the 3D grains located at the surface and grain boundaries.^[^ ^31^ ^]^ This microstructure affectively reduced the Sn^2+^ oxidation and the defect density, leading to a PCE enhancement from 9.38% to 14.81% (using ICBA as ETL). Moreover, due to the higher hydrophobicity of the fluorine atoms, the treated device exhibited higher thermal and ambient stability (75% of humidity).

### 7.6 Low‐Dimensional (Quasi‐2D) Perovskite Solar Cells

As mentioned earlier, layered tin perovskites are very promising light harvesting materials for solar cells due to their superior structural stability over their 3D counterpart. Depending on the organic cation employed, in particular when hydrophobic, a superior stability to heat, moisture, or oxygen is seen. Among the layered tin perovskite family, pure 2D tin perovskite materials (*n* = 1), are not suitable light harvesting materials due to the high exciton binding energy and wide bandgap, which harm the free charge generation capability needed in a solar cell. The quasi‐2D tin perovskite materials with *n* = 3, 4, and 5 exhibit weaker quantum confinement effect compared to the 2D materials and have been frequently investigated in solar cells, as they benefits from the smaller exciton binding energy, narrower bandgap, and the good structural stability of these quasi‐2D materials. Despite the aforementioned advantages, quantum and dielectric confinement effects of quasi‐2D tin perovskite materials still play an important role in determining the charge generation, transport, and recombination in these materials and corresponding solar cells. Previous studies indicated that energy and charge transfer occurs from small *n* to high *n* members in layered tin perovskites, the last of which is a critical process responsible for the generation of free carriers.^[^ ^134^, ^260^ ^]^ Quasi‐2D perovskites only allow the conduction of charges in regions where the inorganic octahedra are in contact, and inhibit the charge conduction across the insulating organic spacer cations. Therefore, the performance of the quasi‐2D tin perovskite‐based solar cells highly depends on the crystallinity, orientation, phase purity, and distribution of the quasi‐2D films. In the following sections we aim to cover the recent advancement in the field of quasi‐2D tin‐based perovskite solar cells, which are mainly based on RP and DJ perovskites. We discuss the role of the organic spacer cations, solvents additives, processing methods on the crystallographic behavior, defect properties, charge transport, and recombination, as well as solar cell performance with the aim to inspire future research.

#### 7.6.1 RP Quasi‐2D Sn‐Based Perovskite Solar Cells

We first introduce the quasi‐2D tin RP PSCs employing aliphatic monoammonium as spacer cations. Cao and co‐workers reported the first quasi‐2D tin PSCs using butylammonium as spacer cation.^[^ ^134^ ^]^ Their work revealed how the processing conditions influence the phase purity, orientation, and morphology of the quasi‐2D tin perovskite films. The perovskite precursor solution plays a major role in the final film quality. Making solutions by mixing stoichiometric amounts of metal halide and ammonium halide salts led to mixed layered tin perovskite phases, whereas making solutions from presynthesized quasi‐2D materials produced phase‐pure layered tin perovskite films. This result needs to be further analyzed due to the difficulties to obtain pure‐phase quasi‐2D films. The BA_2_MA_2_Sn_3_I_10_ films were reported to grow with the inorganic planes parallel to the substrate in case of DMSO solvent, and perpendicular to the substrate when DMF is used. The explanation behind the different direction of growth is reported in Section 5. The inferior orientation of the films where DMSO is used blocks the charge transport across the spacer cations in the direction perpendicular to the electrodes and causes significant bimolecular charge recombination. In the absence of additives, the BA_2_MA_3_Sn_4_I_10_ film featured a noncompact morphology and a high density of defects, which cause severe monomolecular charge recombination. By adding SnF_2_ as reducing agent and triethylphosphine soft Lewis base into the precursor solution, the authors obtained uniform and compact BA_2_MA_3_Sn_4_I_10_ film, which displayed less defects and exhibited slower free charge carriers decay dynamics. Consequently, the solar cells delivered a PCE of 2.53% for quasi‐2D PSCs using BA_2_MA_3_Sn_4_I_10_ as light harvesting layer, which showed superior stability in air compared to the 3D counterpart. The encapsulated quasi‐2D solar cells retained more than 40% of their efficiency after 4 months. The achievement of such a remarkable stability in quasi‐2D tin perovskite solar cells is attributed to the low‐lying VBM and the bulky and hydrophobic organic spacer cations used, which form a protecting layer inhibiting the reaction between the tin perovskite layer and water and oxygen.

Li et al. reported the effects of the chain length of aliphatic alkyl ammonium spacer cations on the growth and the structural stability of quasi‐2D RP phases, which employed butylamine (BA: CH_3_(CH_2_)_3_NH_3_^+^), octylamine (OA: CH_3_(CH_2_)_7_NH_3_^+^), and dodecylamine (DA: CH_3_(CH_2_)_11_NH_3_^+^)] as organic spacer cations.^[^ ^260^ ^]^ On the one hand, the longer organic spacer cations cause stronger quantum confinement effect, which enlarges the bandgap and energy barrier for the charge tunneling across the barrier layers. On the other hand, the longer spacer cations lead to disordered phase orientation and distribution of the quasi‐2D RP Sn‐based perovskite film. Therefore, organic spacer cations with shorter chain length are favorable for improving the d transport properties of the solar cells in terms of highly oriented grains and ordered phase distribution. The organic spacer cation with short alkyl chain (e.g. BA) forms an ordered protecting layer, which prevents the penetration of the oxygen and water into tin RP film and therefore prolonged the lifetime of the solar cells in air.

Xu et al. investigated the use of 5‐ammoniumvaleric acid (5‐AVA^+^) as the organic spacer cation in quasi‐2D tin‐based RP PSCs.^[^ ^261^ ^]^ In the absence of the additives, the device employing AVA_2_FA_4_Sn_5_I_16_, as light absorbers showed a PCE value of 4.19%, with a *V*_oc_ of 0.47 V, a *J*_sc_ of 15.6 mA cm^−2^, and FF of 57.2%. Such poor performance was attributed to the charge recombination in the presence of high density of defects in the as‐cast films, which suffer from the random orientation and bad film morphology. Adding appropriate amount of ammonium chloride (NH_4_Cl) as an additive led to vertically oriented tin‐based quasi‐2D perovskite film with superior compact morphology. This additive‐based approach suppressed the charge recombination by improving the charge transport and extraction in the solar cells, which delivered a PCE of up to 8.71%.

In the following, we discuss the quasi‐2D RP PSCs using aromatic monoammonium molecules as spacer cations. Liao et al. first fabricated high *n*‐member (*n* = 9) layered tin perovskite solar cells using PEA cation.^[^ ^160^ ^]^ Unlike the previous studies using low *n* member layered perovskite, PEA_2_MA_8_Sn_9_I_28_ reported in this study actually resembles much more 3D perovskites in terms of optical and electronic properties. The preferential vertical orientation of the inorganic layers of the PEA_2_MA_8_Sn_9_I_28_ forms a direct path facilitating charge transport and extraction. The device with an inverted planar architecture of ITO/NiO*_x_*/perovskite/PCBM/Al displayed a PCE of 5.94% with a *V*_OC_ of 0.59 V, a *J*_SC_ of 14.44 mA cm^−2^, and an FF of 69%. In addition, these devices retained their performance for over 100 h without encapsulation. The impressive stability of the layered perovskite system was attributed to the collective factors of the hydrophobicity of PEA passivating the grain boundaries, the compact film morphology, and the large decomposition enthalpy, which enhanced the resistance to oxygen. Previous studies indicated that aromatic spacer cations have higher dielectric constant over the aliphatic counterparts and reduces the binding energy of the excitons. A direct comparison of the performance between the PEA‐ and BA‐based PSCs made under the same conditions has been lacking in the literature, which leaves the puzzle whether the dielectric constant of the organic spacer cation plays a role in the charge generation and transport of the tin‐based quasi‐2D RP phase.

More recently, Li and co‐workers reported that phenyl ethyl ammonium chloride (PEACl) as an additive enables to grow pure phase quasi‐2D crystals of Sn‐perovskite with vertical orientation.^[^ ^262^ ^]^ PEACl acts as a barrier layer at the surface of the crystals to protect tin from oxidation. Consequently, the device prepared with PEACl showed an PCE (9.1%) with minor hysteresis.

To summarize, quasi‐2D tin‐based RP PSCs exhibited superior environmental stability compared to their 3D counterparts when hydrophobic organic spacer cations are used. The van der Waals force between the monovalent spacer cations is one of the weak points for the structure and poses challenges in further improving the structural stability of the tin RP phase. Enhancing the interactions between the organic spacer cations could be one parameter to be considered from chemical synthesis point of view to enhance the structural stability of the RP phase. It is necessary to further optimize the processing conditions, additives, and the structure of the organic spacer cations to achieve ideally vertical orientation, good phase purity, and ordered phase distribution of the Sn‐based RP film to realize higher performance solar cells. The vast versatility in structure of the organic spacer cations could further advance the efficiency and stability of tin‐based RP PSCs.

#### 7.6.2 DJ Quasi‐2D Sn‐Based Perovskite Solar Cells

Recently, quasi‐2D tin‐based DJ phases have also been applied to perovskite solar cells due to their weaker quantum confinement and higher structural stability in the absence of the van der Waals interaction. Chen et al. first reported the quasi‐2D DJ Sn‐based perovskite solar cells employing 4‐AMP as spacer cation.^[^ ^263^ ^]^ The quasi‐2D DJ phase with *n* varied from 1 to 4 clearly show red‐shifted absorption and PL emission peak with increased *n* value, and the average *n* = 4 film has an optical bandgap of 1.47 eV, which is very suitable for solar cell applications. The authors prepared an HTL‐free device using (4‐AMP)MA_3_Sn_4_I_13_ as light harvesting layer, which exhibited a PCE of 4.22%, and was stable under 1 sun illumination in a N_2_ atmosphere at 45 °C for 100 h. More recently, Li et al. investigated a series of low‐dimensional DJ perovskites using 1,4‐butane diamine (BEA) as organic spacer cations.^[^ ^264^ ^]^ The BEA ligand stabilized the low‐dimensional perovskite structure by enlarging the formation energy up to 106 J mol^−1^, which in turn inhibits the oxidation of Sn^2+^. The (BEA)_2_FA_2_Sn_3_I_10_ film exhibited a fast charge transfer across the quantum well layers from low *n* to high *n* members due to weakened carrier localization, which leads to a carrier diffusion length over 450 nm for electrons and 340 nm for holes, respectively. The solar cells fabricated with (BEA)_2_FA_2_Sn_3_I_10_ delivered a PCE of 6.43% with negligible hysteresis, which was remarkably higher than the 3D‐based counterpart due to much lower trap density. Moreover, the (BEA)_2_FA_2_Sn_3_I_10_ devices were more robust against oxidation, illumination, and humidity than the 3D devices due to higher structural stability. It is worth mentioning that the (BEA)_2_FA_2_Sn_3_I_10_ film in this study exhibited significant amorphous components, which are possibly associated with the reduced assembling capability of the rigid diammonium cation.

These studies demonstrated that tin‐based quasi‐2D DJ phases featured with large organic diammonium cations can enhance the stability of the solar cells by efficiently blocking water and oxygen from reacting with the perovskite layer. Research in this field is still at early stage and the performance of the tin‐based quasi‐2D DJ PSCs is lagging much behind those of the RPP‐based counterparts. The possible factors that are responsible for such poor performance include nonideal crystallinity, random or parallel orientation, low phase purity, and disordered phase distribution of the quasi‐2D DJP. It is still unclear if these features are consequences of the use of diammonium cation or if an optimization of the processing techniques and the structure of the organic spacer cations can allow in the long run to exploit the positive feature of this class of low‐dimensional systems.

## 8 Conclusion

In this review, we scrutinized the large body of work that has been reported on Sn‐based metal halide perovskites, where we discussed the structural and optoelectronic properties of a broad range of materials, including 3D, hollow, and low‐dimensional perovskite systems. We provided a detailed discussion of the nature of crystallographic phases and discussed the effect of the connectivity of the inorganic octahedra on the band structure, which are summarized by the following four key points. i) 3D perovskites exhibit several phase transitions going from high symmetry structures to lower symmetry structures with decreasing temperature, introducing distortions in the octahedral framework. ii) The band edges are mainly composed of tin halide orbitals, such that distortions to the cubic connectivity reduces the bandwidth of the electronic bands, thereby increasing the bandgap and the effective masses of these compounds. iii) Hollow and lower dimensional structures follow much of the same key points described above, as these structures can be deduced from the 3D parent structure. iv) All these structures show excellent compositional versatility, enabling a further tuning of the bandgap. The poor stability of Sn‐based perovskites remains a big challenge, and several strategies emerged to address this issue starting from reviewing its defect chemistry. It is our view that the introduction of reducing agents and large organic cations in the precursor solutions have been important milestones for improving both the crystalline quality of the thin films and the device performances. We strongly believe that an in depth understanding and consequent controlling of the crystallization mechanisms are decisive for advancement of the field, as it was demonstrated that a better crystallinity of the Sn‐based perovskite film results both in better efficiency and stability. Encouragingly, we have shown that the search for better stability and higher PCE devices are not necessarily mutually exclusive. To date, many of the high efficiency solar cells use additives to further increase the PCE and the stability of the devices, and we believe that continuous positive developments in the field will be reported in the near future. Finally, low‐dimensional perovskites have been essential for solar cell developments, but their fundamental properties have largely been limited to their structural characterization only. Nonetheless, it remains unclear how and when exotic structures are formed, alongside the question why Sn‐based structures show polymorphs with the same chemical composition. In addition, many of their photophysical properties are unresolved, which could both open up new photovoltaic applications as well as resolve remaining controversies in Pb‐based perovskites. Above all, we are convinced that the initial promises of this lead‐free material are justified by the recent improvements in device efficiency and that future innovative strategies will make one day the use of Sn‐based perovskites commercially relevant.

## Conflict of Interest

The authors declare no conflict of interest.
